# Growing recyclable and healable piezoelectric composites in 3D printed bioinspired structure for protective wearable sensor

**DOI:** 10.1038/s41467-023-41740-6

**Published:** 2023-10-14

**Authors:** Qingqing He, Yushun Zeng, Laiming Jiang, Ziyu Wang, Gengxi Lu, Haochen Kang, Pei Li, Brandon Bethers, Shengwei Feng, Lizhi Sun, Peter Sun, Chen Gong, Jie Jin, Yue Hou, Runjian Jiang, Wenwu Xu, Eugene Olevsky, Yang Yang

**Affiliations:** 1 Department of Mechanical Engineering, San Diego State University, San Diego, CA 92182 USA; 2 Alfred E. Mann Department of Biomedical Engineering, Viterbi School of Engineering, University of Southern California, Los Angeles, CA 90089 USA; 3 College of Materials Science and Engineering, Sichuan University, Chengdu, 610064 China; 4 The Institute of Technological Sciences, Wuhan University, Wuhan, 430072 China; 5 Department of Civil and Environmental Engineering, University of California, Irvine, California, CA 92697 USA; 6 Grossmont College, 8800 Grossmont College Dr, El Cajon, CA 92020 USA; 7 Canoo Technologies Inc, Torrance, CA 90503 USA

**Keywords:** Composites, Mechanical properties, Organic molecules in materials science

## Abstract

Bionic multifunctional structural materials that are lightweight, strong, and perceptible have shown great promise in sports, medicine, and aerospace applications. However, smart monitoring devices with integrated mechanical protection and piezoelectric induction are limited. Herein, we report a strategy to grow the recyclable and healable piezoelectric Rochelle salt crystals in 3D-printed cuttlebone-inspired structures to form a new composite for reinforcement smart monitoring devices. In addition to its remarkable mechanical and piezoelectric performance, the growth mechanisms, the recyclability, the sensitivity, and repairability of the 3D-printed Rochelle salt cuttlebone composite were studied. Furthermore, the versatility of composite has been explored and applied as smart sensor armor for football players and fall alarm knee pads, focusing on incorporated mechanical reinforcement and electrical self-sensing capabilities with data collection of the magnitude and distribution of impact forces, which offers new ideas for the design of next-generation smart monitoring electronics in sports, military, aerospace, and biomedical engineering.

## Abstract (web summary)

Smart monitoring devices with integrated mechanical protection and piezoelectric induction are limited. Here, the authors report a strategy to grow piezoelectric Rochelle salt crystals in 3D-printed cuttlebone-inspired structures to produce smart monitoring devices with integrated mechanical protection and electrical sensing capability.

## Introduction

Recently, bioelectronics that are lightweight, robust, and smart sensors for sports and gerontology have been extensively studied and developed due to the need for integrated sensing and protection^1–6^. Nonetheless, the smart sensing function and high-strength protection do not go hand in hand. For example, the most-developed wearable electronics for bio-monitoring were based on soft piezoelectric materials or flexible printed circuit boards, which lack protective capability^7–9^. In contrast, advanced armors were assembled with strong organic fibers, metals, or inorganic ceramics and could not work as sensors. Combining and integrating the sensing and protection functions to fabricate multifunctional wearable sensors are in demand for future applications, such as sports vests, space armor, and elderly protective gear, which require new manufacturing strategies to achieve^10–12^.

The basis for the structural design of multifunctional sensors is inspired and built by translating the specific microstructures. Bio-structures in nature have evolved over thousands of years and attracted much attention in the design of functional structural materials for various applications owing to their low density and high strength^13–16^. One interesting example is the cuttlefish that has a stiff cuttlebone structure to sustain high water pressure in deep-sea areas^17^. The key point of cuttlebone’s excellent protective performance lies in its unique chambered wall-septa microstructure, which is able to achieve high stiffness and energy absorption under high-pressure circumstances^17^. Furthermore, these wall-septa microstructures also provide high porosity inside the cuttlebone which is an excellent model for multifunctional sensor design^18^.

The first challenge comes from the manufacturing process of the high-porosity cuttlebone structure being used as a unique base model for smart sensing materials (e.g., piezoelectric composites). The traditional fabrication for piezoelectric composite (such as 1–3 or 2–2 composites) includes the dicing-and-filling technique and molding process^19,20^. However, complex microstructure design hinders the further development of these conventional fabrication techniques. Additive manufacturing, also known as 3D printing technology, has become an effective way to overcome these barriers and fabricate complex bio-inspired structures with reinforced mechanical and electrical properties^21–24^. Additionally, in cuttlebone-inspired structures, the empty chamber space between each wall-septa structure can be utilized to fill specific functional materials for sensing applications. Traditional piezo-based materials are hard materials, which are difficult to manufacture in an irregular shape^25^. Rochelle salt crystal (RS) is a crystalline solid with expected piezoelectric and ferroelectric properties, which can be easily synthesized, melted under relatively low temperatures (70–80 °C), and recrystallized after cooling. Besides, RS crystal is an eco-friendly material that has been used for decades^26^. Hence, it shows great potential for utilization of RS with ease-manufacturing, healing and sustainable capability for sensing applications^27^.

Herein, we directly grow RS crystal in the cuttlebone-inspired structure for the smart monitoring device with integrated mechanical protection and electrical sensing capability. The cuttlebone structure was achieved by a 3D printing method using photocurable resin to grow RS crystal composite. The synthesis and the mechanism of piezoelectric performance of RS crystal in the 3D-printed cuttlebone structure, 3D-printed Rochelle salt cuttlebone composite (RSC), was systematically investigated. The printed composites with growing crystals displayed remarkable piezoelectric and mechanical performance, as well as excellent healable and recyclable properties. Moreover, the smart array armor as well as the knee pad based on the 3D-printed-RSC can be achieved for the detection of the location and magnitude of the force experienced by the wearers. These results establish the foundation for new-generation of smart monitoring electronics for various applications, such as sports, medicine, military, and aerospace.

## Results and discussions

### Cuttlefish bone-inspired 3D-printed RS piezoelectric composites

**Fig. 1:**
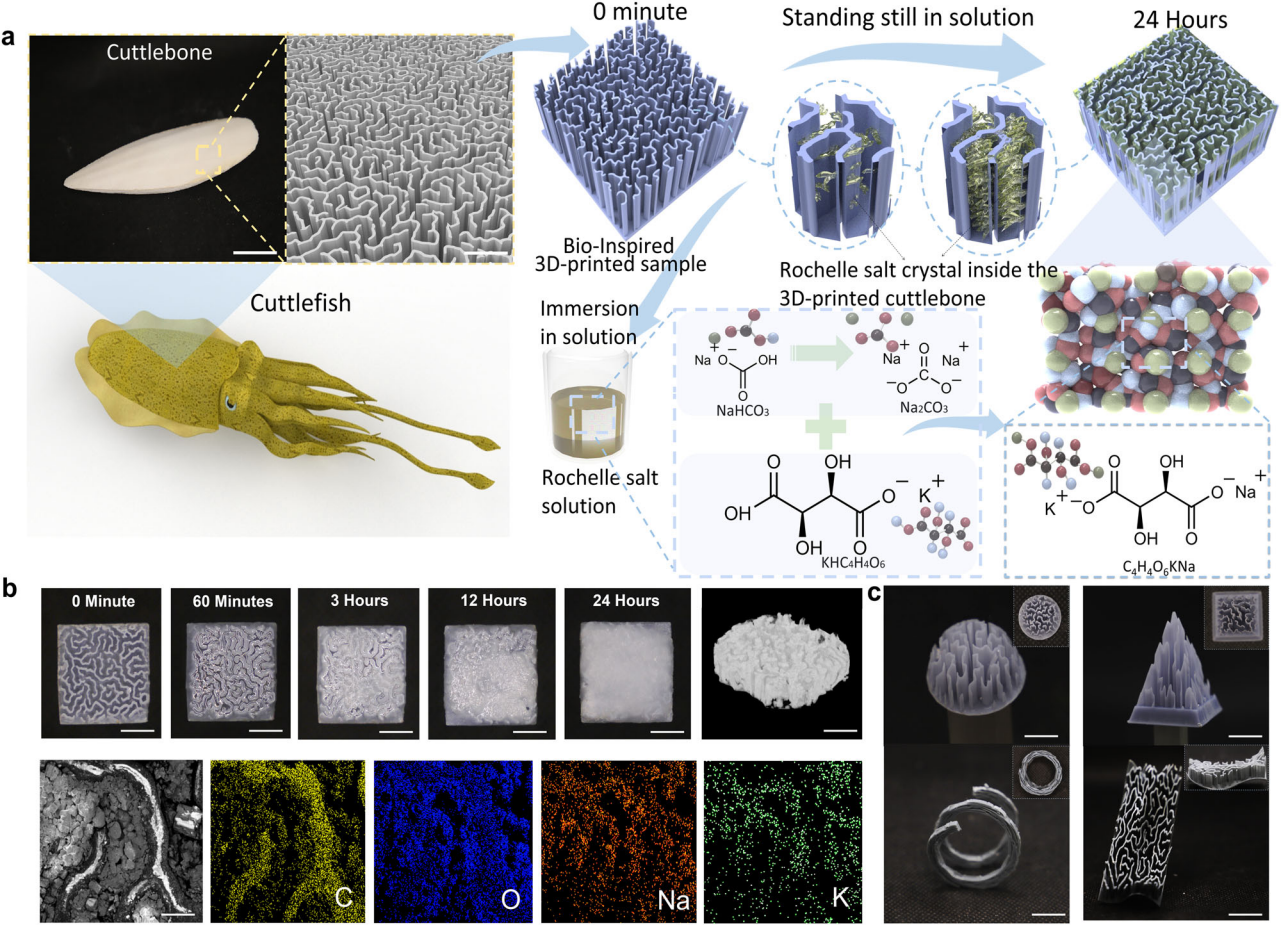
Design and crystal growth process of the 3D-printed-RSC. **a** Schematic diagram of bio-inspired 3D-printed cuttlefish bone structure and RS crystal growth process (cuttlebone photo scale bar, 40 mm; cuttlebone structure scale bar, 100 µm); **b** The picture of crystal growth in 3D-printed structure at different times (scale bar: 5 mm); CT scan picture of the sample (scale bar: 5 mm), and EDX elemental analysis of sample (scale bar: 500 µm); **c** Photos of multiple 3D-printed artificial cuttlebone complex structures, demonstrating the design flexibility of this 3D printing method (scale bar: 5 mm).

Natural cuttlefish have evolved a strong bone structure that enables them to withstand high pressure in deep sea areas. The wall-septa microstructure in cuttlefish bone plays a vital role in being rigid and damage-resistant properties (Fig. 1a). Inspired by cuttlefish, a 3D-printing method was applied to build the artificial cuttlefish bone structures. The schematic of the fabrication process for producing the 3D-printed-RSC is shown in Fig. 1a. Here, stereolithography was employed as the method of 3D printing due to its high resolution (Supplementary Fig. 1). The printed sample was merged into the synthesized RS medium for the growth and attachment of piezoelectric crystals. The RS crystal filled all the gaps that exist between the walls of the microstructure inspired by cuttlefish bones.

The manufacturing process of the piezoelectric composite mainly relies on RS growth within the 3D-printed cuttlebone-inspired structure. According to previous studies, dicing-and-filling technology for 1–3 and 2–2 composites is one of the most common fabrication methods for piezoelectric composites^28^. For example, ref. ^29^ investigated the use of a 3D-printed barium titanate honeycomb structure filled with epoxy to produce a piezoelectric composite. However, this method requires subsequent high-temperature sintering, which is time-consuming and sacrifices performance^30^. The study of growing piezoelectric crystals into printed polymer structures demonstrates a new way to fabricate piezoelectric composites with complex shapes. The detailed process of growing crystals into the printed structure is demonstrated in Fig.1a and explained in the methods. Fig. 1b illustrated the photos of the RS crystals growing process under each specific time over 24 h, scanning electron microscopy (SEM) (Supplementary Fig. 2), and computerized tomography (CT) scanning, respectively (Supplementary Fig. 3). The microstructure and composition of the 3D-printed-RSC were further examined using SEM and energy dispersive X-ray spectroscopy (EDS) shown in Supplementary Fig. 4a. Based on the results, it is evident that the carbon elements are concentrated in the 3D-printed cuttlebone by virtue of the light-curing resin. As the RS crystals grow in the middle of the skeletal gaps, oxygen, potassium, and sodium are mainly concentrated. EDS Spectrum in Supplementary Fig. 4b depicts the expected major elements such as Carbon from the 3D-printed cuttlebone, Oxygen, potassium, and sodium from the RS crystal. Supplementary Fig. 4c illustrates the weight ratio and atomic ratio of four different elements in the tested composite. The top view of the CT scanned photo is shown in Supplementary Fig. 3, which illustrates the distribution of the 3D-printed structure and RS crystal growth. The growth of the crystal is uniform and dense, and it essentially fills the voids. Besides, the applied 3D printing method is also capable of being used to obtain the complex transformations of the cuttlefish bone structure, as shown in Fig. 1c. And 3D-printed-RSC with different scales are also exhibited in Supplementary Figs. 5 and 6.

### Measurement of piezoelectric response of 3D-printed-RSC

**Fig. 2:**
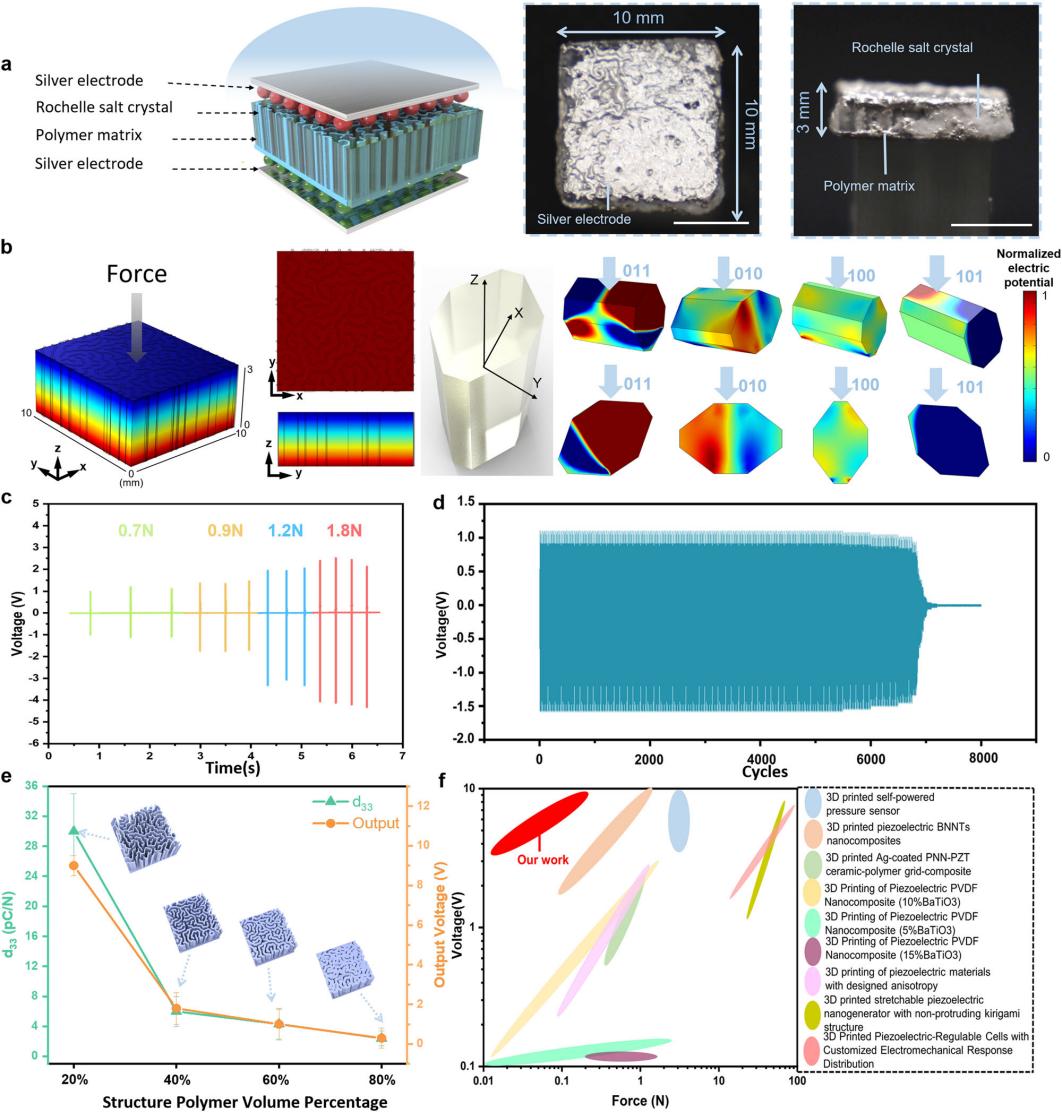
Piezoelectric performance of 3D-printed-RSC. **a** Illustration of the tested 3D-printed-RSC sample electrodes and photos of vertical free fall piezoelectric performance test process using weights (scale bar from left to right, 5 mm, 5 mm, 25 mm); **b** Piezoelectric FEM simulation of the 3D-printed-RSC sample, simulations were conducted in four different directions of piezoelectric individual RS crystals; **c** Piezoelectric output at different frequencies corresponding to different force magnitudes; **d** The output voltage over 8000 cycles cyclic impact test under 2 Hz frequency; **e** Voltage output and piezoelectric coefficient comparison line for the identical test conditions corresponding to the polymer ratio of different 3D-printed-RSC. Error bars represent standard deviation(*n* = 10); **f** Comparison of output voltage and force of 3D-printed-RSC with other structures^47–53^.

The composite (10 mm × 10 mm × 3 mm) was laid flat on a glass substrate with a contact angle (CA) between the material and the table of 0˚ (Fig. 2a). Composites with 24 h growth of RS crystal inside the printed cuttlebone-inspired polymeric structure with different volume ratios were produced and used. Stainless steel with weight loadings (1 g–20 g) were applied to the 20% composites along the vertical direction, and accordingly, significant differences in the output voltage were observed for various weight loadings (Supplementary Fig. 7). Here, the output voltage of the 20% 3D-printed cuttlebone structure composite is shown in Supplementary Fig. 8, with the highest output performance compared to the other fabricated composites when CA is 0˚. Especially, dropping a 20 g weight at the distance of 50 mm directly above the sample, the output of the composite could reach 8 V_peak–peak_ owing to the contribution of its high-volume crystal ratio.

Additionally, cyclic tests were conducted to obtain more precise piezoelectric performance data. Supplementary Fig. 9 shows the motor-driven cyclic impact test set-up. The motor drives the rotation of the rocker arm, and the frequency as well as force level are adjusted by changing the motor input voltage. The bottom of the installation pulley allows the distance between the tested 3D-printed-RSC samples to be easily adjusted. For the purpose of obtaining the output voltage signal, the positive and negative terminals of the 3D-printed-RSC are connected to the signal collector. To obtain accurate values of the magnitude of the forces on the tested sample, we apply the thin film force sensor as shown in Supplementary Fig. 9a. From 1 Hz to 4 Hz, Fig. 2c illustrates how voltage is generated by continuous impact on a 3D-printed-RSC. The sensitivity of the composite can be observed in Supplementary Fig. 9b according to the peak-to-peak output voltage under varying forces. Fig. 2d and Supplementary Fig. 10a demonstrates the 8000 cycles cyclic impact test output voltage. It indicates that the 3D-printed-RSC devices perform well and maintain a stable output voltage from 0 to 6800 impact cycles. A fracture was observed in the sample at about 6800 cycles with RS crystal detachment. A complete failure occurred at approximately 7000 cycles. Thus, the cycling 7000 uses as an athlete’s smart armor are sufficient for a single sport season of use^31^. Supplementary Fig. 10b, c display the output voltage at the beginning and the end of the cyclic impact test. In the event that the composite has been damaged (Supplementary Fig. 11), the 3D-printed-RSC can be repaired in accordance with its healable and recyclable characteristics, so that it can be used for a longer period of time, which will be discussed in the later section.

The effective piezoelectric coefficient d_33_ with an average value of 30 pC/N is obtained for the 20% composites (Fig. 2e). Supplementary Fig. 12 displays the setup of *d*_33_ measurement. Furthermore, Supplementary Fig. 13 provides images of RS growth in different volume ratios of 40%, 60%, and 80% 3D-printed cuttlebone structures samples at various growth times. And Supplementary Fig. 14 illustrates voltage outputs in response to different RS crystal growth times in 20% composites. Consequently, the comparison was plotted between the force applied to the composites and the corresponding voltage generated by the other 3D-printed piezoelectric materials (inset image in Fig. 2f and Supplementary Tab. 1). It shows that our fabricated 3D-printed-RSC shows excellent piezoelectric output voltage with a relative low impact force.

Moreover, the converse piezoelectric effect was also achieved by getting the displacement under the input voltage. In order to measure out-of-plane displacement modules and phase changes in the tested samples^32^, the actuating conditions (−10V to 10 V sinusoidal input voltage from 0 Hz to 20 KHz) were applied to the samples that were placed in a laser vibrometer (MSA-500 from Polytec) (Supplementary Fig. 15). Supplementary Fig. 16a illustrates the normalized out-of-plane displacement distribution, in which the red area exhibits a greater displacement change than the green area, corresponding to the locations of the filled RS crystals and the 3D-printed Cuttlebones. Based on the dynamic response of the 3D-printed-RSCs in the frequency domain, it was confirmed that the composites exhibited a converse piezoelectric behavior (Supplementary Fig. 16b). The statistical displacement increased with the applied voltage^33^, and we performed a linear fit to the resulting curve, and the slope of the fitted curve is the converse *d*_33_ (Supplementary Fig. 17). The results in the direct and converse *d*_33_ values remain essentially the same. For the purpose of eliminating the factor of output voltage due to friction between different materials, we compared three materials in each 3D-printed Cuttlebone separately. The results of Supplementary Fig. 18 indicate that, as compared to the output voltage signal of the 3D-printed-RSC, the epoxy and silicone rubber samples did not exhibit any significant voltage output after being hit by the weights. Furthermore, to ensure that the measured signal is the actual result of piezoelectric responses, a voltage from switching polarity tests is measured, as shown in Supplementary Fig. 19. Upon reversing the polarity of the fabricated composites^34^, the shapes of the electrical output curves are reversed, confirming the piezoelectric response.

The structural finite-element-method simulation using COMSOL Multiphysics demonstrates that a piezoelectric potential distribution occurs when the weight falls perpendicular to the composite (Fig. 2b). A factor *d*_km_ [m/V] is commonly used to convert from mechanics to electricity, namely from stress-strain to potential difference and electric field strength^35^. A reduced matrix notation dKM (Supplementary Fig. 20) is commonly used to express both direct and reverse piezoelectric effects^35,36^. The orientation of crystals in simulation is determined by the coordinate system selected, to simulate the piezoelectric potential distribution of different crystal orientations, reference frame x_1_, x_2_, and x_3_ were specified in simulation as X, Y, and Z axes respectively for each single RS crystal. An individual crystal has four pairs of force surfaces along the z-axis, and the normalized electrical potential difference calculated in simulation is illustrated in Fig. 2b. Although RS crystal has a strong piezoelectric effect, the piezoelectric properties of every single RS vary with reference to the crystalline axes. An RS crystal is composed of three axes, the electrical axis (x-x’), the mechanical axis (y-y’), and the optical axis (z-z’). The slices are cut into different shapes according to the specific application, such as expander or shear vibration plates^37^. In our study, since no cut was conducted, the effective *d*_33_ of the final structure is the combination of three shear piezoelectric constants. The measured effective *d*_33_ is approximately ~30 pC/N, which fits within the theoretical range. The coupling factor of the 3D-printed-RSC is calculated through testing the impedance spectrum in Supplementary Fig. 21. Furthermore, the piezoelectric responses of the pure RS film are also analyzed and illustrated in Supplementary Fig. 22.

### Mechanical properties test of 3D-printed-RSC

**Fig. 3:**
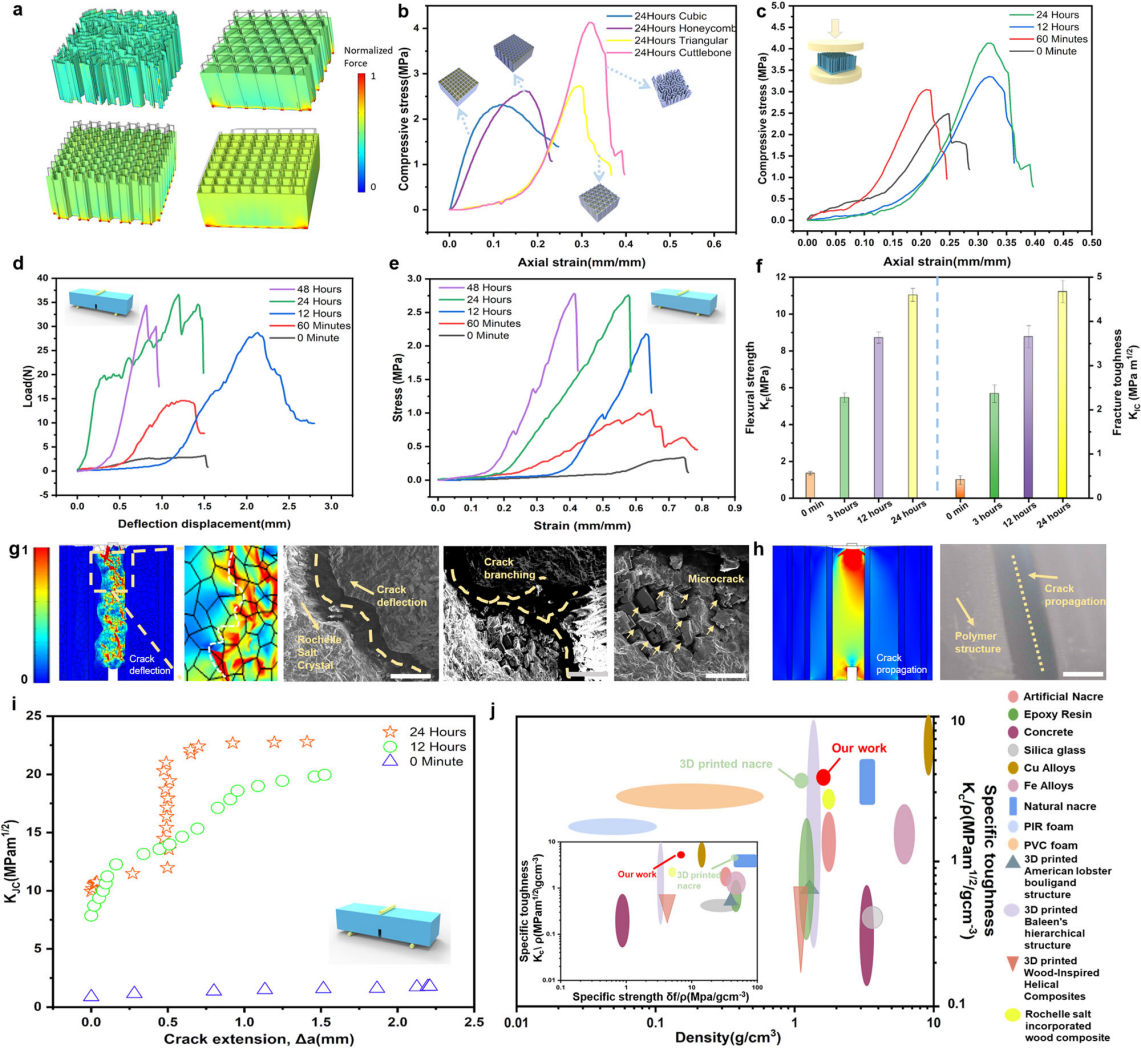
Studies of mechanical properties of 3D-printed-RSC. **a** FEM simulation of the compressive load of four different structures; **b** Comparison of compressibility of RS crystal growth in various structures for 24 h; **c** Comparison of compression properties of different RS crystal growth time inside the 3D-printed cuttlebone structure; **d** Compression force versus resistance change for various RS crystal growth time inside the 3D-printed cuttlebone structures; **e** Comparison of stress versus strain of the 3D-printed cuttlebone structures with different RS crystal growth time; **f** Comparison of fracture strength (K_F_) and fracture toughness for crack initiation (K_IC_) of the 3D-printed cuttlebone with different RS crystal growth time. Error bars represent standard deviation (*n* = 10); Simulations of stress distribution by COMSOL Multiphysics for the 3D-printed-RSC (**g**) (inset shows SEM images of the related fracture surfaces, scale bar from left to right, 400 µm, 400 µm, 150 µm) and 3D-printed cuttlebone (**h**) (inset shows microscope photo of the related fracture surface, scale bar 500 µm); **i** R-curves of the 3D-printed-RSC for different growth time; **j** Comparison of specific toughness and density of the 3D-printed-RSC with other works (inset shows the comparison of specific strength and specific toughness of the 3D printed-RSC with other works)^21,26,38,43,54–63^.

To study the mechanical properties of the RS crystals grown in the 3D-printed cuttlebone structure, three common mechanical structures with the same dimensions and materials (Cubic, Honeycomb, and Triangular) are 3D-printed for comparison. When subjected to the same compressive force, the composite with cuttlebone structure exhibits the least deformation and uniform force distribution compared to the composites with other three structures. Then, RS crystals were grown overnight in the printed samples. Fig. 3a illustrate the comparison of simulated mechanical properties among the Cubic, Honeycomb, Triangular, and Cuttlebone structures in the uniform color bars. The cuttlefish bone structure exhibits the best mechanical properties and expected piezoelectrical sensing abilities in the same conditions among all these structures (Fig. 3b and Supplementary Fig. 23). Furthermore, the compressive stress-strain curves of the composites with different growing times are demonstrated (Fig. 3c) with a buffer downtrend. In addition, a bar chart of the maximum load for different RS crystal growth rates in 3D-printed cuttlebone samples is demonstrated in Supplementary Fig. 24. With the wall-septa structures of cuttlebone structure, the wavy septas can limit damage to chambers in the middle chamber between walls under high pressure, contributing an excellent mechanical property. A 3D-printed cuttlebone-based structure without crystal shows a high maximum load of 180 N. Additionally, with RS crystal growing inside the structure to fill every free space, the maximum load of the composite is gradually increased.

To further study the toughness of the composites, the standard three-point bending tests were applied for 3D-printed-RSC growing at different times (Fig. 3d). The results demonstrate that the sample with pure polymer structure exhibits a nearly linear elastic response before an entire failure happens, showing crack propagation on the fracture surface. Composites with crystal growth for 1, 12, and 24 h show crack deflection, which is enhanced by these crack suppression mechanisms, resulting in increased toughness. To study the reinforcement mechanisms, such as structural integrity and reliability of composites with crystal growing at different times, fracture toughness is analyzed, which is one of the most crucial mechanical properties of interest as it is defined as the capacity of a material with cracks to resist fracture (Fig. 3f, and Supplementary Fig. 25a)^38^. In comparison, the increasing growing time of the crystal led to a related increment of the fracture toughness for crack initiation (K_IC_). Significantly, the results show that the K_IC_ of composites with crystal growing for 24 h has the highest value of 4.67 MPa m^1/2^, which is enhanced by 1044.6% compared with the pure polymer structure. The crack branching and deflection between the RS grain boundary was observed from both experimental and simulation results, this crack suppression mechanism will absorb energy and increase the fracture toughness (Fig. 3g) compared to the crack propagation in pure polymer structure (Fig. 3h). The damage is not only confined to the crack tip in printed composites with crystal but also is widely spread ahead of the growing crack by deflecting microcracks following the direction of crystal growth in the composite (Fig. 3g).

Moreover, the three-point flexural tests were implemented to study the flexural strength of the proposed samples (Supplementary Fig. 25b)^39^. The stress-strain curves of the pure polymer sample and composites with crystal show a sharp drop behavior (Fig. 3e). As shown in Fig. 3f, the flexural strength (K_F_) of the composite, which describes as a material’s stress just before it yields in a flexural test^39^, increases significantly with crystal growth inside the composite. Interestingly, the K_F_ of the composites with 24 h of growing crystal (11 MPa) is as much as 8.4 times that of pure polymer structures (1.3 MPa).

Furthermore, based on our previous studies, the fracture toughness (K_JC_) will change with the crack extension (△a), thus plotting a characteristic crack resistance curve (R-curve) owing to the increase of K_JC_ with △a^38^. To quantitatively analyze the changes of K_JC_ with the crack extension (Δα), the J-R curve approach was applied (Fig. 3i). Similar to most of the bioinspired toughness structures^1,38^, the composites with crystal growth exhibit extensive rising R-curve behaviors. And the composite with 24 h of crystal growth has a higher R-curve than the composite with 12 h of crystal growth, demonstrating their increasing resistance to fracture during the crack growth due to more crystal growth inside the composite. Nonetheless, no R-curves were observed for the pure polymeric structures due to the crack propagation. Additionally, the specific toughness, specific strength, and density of the proposed composite are comparable to other natural structures and 3D-printed structures (inset image in Fig. 3j and Supplementary Tab. 2). These results strongly demonstrate that our proposed composites exhibit excellent mechanical properties with high toughness and strength due to the design of bio-inspired cuttlebone structure with the RS crystal growth inside. It also provides us with a better understanding of how the growing crystal is capable of absorbing energy and sustaining subcritical cracking. With the expected piezoelectric sensing response and mechanical properties, it brings more feasibility for us to develop smart monitoring armor and multifunctional sensors with the capability of high toughness and sensing.

### Recycling and healing behavior of 3D-printed-RSC

**Fig. 4:**
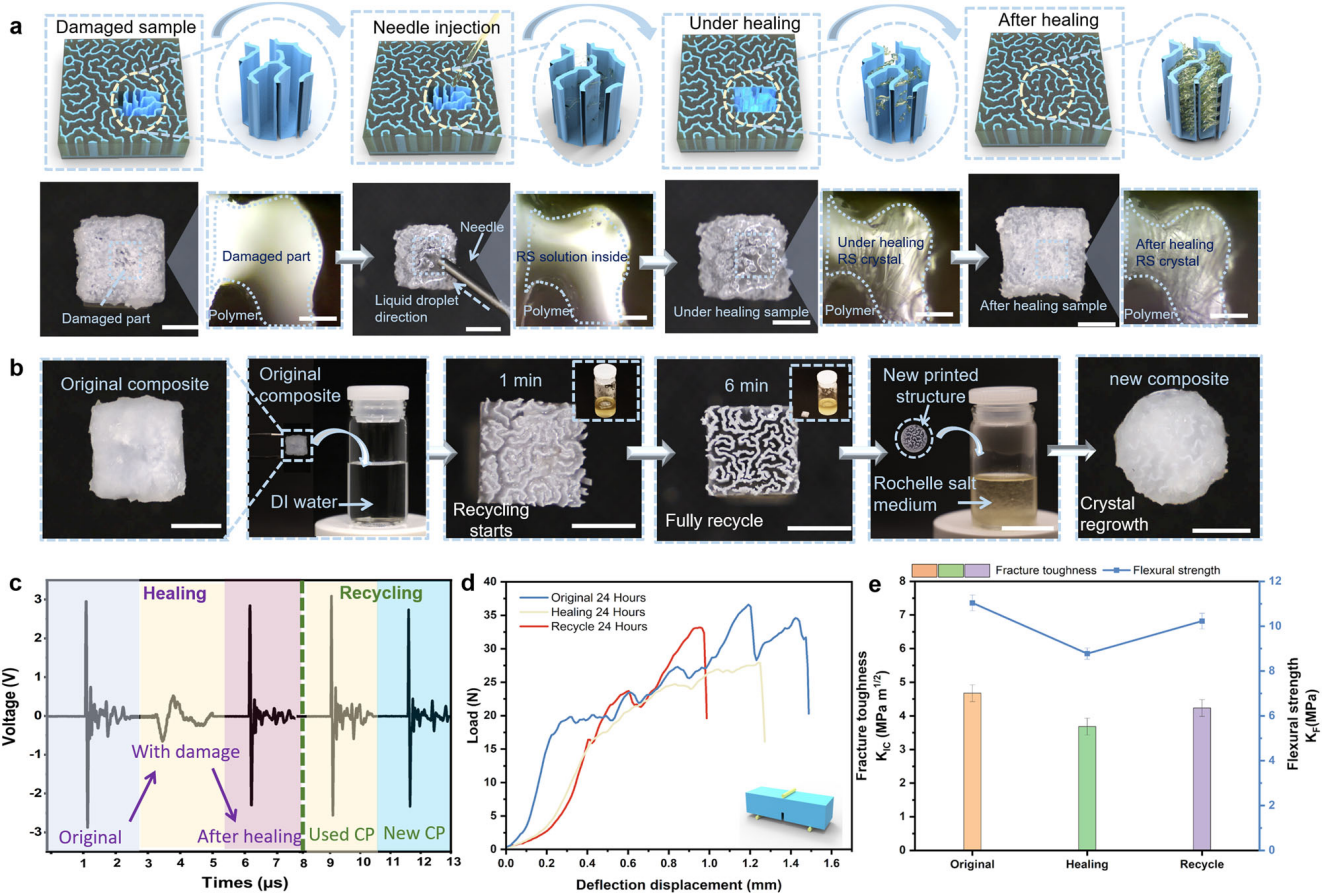
Recycling and healing behavior of 3D-printed-RSC. **a** Schematic diagram and photos of the process of repairing the broken 3D-printed-RSC sample by dropping the RS solution through a syringe (photos scale bar, 5 mm; microscope photos scale bar, 0.2 mm); **b** The photos of the process for 3D-printed-RSC recycling (scale bar, 7 mm); **c** Comparison of the piezoelectric response of the original 3D-printed-RSC samples with samples after recycling and healing; **d** Compression of force versus resistance change for original, healed, recycled samples, respectively; **e** Comparison of fracture toughness (K_IC_) and flexural strength (K_F_) among original, healed, recycled 3D-printed-RSC samples. Error bars represent standard deviation (*n* = 10).

For the applications in impact monitoring devices, the crystals could be damaged during usage as a result of the continuous impact, and the repairability of the composite was conducted. Heating the RS crystal can cause the mature solid crystals to dissolve and revert to a liquid crystal solution, leading to excellent recyclability and sustainability of the composite. After damage, the RS solution was injected into the damaged area and left at room temperature for 24 h (Fig. 4a). The microscope images show that the crystal grows well, and the damaged area is well repaired. In Fig. 4b, the recycling process of the prepared composite is demonstrated. A 3D-printed-RSC is first soaked in the heated DI water. After immersing for 6 min, the grown RS crystal inside the composite structure is completely dissolved in DI water and form the heated RS solution. Following that, the polymeric matrix structure is immediately separated from the recycled solution, and a new 3D-printed pure polymeric structure is used to immerse into the recycled solution to grow the RS crystal for a new piezoelectric composite. The mechanical and piezoelectric performance of the repaired and recycled samples were both tested. As shown in Fig. 4c, the elastic response and the amplitude of output voltage for the new composite are comparable to the original composite, achieving 95% of the original composite performance. We further compared the mechanical properties of the original composites and the healed composites, as well as the recycled composites, by applying standard three-point bending tests. In Figs. 4d and 4e, the K_IC_ of the healed and recycled composites recovered and reached 78.7% and 90.5% of the K_IC_ of the previous original composite, respectively. Moreover, the K_F_ of the healed and recycled composites achieved recovery percentage of 79.5% and 92.6%, compared to that of the previous original composite. These results illustrated that the recycled RS crystal can be well repaired and reused during utilization. And it demonstrated that RS piezoelectric composite is able to achieve excellent sustainability, thereby enriching the wide applicability and practicability for the new generation of smart armor.

### Applications of the composites for football athlete smart detection enhanced protective armor

**Fig. 5:**
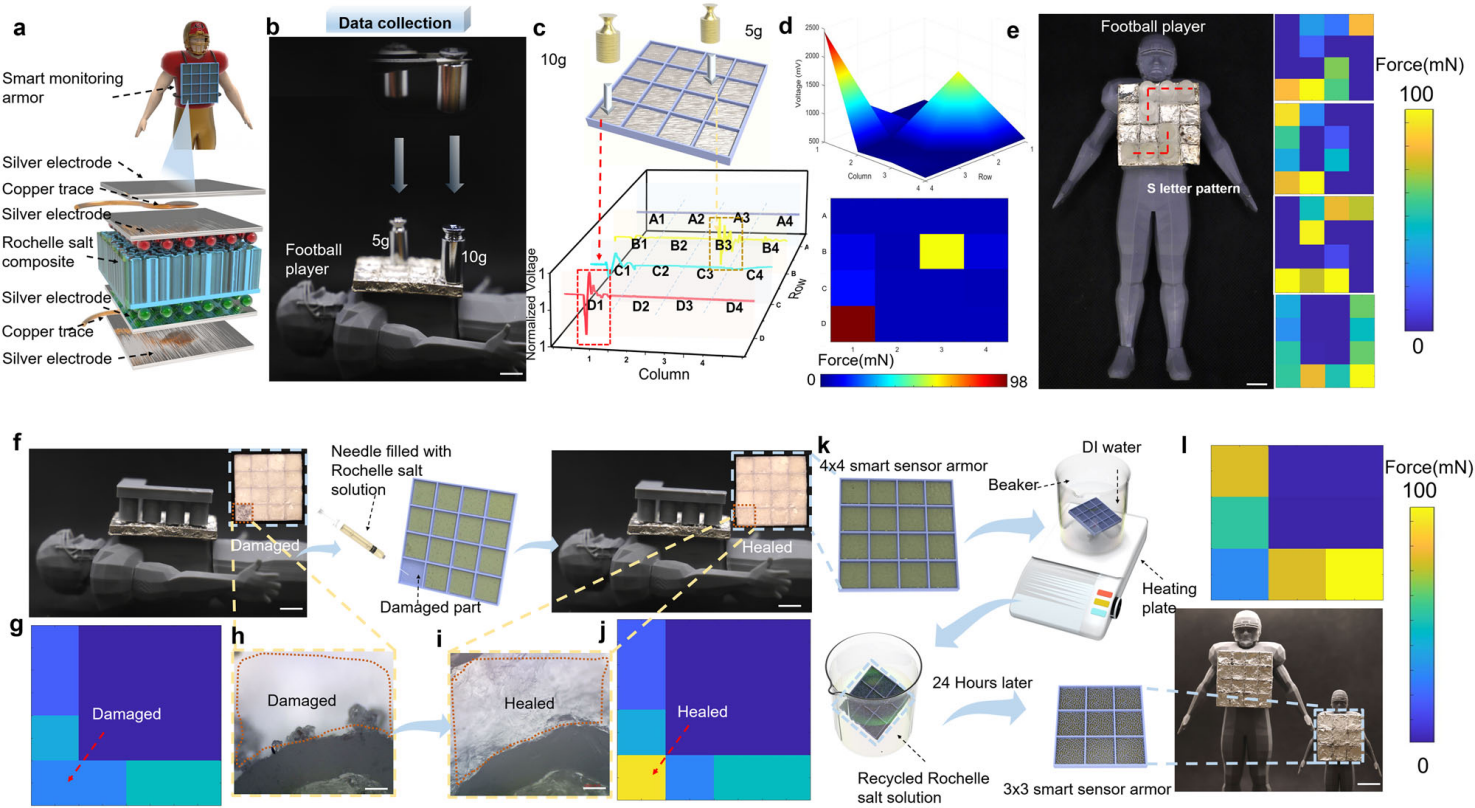
3D-printed-RSC for smart armor with enhanced protection. **a** Illustration of testing 3D-printed-RSC smart monitoring armor element electrodes; **b** Data collection using 5 g and 10 g weights to impact the 3D-printed-RSC smart monitoring armor (scale bar, 10 mm); **c** Voltage output waveform of the 16 elements corresponding to the impact and (**d**) the force distribution derived from the voltage analysis by MATLAB; **e** “SDSU” patterns can be derived from pressure detection distribution using MATLAB (scale bar, 10 mm); **f** Comparison of damaged smart monitoring armor before and after healing (scale bar, 10 mm). The enlarged images (**h**) and (**i**) compare the damaged and healing parts (scale bar, 0.2 mm); **g**, **j** Corresponding MATLAB stress element blocks; **k** Schematic diagram of smart armor recycling (scale bar, 20 mm); **l** Force distribution MATLAB element blocks shows letter “L” corresponding to piezoelectricity tested by new 3 × 3 smart monitoring armor growing with recycled RS crystal solutions.

Biomimetic 3D-printed-RSC are capable of providing superior protection and remarkable piezoelectric sensing capabilities, making them ideal candidates as a smart armor that provides integrated mechanical protection and electrical sensitivity for athletes. Here, to realize the application of smart monitoring and protection, the biomimetic cuttlefish bone structure consisting of 4 by 4 panels was 3D-printed and then immersed in RS solution for 24 h to fabricate a smart armor. Subsequently, the armor was attached to a football player model for testing (Fig. 5a). In the beginning, we conducted a preliminary piezoelectric test by placing a 10-gram weight vertically. The force magnitude distribution and the piezoelectric output diagram are shown in Supplementary Fig. 26. As shown in Fig. 5b, two weights of 10 g and of 5 g are positioned on top of the two sensing elements at a distance of 5 cm from the surface of the smart armor, respectively. A corresponding voltage is generated by each element and nearby element because of the vibration caused by the impact of the weight (Fig. 5c), demonstrating that the output voltage is proportional to the magnitude of the force exerted on each element of the smart armor. Fig. 5d illustrates the 3D and 2D visualization of the force distribution for each pixel, which is reflected by the output piezoelectricity. Supplementary Fig. 27 shows the output of mechanical signals from 16 elements under the impact of weights, by placing flexible force sensors on the 3D-printed-RSC smart armor. The mechanical signal derived from the output voltage signal (Fig. 5d) is consistent with the measured physical input signal. The high accuracy of the output voltage demonstrates the ability to infer the pressure on each pixel. The use of smart armor allows for accurate positioning of the impact based on the different masses of the object. As shown in Fig. 5e, the force analysis of the pigment block is based on the free fall of 3D-printed letters from the same height (20 cm) perpendicular to the armor surface. According to the MATLAB color block, the letters are S, D, S, and U, with each letter’s shape and alignment being determined, thereby providing the final range of force based on the voltage generated. The results demonstrate that the 3D-printed piezoelectric array can be applied to football games to detect the location and magnitude of the impact force on the players.

In our previous studies, the growth of RS crystals in biomimetic 3D-printed cuttlefish bones has been demonstrated to be healable and recyclable. Therefore, utilizing these crystal properties as advantages, armor that has been damaged results from the impact and breakage can be repurposed by using the healable properties. In the design of our intelligent piezoelectric induction protective armor, we primarily relied on the 4 × 4 arrays of 3D-printed cuttlefish bone structures grown with RS crystals. The crystals of one of the unit blocks in the 4 × 4 armor were broken by an external impact. During the healing process, the RS solution is injected into the damaged smart armor using a needle as depicted in Fig. 5f. To determine the magnitude of the piezoelectric response of the smart armor before and after the repair, MATLAB pigment blocks are applied to visualize the output voltage. 3D-printed L-shaped weights (10 g) were used for free-fall impacts perpendicular to the 4 × 4 detection armor, with the output voltage collected and presented as a MATLAB pigment block. It shows that before the repair, the device cannot work well due to the disconnection of RS crystals, while the output voltage works well after the repair (Fig. 5g, j). Fig. 5h and Fig. 5i depict the top partial view of the damaged sample under an optical microscope. When viewed from the perspective of athletes, the production of recyclable armor contributes to the recycling of raw materials, as well as providing athletes with smart armor of different sizes based on their weight. The repaired samples were again subjected to a recycling experiment illustrated in Fig. 5k. As the first step, we immersed the 4 × 4 arrays of 3D-printed armor filled with RS crystals in distilled water heated to 80°, which caused the crystals to melt completely. After this, the 3 × 3 arrays 3D-printed cuttlebone armor sample was immersed in the RS solution. As soon as the RS solution had soaked into the sample completely, it was removed from the solution and allowed to rest at room temperature for another 24 h. For the purpose of verifying whether the sample after the cycle retains the same piezoelectric properties, the sample was still perpendicular to the free fall of the sample by the 3D-printed L-shaped object (10 g), based on the piezoelectric properties, MATLAB was used to calculate the pigment block utilizing the corresponding output voltage. Based on the output voltage of the recycled 3 × 3 arrays sample, the pigment block letter “L” was successfully sensed and demonstrated (Fig. 5l), showing the excellent sustainable potential of the 3D-printed cuttlebone armor.

### Application of the composites for smart knee pad

**Fig. 6:**
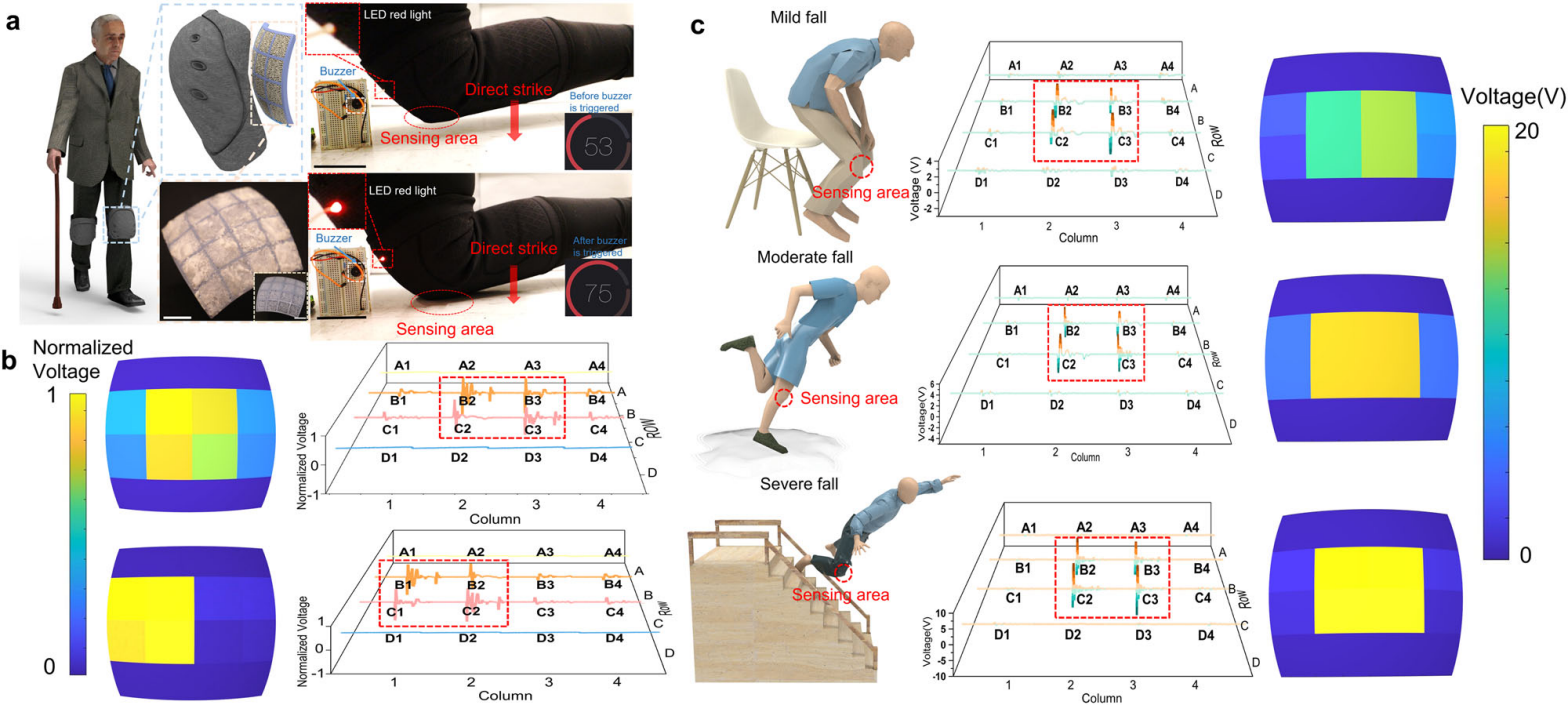
Application of the composites for smart fall detection protection enhancement knee pad. **a** Schematic diagrams and images of knee pad (scale bar, 10 mm), as well as an alarm detection test for knee pad (scale bar, 30 mm); **b** MATLAB element block distribution and the voltage waveforms of output voltage obtained from smart knee protector fall test; **c** Smart knee pad induction the data collection of voltage output waveform and MATLAB element block distribution of piezoelectricity for different levels of falls, including mild fall, moderate fall, and severe fall.

The need for knee pads is crucial among older people in their daily lives. However, most knee pads only provide mechanical protection and hinder the further development of smart protective devices for senior people^40^. In light of this premise, we developed a smart biomimetic knee pad with 3D-printed-RSC that can provide integrated mechanical protection, serve as a fall detection alarm as well as collecting the data for further medical treatment. A curved pad array that matches the shape of the knee was designed and then 3D-printed with cuttlebone-inspired structures. After the RS crystal growth for 24 h, it is placed on the knee for future tests. Each element of the 4 × 4 arrays in the 3D-printed knee pad was connected to the Arduino Uno, buzzer, and led lights to the breadboard (Supplementary Fig. 28). With the knee bent and the induction knee brace bound, we hit the ground from three different angles (vertical center knee impact, inner knee impact, outer knee impact) (Fig. 6a). For each of the three different orientations of impact, the voltage diagrams and the MATLAB pigment block diagrams are illustrated in Fig. 6b and Supplementary Fig. 29. By observing the MATLAB pigment block diagram, it is possible to distinguish the location of the force impact and detect the magnitude of the force during the fall. Providing more effective enhanced knee protection for the elderly is feasible by analyzing different positions of falling.

In addition, the alarm will ring when the falling of the elderly is detected. The alarm threshold value for the smart knee brace is 900 mV, at which point the buzzer sounds and an indicator LED light illuminates (Supplementary Movie. 1) as soon as the voltage generated from the impact of the knee pad on the ground was detected. To further analyze the level of injury, we tested the amplitude of voltage generated by the knee pad by performing different heights of fall. The levels of injury, which included mild, moderate, and severe are related to three different types of falls, respectively. As shown in Fig. 6c, falling from the chair results in a voltage of ~7 V_peak to peak_ generated in the middle part of the curved array. By increasing the falling height, the voltage generated from the pad increases to 11 V_peak to peak_ when falling from standing up. By further increasing the falling height, the voltage increases to 20 V_peak to peak_ when the senior falls from the stairs. Fig. 6c presents the MATLAB pigment blocks and piezoelectric curves that correspond to the different types of fall injuries. As a result, a clear identification can be made to diagnose the changes in voltage levels associated with different degrees and locations of injury for further medical treatment. The smart knee pad provides not only mechanical protection but also serves as a fall alarm and sensor that collects data for the analysis of the location and height of falls for further biomedical monitoring applications.

In summary, we constructed a bionic structure with desirable mechanical properties of cuttlefish bone using biomimetic 3D printing technology and grew eco-friendly piezoelectric RS in this platform to generate a sustainable and repairable additional protective cover with the piezoelectric sensor device. We investigated and determined the mechanical and piezoelectric properties of our 3D-printed biomimetic sensor, thereby allowing it to be used as a piezoelectric device with protective capability. On the basis of this study, enhanced smart armor protection for football players and fall-alert devices with data collection for elderly people are demonstrated as potential applications of smart sensing devices. Sports, medicine, military, and other fields are able to benefit from such repairable and recyclable electronic sensor devices.

## Methods

### DLP 3D printing of structures (cuttlefish bone, Triangular, Cubic and Honeycomb structures)

The size and shape of biomimetic structures need to be precisely controlled in order to achieve their excellent mechanical properties. All the 3D-printed structures were designed by “SolidWorks” and “Fusion 360” software, and models were then sliced as layers by “CHITUBOX” software to generate 2D patterns. To fabricate the biomimetic cuttlebone samples, a commercial Digital Light Processing printer (Phrozon sonic mini 4 K, resolution:35 µm) and industry-standard photocurable resin (Phrozon Auqa-Gray 4 K) were used, and the sliced image patterns were projected on the resin surface through a glass tank and then cured via LED optical system. The first structure layer was cured and attached to the substrate, afterward, the substrate was moved up in one layer thickness for the cure of the second layer and so on. The printed structure layers were attached to the bottom of the lifting substrate. For each layer to be able to be attached to the print platform, 2.5 s of exposure time was set, followed by 35 s of exposure time at the bottom. The curing time of 35 s was set at the bottom to ensure that the resin material can fully adhere to the printing platform and will not fall off, after that the curing time of each layer was set to 2.5 s to ensure the printing accuracy and will not be over-cured. After printing, the samples were washed using a 99% Isopropyl alcohol tank and post-cured in a UV chamber (Anycubic) for 15 min.

### Preparation of RS crystal and piezoelectric sample

Sodium carbonate powder (produced by RUPERT, GIBBON& SPIDER, INC) and Potassium bitartrate powder (produced by PURE SUPPLEMENTS) were used as the raw materials. To prepare the RS solution, 80 g of potassium bitartrate powder were slowly poured into 100 ml of distilled water under 75 °C condition while stirring continuously. About one gram of Sodium carbonate powder was periodically added to the continuously heated solution. Additionally, the RS solution preparation was completed once the Sodium carbonate powder had been added and bubbles were no longer present. In low light, at room temperature, the glass beaker containing the RS solution was left for 24 h, resulting in large, nearly transparent crystals. As a means of adhering RS crystals to the gaps within the biomimetic cuttlebone structures and growing more densely, The RS crystals were dissolved in a glass beaker heated to 100 °C, followed by 15-min evaporation of distilled water vapor. After allowing the solution to stand for 24 h, approximately three to five repetitions of the above steps were performed. The dense distribution of RS crystals was observed. Each crystal particle measured approximately 50 μm in size. To verify the crystallization of RS in a polymer, an X-ray Diffraction experiment was performed on the cross-section of a newly broken RS crystal incorporated polymer illustrated in Supplementary Fig. 30. Preparation of piezoelectric devices is accomplished by involving fully immersing the 3D-printed samples in the solution for one hour, followed by different times (60 min to 48 h) of resting after removing the cuttlebone structures from the crystal solution. A cuttlebone-inspired polymeric structure was first 3D-printed with dimensions of 10 mm × 10 mm × 3 mm. The microstructure of the printed structure was then immersed in melted RS (Sodium Potassium Tartrate Tetrahydrate, NaKC_4_H_4_O_6_·4H_2_O) medium in a glass dish to allow the solution to penetrate into the inner structure. Afterward, the immersed structure was placed in the air to allow for the attachment of RS crystal to the internal printed cuttlebone-inspired structure. As shown in Supplementary Fig. 2, RS crystals are initially attached to the 3D-printed cuttlebone walls. As the exposure time in the air gradually increases, the crystal gradually extends outward along the wall length and thickness, eventually filling the entire space in the 3D-printed cuttlebone structure. As shown in Fig. 1b, more and more RS crystals fill the free space of the structure over hours, and the crystal growing process under each specific time was characterized by an optical microscope, SEM (FEI Quanta 450 FEG Scanning Electron Microscope, USA), and CT scanning (micro-CT instrument, Zeiss/Xradia 410 Versa, Germany). For the first 3 h, a few solid crystals were randomly attached to the printed polymeric wall. After 6 h, the cuttlebone-inspired structure was fully wrapped with RS crystal to become the 3D-printed-RSC. 3D-printed smart armor has dimensions of 5.5 cm, 5.5 cm, and 5 mm in length, width, and height, respectively, and consists of four-by-four sensing pixels, each measuring 1 cm by 1 cm by 3 mm. To perform the healing process, the RS solution is first injected into the damaged area of the 3D-printed armor for a growth of 24 h, and then the sample is allowed to stand for 24 h in order to obtain a restored sample.

### Piezoelectric response test

It is a natural property of Rochelle salt that it exhibits piezoelectricity without the need for poling^33^. With three non-neglectable orthogonal piezoelectric coefficients in RS (d_14_, d_25_, d_36_), piezoelectric responses can be measured in almost all axes. To investigate the piezoelectric response, the composite with 24 h of growth of RS crystal inside the printed cuttlebone-inspired polymeric structure was produced and used, and both sides of the composite were covered by silver electrodes. The copper wires were used as the external electrodes to connect the composites with Analog Discovery 2 (Digilent 410-321, Digilent Co. WA, USA) to measure the generated output voltages induced by the applied external stress. The effective piezoelectric coefficient *d*_33_ was characterized by a *d*_33_ meter (YE2730A, APC International, Ltd., Mackeyville, PA, USA). The vibrometer (Polytec MSA-500 Micro System Analyzer, Germany) was used to obtain displacement and phase data of the samples. And the electrical impedance of the sample was measured by impedance analyzer (Agilent 4294 A, Santa Clara, CA, USA).

### Mechanical test

To measure the mechanical property, the printed samples with pure polymer and fabricated RS crystal composites were measured by a universal testing machine (Instron 34SC-1, MA, USA). All the prepared samples were placed on the platform, and the compressive force was applied vertically to the tested samples. In the static compression test, a compressive velocity of 0.5 mm/s was set with a maximum compression distance of 7 mm. In the three-point bending test, a compressive velocity of 0.5 mm/s and a maximum compression distance of 3 mm were selected and applied to the samples with/without notches. In the single-edge notched bend tests, all the pure structure samples were 3D-printed with a thickness of 5 mm and a notch depth of 0.6 mm, and then structures were filled with RS crystal for growing 0–24 h. All the pictures of the crack from the side view were obtained instantly by SEM. To study the fracture toughness for crack initiation (K_IC_), the following equations were used and calculated^41,42^:

1 $${K}_{{IC}}=\frac{P\times S}{B{W}^{\frac{3}{2}}}\times f\left(\frac{a}{W}\right)$$

2 $$f\left(\frac{a}{W}\right)=\frac{3{\left(\frac{a}{W}\right)}^{\frac{1}{2}}\times \left[1.99-\left(\frac{a}{W}\right)\left(1-\frac{a}{W}\right)\left(2.15-3.93\left(\frac{a}{W}\right)+{\left(\frac{a}{W}\right)}^{2}\right)\right]}{2\left(1+2\frac{a}{W}\right)\times {\left(1-\frac{a}{W}\right)}^{\frac{3}{2}}}$$

whereas *P* is the maximum load during the test, S is the support span (2 cm), a is the notch depth (0.6 mm), W is the sample width (5 mm), B is the thickness of the sample (3 mm). To further calculate and analyze the fracture toughness for crack propagation (K_JC_), the J- integral equation developed by James R. Rice was utilized. The J-integral equation based on the elastic and plastic contribution can be presented as *J*  *= J*_*el*_ *+* *J*_*pl*_. *J*_*el*_ is the elastic component, which can be calculated by the following equation^43,44^:

3 $${J}_{{el}}=\frac{{K}_{{IC}}^{2}}{E{\prime} }$$

4 $$E{\prime}={E(1-v)}^{2}$$

where *E* is Young’s modulus, and *v* is the Poisson’s ratio. Furthermore, *J*_*pl*_, the plastic component, can be calculated as^45^:

5 $${J}_{{pl}}=\frac{2{A}_{{pl}}}{B(W-a)}$$

where *A*_*pl*_ is the plastic area under the load-displacement curve. Thus, the *K*_*JC*_ is demonstrated in the following equation by transforming J-integral equation^46^:

6 $${K}_{{JC}}={({JE}{\prime} )}^{1/2}$$

The crack extension (∆a) is related to the following equations^46^:

7 $${a}_{n}={a}_{n-1}+\frac{(W-{a}_{n-1})}{2}\times \frac{({C}_{n}-{C}_{n-1})}{{C}_{n}}$$

8 $${C}_{n}=\frac{{u}_{n}}{{f}_{n}}$$

9 $$\triangle a={a}_{n}-a$$

In the equation, *a*_*n*_, *C*_*n*_, *u*_*n*_, and *f*_*n*_ represent crack length, complaisance, displacement, and force at each point after the departure of the crack, respectively. A mechanical test is conducted using ten samples under the same conditions to reduce experimental error.

### COMSOL multiphysics simulation for piezoelectric response and mechanical tests

The cuttlebone composite models were designed and optimized in Solidworks software and then imported into COMSOL Multiphysics version 5.6 for simulation of piezoelectric response and mechanical test. In the piezoelectric simulation, mechanical deformation occurred through the whole composite structure, inducing the piezoelectric potential emerging on the top and bottom sides of the composite with electrodes. To demonstrate the potential piezoelectric property of the fabricated composite, a force (10 N) was applied to the model to study the deformation and the piezoelectric output. Moreover, in mechanical test simulation, a compressive force was applied as 1 N to study the crack deflection and deformation of printed raw cuttlebone structure and composite. The piezoelectric coefficient of RS is obtained by COMSOL Multiphysics® software to collect the material references.

### Smart protector devices’ piezoelectric tests

3D-printed smart armor has dimensions of 5.5 cm, 5.5 cm, and 5 mm in length, width, and height, respectively, and consists of four-by-four sensing pixels, each measuring 1 cm by 1 cm by 3 mm. To form positive and negative electrodes, E-solder (VonRoll, USA, Inc) is evenly deposited on the upper and lower surfaces of each sensing element (Fig. 5a). During the experiment and data collection process, we connected each signal acquisition element on the 4 × 4 panel to the Single-board microcontroller (Arduino) by lying down the 3D-printed athletes on a flat table while different weights fell vertically downward from the identical height in order to simulate the impact of soccer balls striking armor during football matches, as well as the impact of athletes’ elbows.

### Fall detection knee pad tests

To better fit the curvature of the knee, the smart knee pad was designed to bend on the x-axis and y-axis simultaneously instead of a flat surface. 3D-printed smart fall detection knee pad measures 5.5 cm, 5.5 cm, and 5 mm in length, width, and height, respectively, with four-by-four sensing pixels measuring 1 cm by 1 cm by 3 mm. Using an Arduino microcontroller, we measured the output voltage for compressing the smart knee pad, as well as connected a red led light and a buzzer for alarm alert. Each sample consists of four 4 × 4 elements connected to a four-channel ADC. The triggered threshold was set at 900 mV (Supplementary Fig. 31).

### Reporting summary

Further information on research design is available in the Nature Portfolio Reporting Summary linked to this article.

### Supplementary information

Supplementary Information

Description of Additional Supplementary Files

Supplementary Movie 1

Reporting Summary

Peer Review

### Source data

Source Data

## Data Availability

The source data generated in this study are provided in the Source Data file. Source data are provided with this paper.

## Code availability

The codes used are open source (COMSOL Multiphysics) and MATLAB codes will be available from the corresponding author upon request.
