# Supplementary material for: Growing recyclable and healable piezoelectric composites in 3D printed bioinspired structure for protective wearable sensor
Source: Nat Commun. 2023 Oct 14;14:6477. doi: 10.1038/s41467-023-41740-6 (PMC10576793; doi:10.1038/s41467-023-41740-6)
Supplement: Supplementary file 1 — Supplementary Information [file 41467_2023_41740_MOESM1_ESM.pdf]

Supplementary Information For

Growing Recyclable and Healable Piezoelectric Composites in 3D Printed Bioinspired Structure for Protective Wearable Sensor

Qingqing He[1, †], Yushun Zeng[2, †], Laiming Jiang[3, †], Ziyu Wang[4, *], Gengxi Lu[2], Haochen Kang[2], Pei Li[4], Brandon Bethers[1], Shengwei Feng[5], Lizhi Sun[5], Peter Sun[6], Chen Gong[2], Jie Jin[7], Yue Hou[4], Runjian Jiang[1], Wenwu Xu[1], Eugene Olevsky[1], Yang Yang[1, *]

[1]Department of Mechanical Engineering, San Diego State University, San Diego, CA 92182, USA.

[2]Alfred E. Mann Department of Biomedical Engineering, Viterbi School of Engineering, University of Southern California, Los Angeles, CA 90089, USA.

[3]College of Materials Science and Engineering, Sichuan University, Chengdu, 610064, China.

[4]The Institute of Technological Sciences, Wuhan University, Wuhan, 430072, China.

[5]Department of Civil and Environmental Engineering, University of California, Irvine, California, CA 92697, USA.

[6]Grossmont College, 8800 Grossmont College Dr, El Cajon, CA 92020, USA.

[7]Canoo Technologies Inc, Torrance, CA 90503, USA.

[*] Correspondence: yyang10@sdsu.edu, zywang@whu.edu.cn

[†] Q. He, Y. Zeng, and L. Jiang contributed equally to this work.

The supplementary information file includes:

Section 1. Supplementary Discussion

Supplementary Figures 1-31
Supplementary Tables 1-2

Section 2. Supplementary References

Section 1. Supplementary Discussion

Supplementary Figures

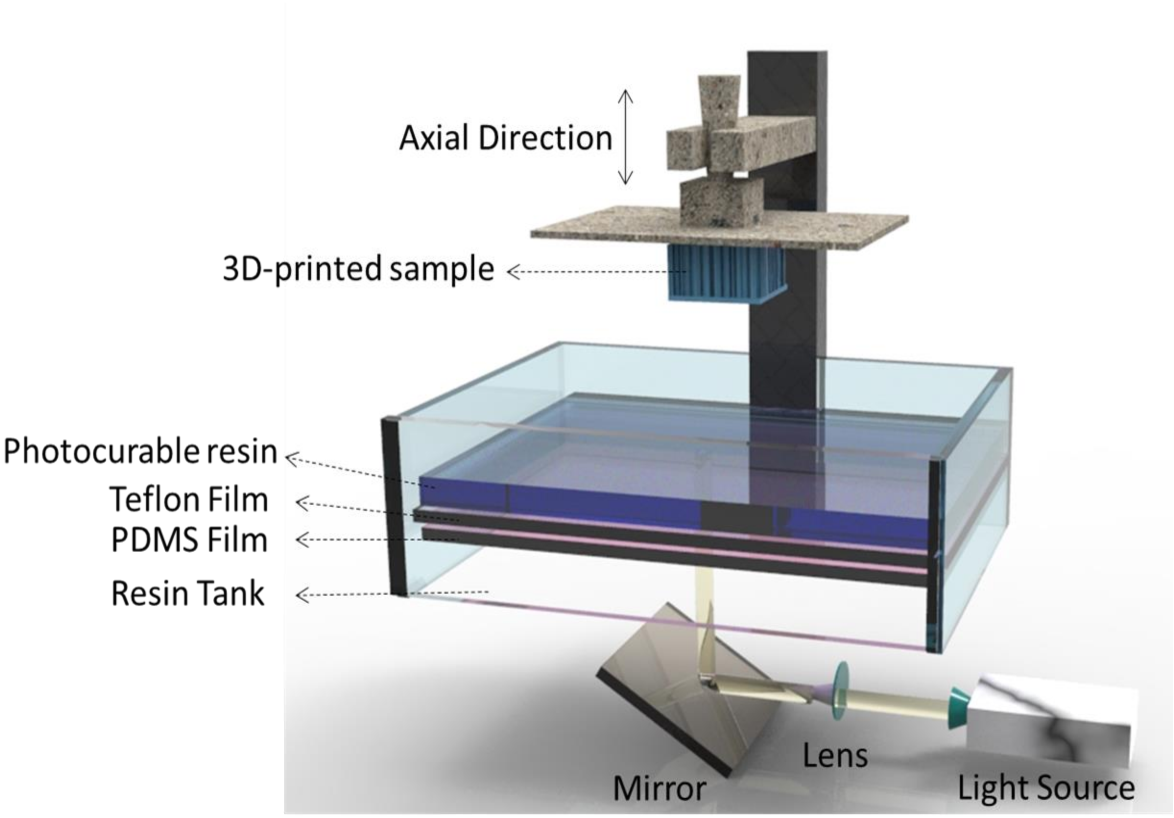

Supplementary Fig.1 The schematic of the DLP-3D printing machine.

As one of the most common resin 3D printing processes, Digital Light Processing (DLP) has gained massive popularity for producing durable, uniform, and leak-proof prototypes and final parts in a wide range of advanced materials characterized by delicate features and smooth surfaces. For our work, we printed the samples using Phrozen sonic mini 4K (PHROZEN TECH CO., LTD.) with 35 μm resolution (Supplementary Fig.1).

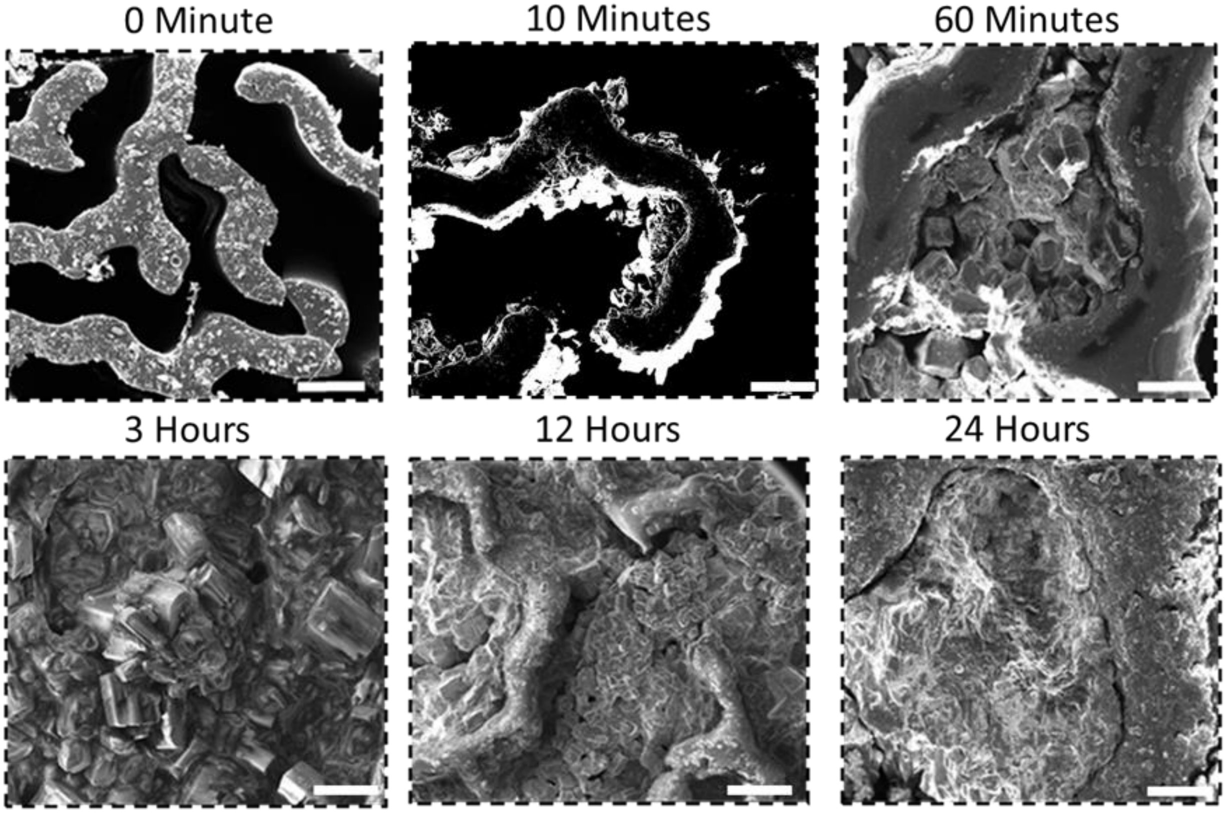

Supplementary Fig.2 Different phases of Rochelle salt crystal growth in the 3D-printed cuttlebone structure as seen by SEM. (scale bar, first row from left to right: 500 μm, 500 μm, 300 μm; second row from left to right: 200 μm, 500 μm, 200 μm)

It is observed that the Rochelle salt (RS) crystals grow along the walls of the 3D-printed cuttlebone sample from the outside inward (Supplementary Fig.2). The sample was not immersed in RS solution for 0 minute as it is the 3D-printed cuttlebone structure with pure polymer. It is noticeable that the RS crystals gradually begin to form along the cuttlebone structure after around 10 minutes, and each individual crystal now has an 8-sided prism shape. Gradual increase in the volume of each RS crystal with increasing time, the cross-sectional length of individual crystals is approximately 50 μm when the sample is immersed in RS solution for 3 hours. After the RS crystals have grown for 12 hours, it can be seen that the crystals are gradually growing densely, and it is gradually impossible to distinguish the form of individual crystals. The RS crystals eventually fill the entire void portion of the structure after 24 hours and are all connected to one another.

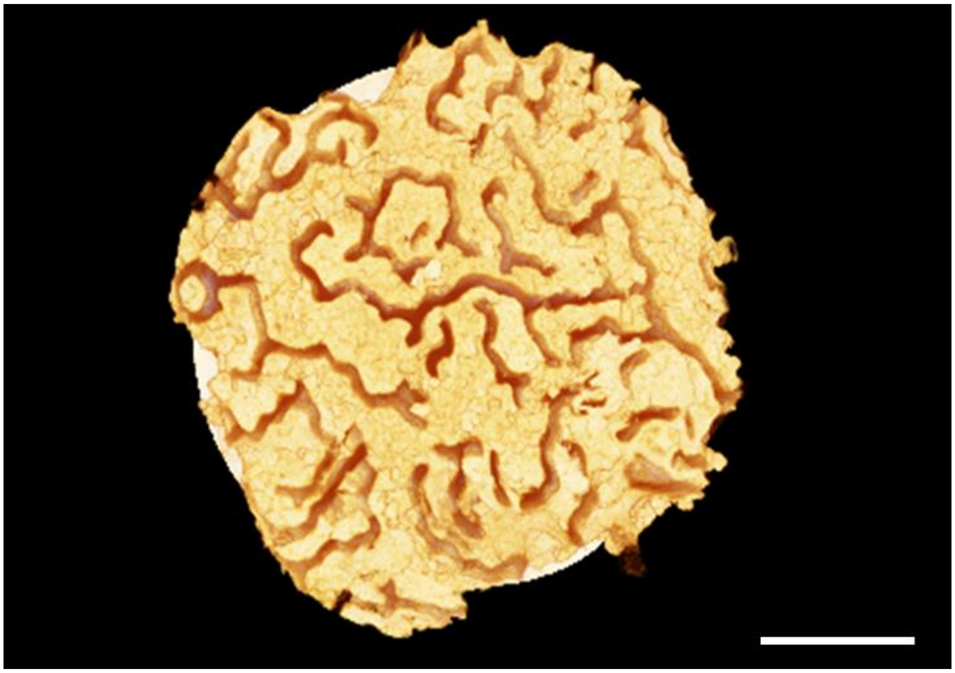

Supplementary Fig.3 The CT scanned photo of 20% cuttlebone polymer structure grown RS crystal for 24 hours. (Scale bar, 5mm)

A computerized tomography (CT) scanning technique is used to visualize the sample in order to determine whether the RS crystals fill the entire sample void space. The pixel size is approximately 12 microns. As shown in Supplementary Fig.3, it is easy to see the shape of the 3D-printed cuttlebone structure, which has been completely filled with RS crystal after 24 hours of growth.

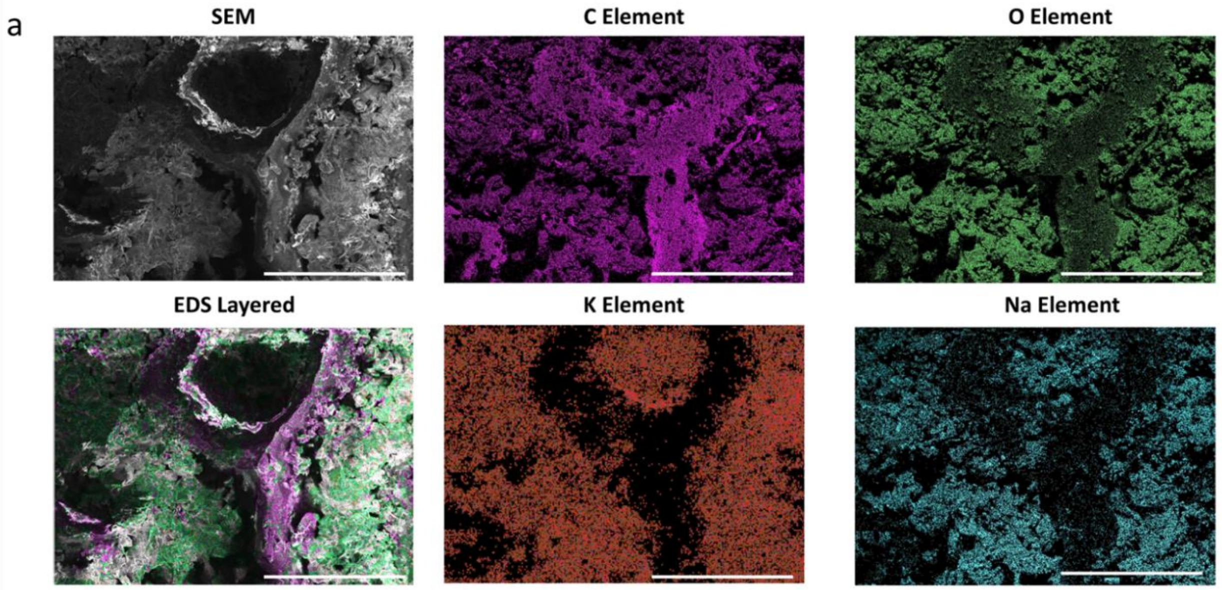

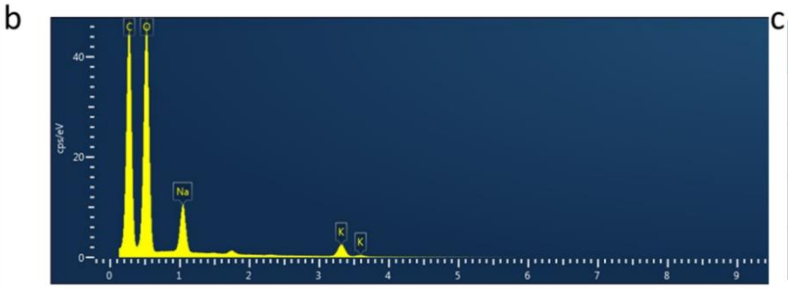

c

Element	Weight%	Atomic%
C	46.01	54.16
O	47.62	42.30
Na	4.67	2.92
K	1.69	0.62

Supplementary Fig.4: SEM-EDS of 3D-Printed-RSC. (a) SEM photo and EDS element layered photos (scale bars: 500μm); (b) EDS spectrum; (c) The weight ratio and atomic ratio of four different elements.

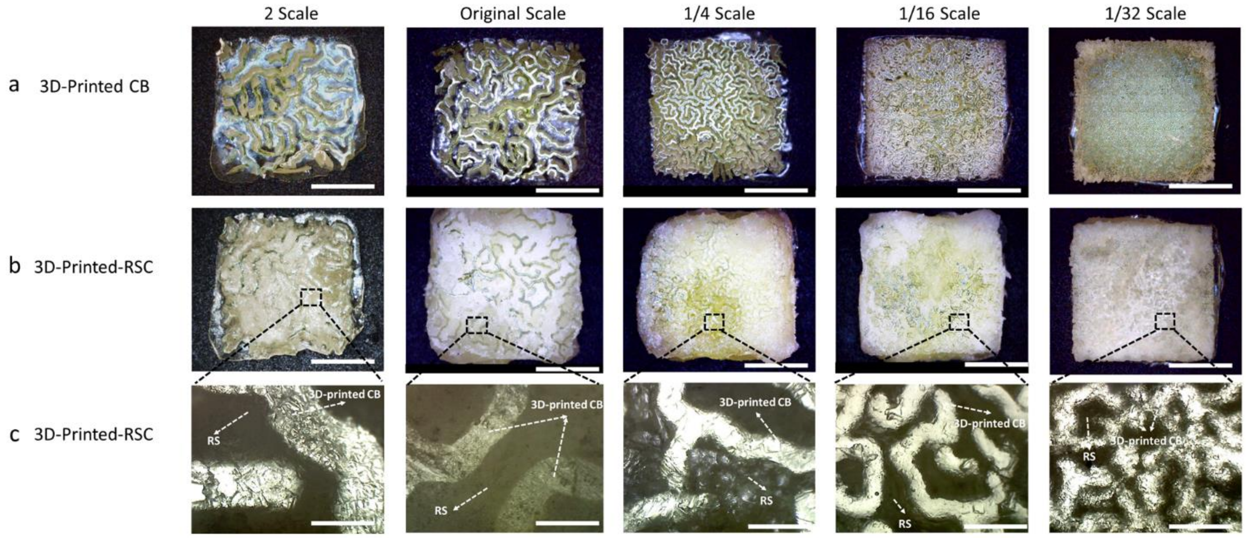

Supplementary Fig.5: 3D-printed-RSC with different scales. (a) 3D-printed CB with the same 20% cuttlebone ratio but different scales. (Scale bars: 5mm); (b) 3D-printed-RSC with the same 20% cuttlebone ratio but different scales. (Scale bars: 5mm); (c) Microscope photos of 3D-printed-RSC with same 20% cuttlebone ratio but different scales. (Scale bars: 500μm).

Our work involved developing 3D-printed-RSC using the Phrozen Sonic MINI 4K 3D printer, which has a resolution of 50 micrometers, and using Aqua-Gray 4K resin. In order to study the size effect, three different sample scales were produced with a Micro-SLA 3D printer. As for the resin, it is UHR Kudo 3D Inc. Micro-SLA 3D printers are capable of producing objects with a resolution of up to 20 micrometers. For electrical and mechanical comparisons, we 3D-printed three different sizes of samples from the Cuttlebone model to mimic the original size of the Cuttlefish bone in nature. As shown in Supplementary Fig.5(a), from left to right, the second photo shows a sample of the same size (original size) as the one printed with the Phrozen 3D printer in the article. On the premise that the volume of the 3D-printed CB is 20% of the 3D-printed RCB total volume, from left to right are Structure of a cuttlefish bone after 2 times equal scale enlargement (2 scale), the original scale, 1/4 scale, 1/16 scale, 1/32 scale. We added the 1/32 scaled down 3D-printed-RSC structure to further mimic the original size of the Cuttlefish bone in nature. All five different scale samples have the same size of 10×10×3mm. As can be seen in the photographs (Supplementary Fig.5(c)) taken under the microscope, the Rochelle salt crystals are dense and uniformly distributed throughout the 3D-printed cuttlebone.

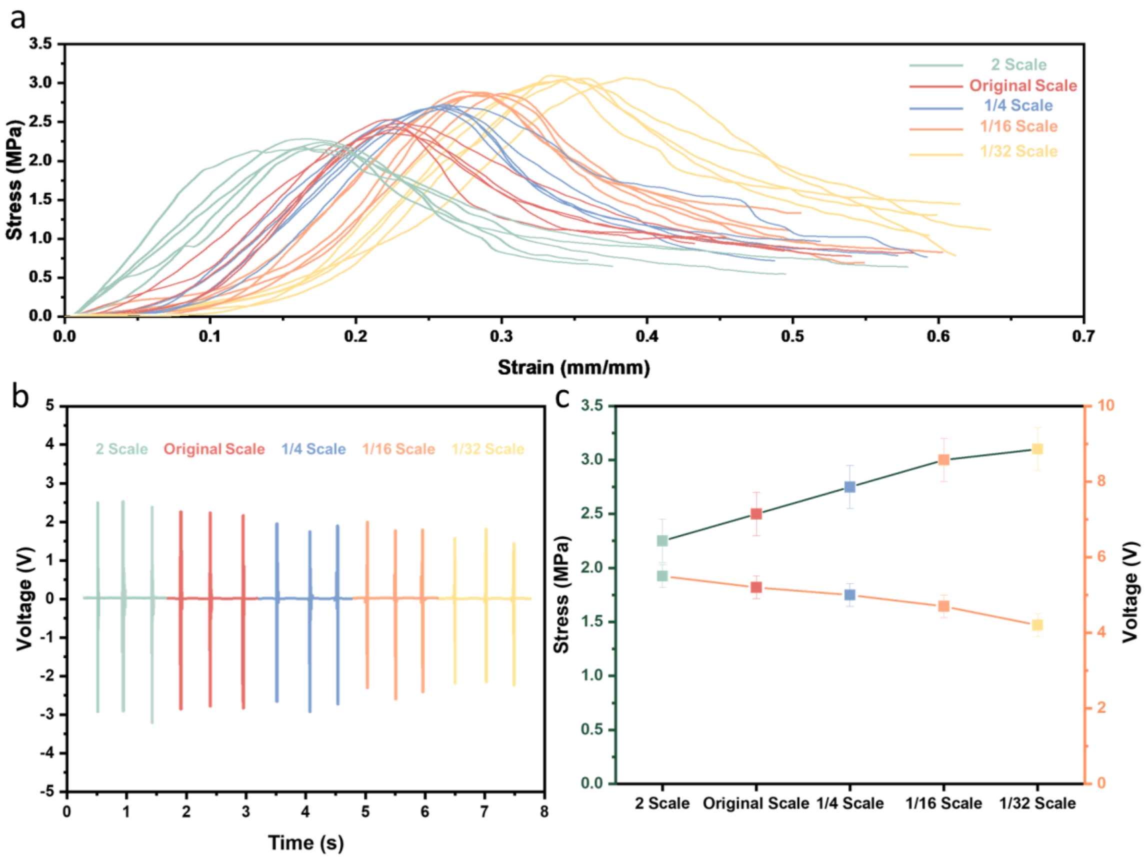

Supplementary Fig.6: The relationship between the structure scale size and performance. (a) Comparison of compression properties of different scales 3D-printed-RSC; (b) Piezoelectric output of different scales 3D-printed-RSC under 2 Hz frequency; (c) The relationship of stress and peak-to-peak output voltage among five different scales 3D-printed-RSC. Error bars represent standard deviation(n=10).

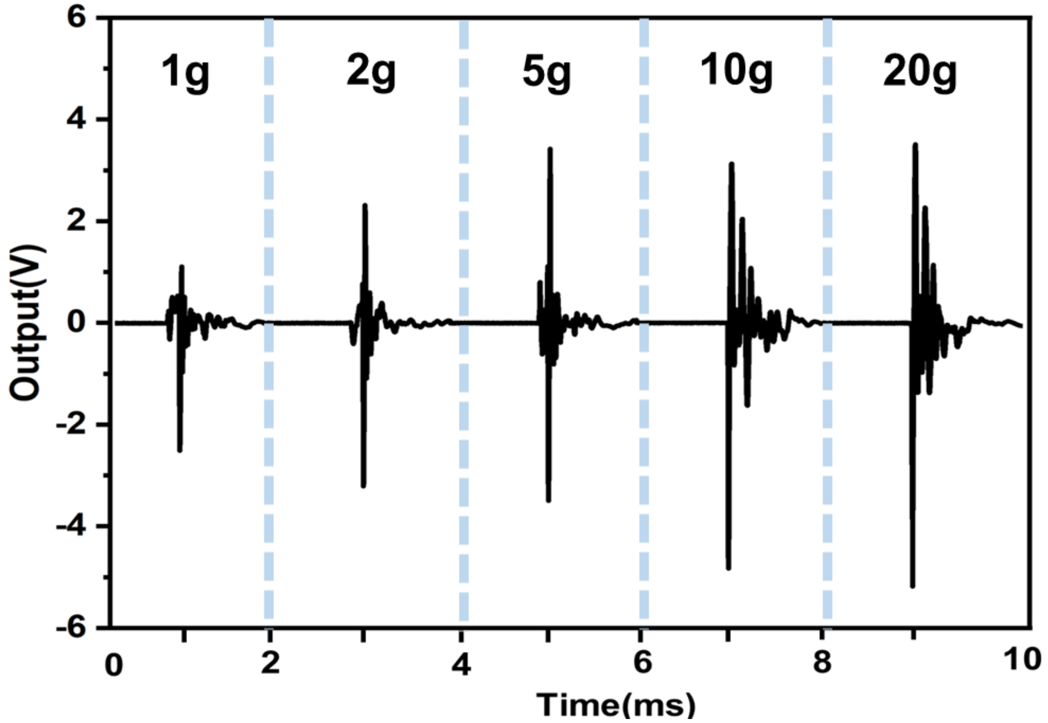

Supplementary Fig.7: Voltage output for piezoelectric testing for 3D-printed-RSC composite with 20% polymer content at the same height using different weights.

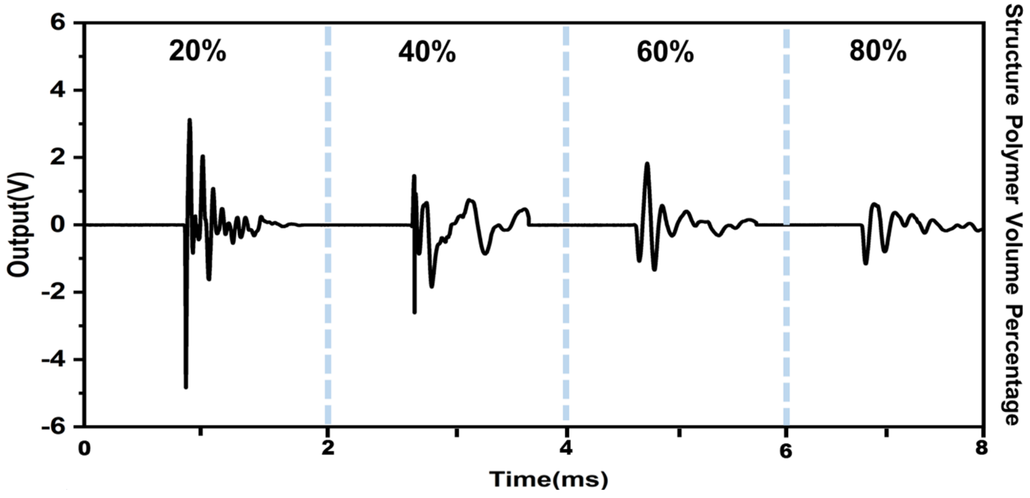

Supplementary Fig.8: Piezoelectric output of different polymer ratios of 3D-printed-RSC for 10 g weight free fall test.

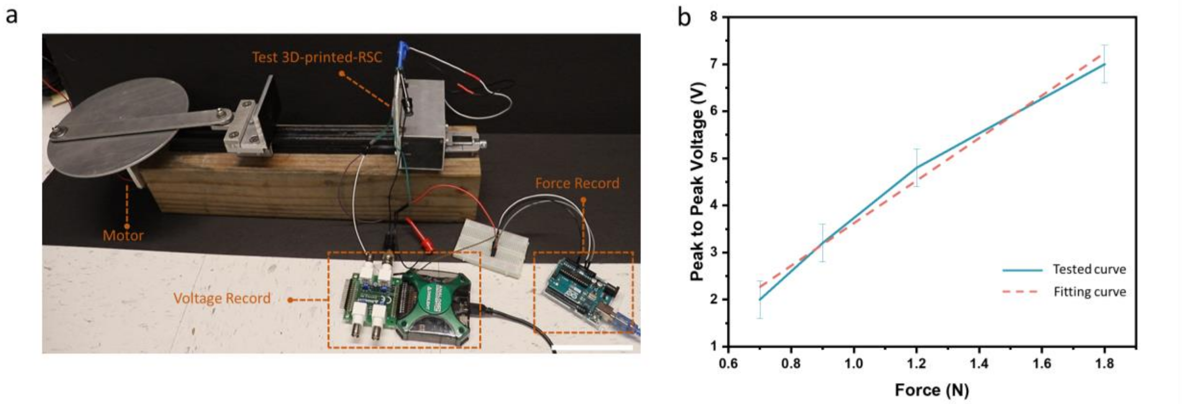

Supplementary Fig.9: Cycle testing performance. (a) Photo of test setup. (Scale bar: 10cm); (b) Peak to peak voltage output at different force. Error bars represent standard deviation(n=10).

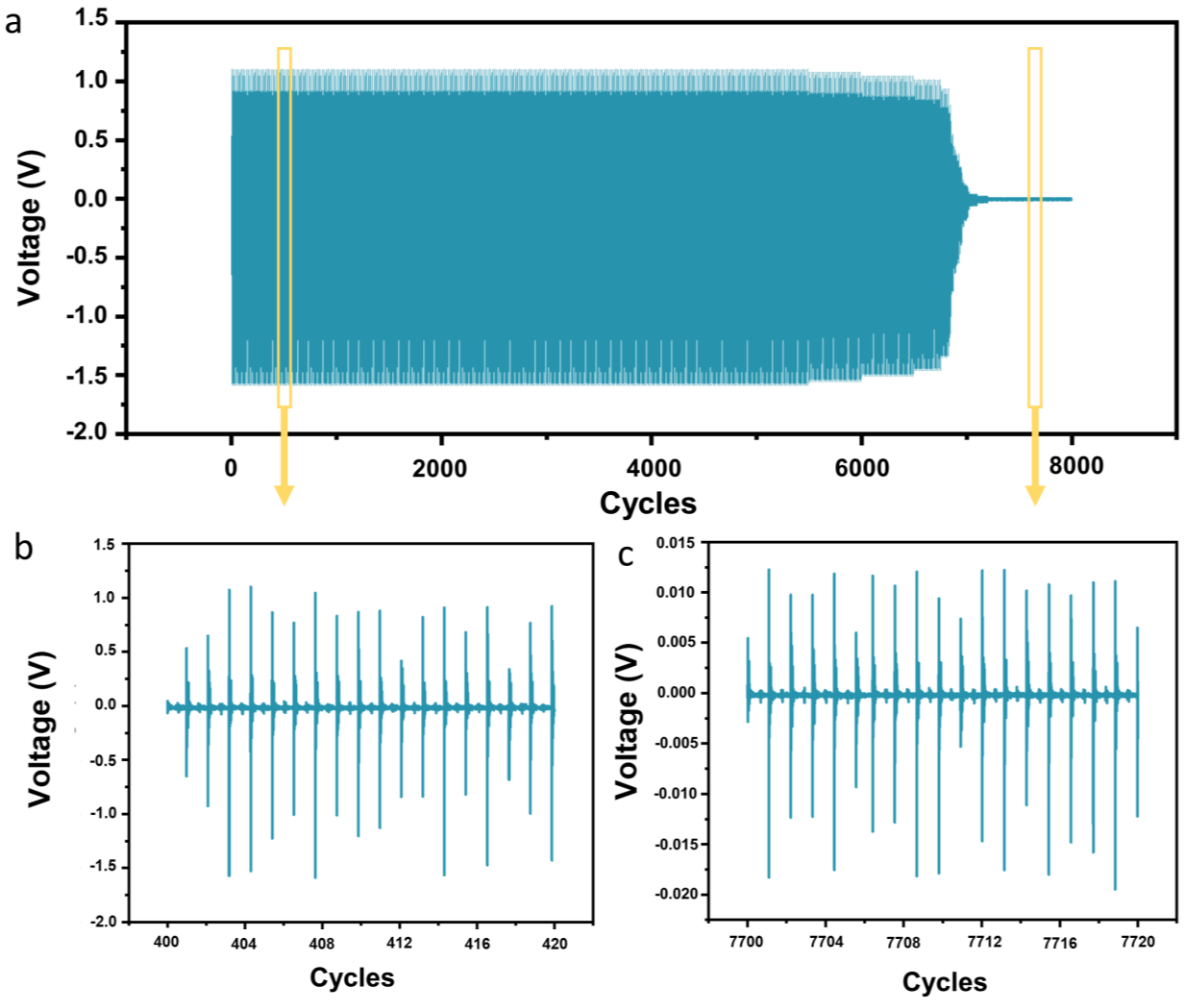

Supplementary Fig.10: 8000 cycles cyclic impact test output voltage graphs

(a) The output voltage over 8000 cycles cyclic impact test under 2Hz frequency; (b) The graph of zoom in between 400 to 420 cycles; (c) The graph of zoom in between 7700 to 7720 cycles.

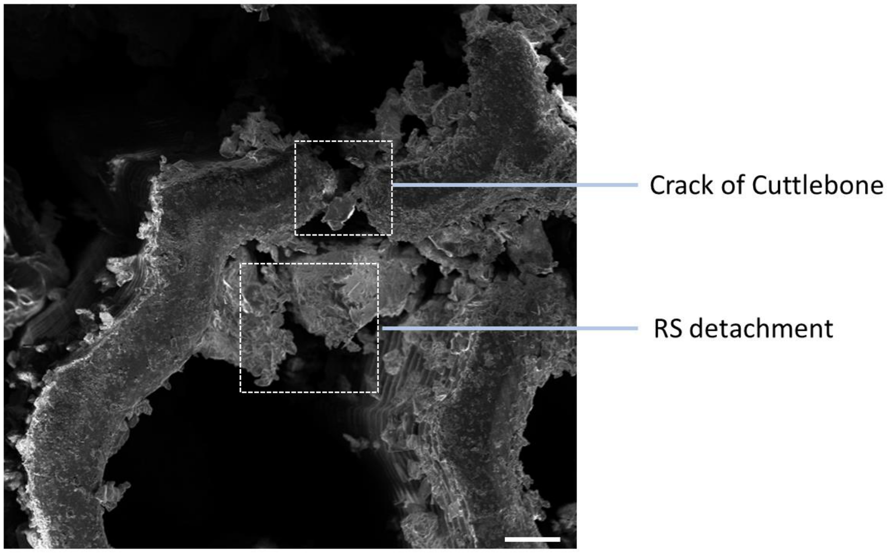

Supplementary Fig.11: SEM photo of 3D-printed-RSC after 8000 cycles cyclic impact test (scale bar: 300 μm).

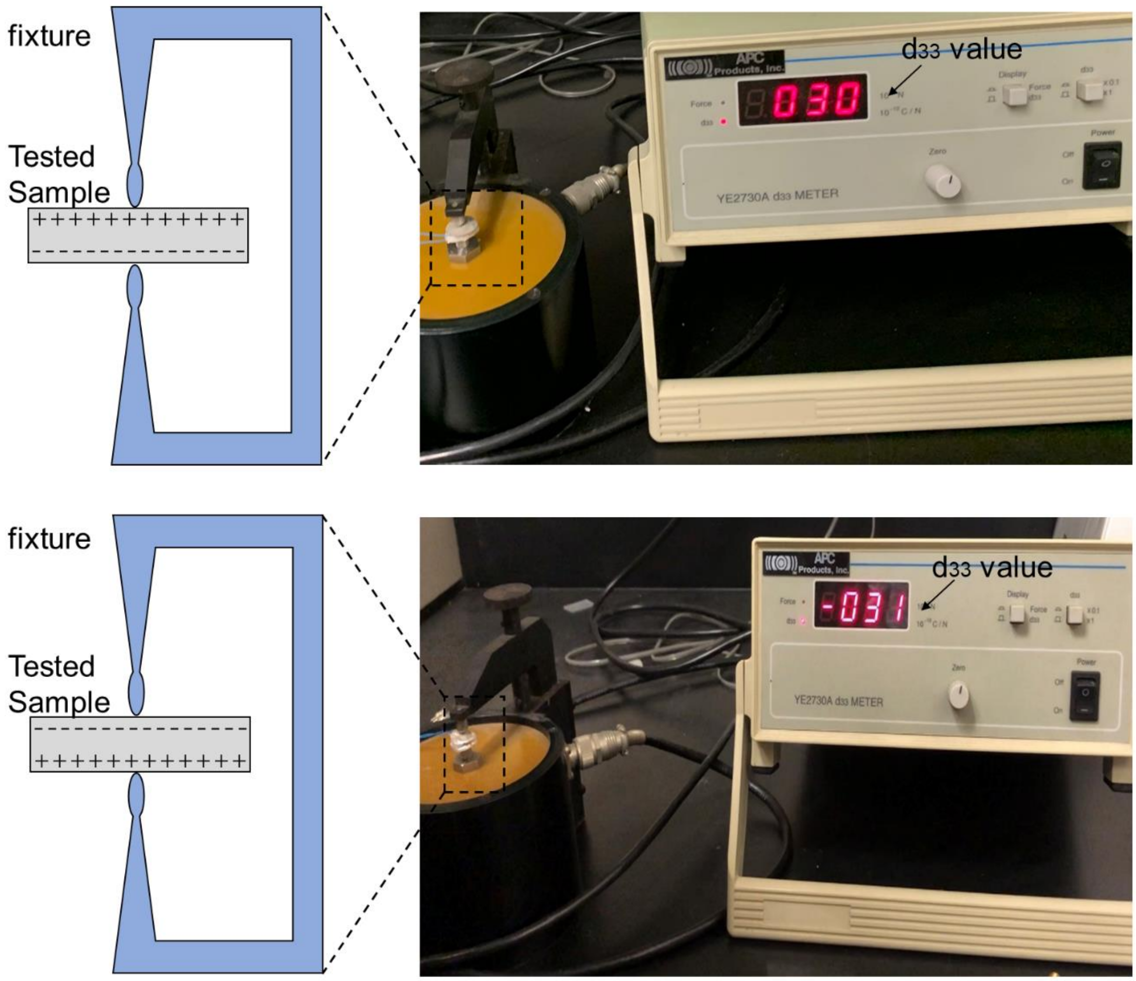

Supplementary Fig.12: Setup of d_{33} measurement and the test results.

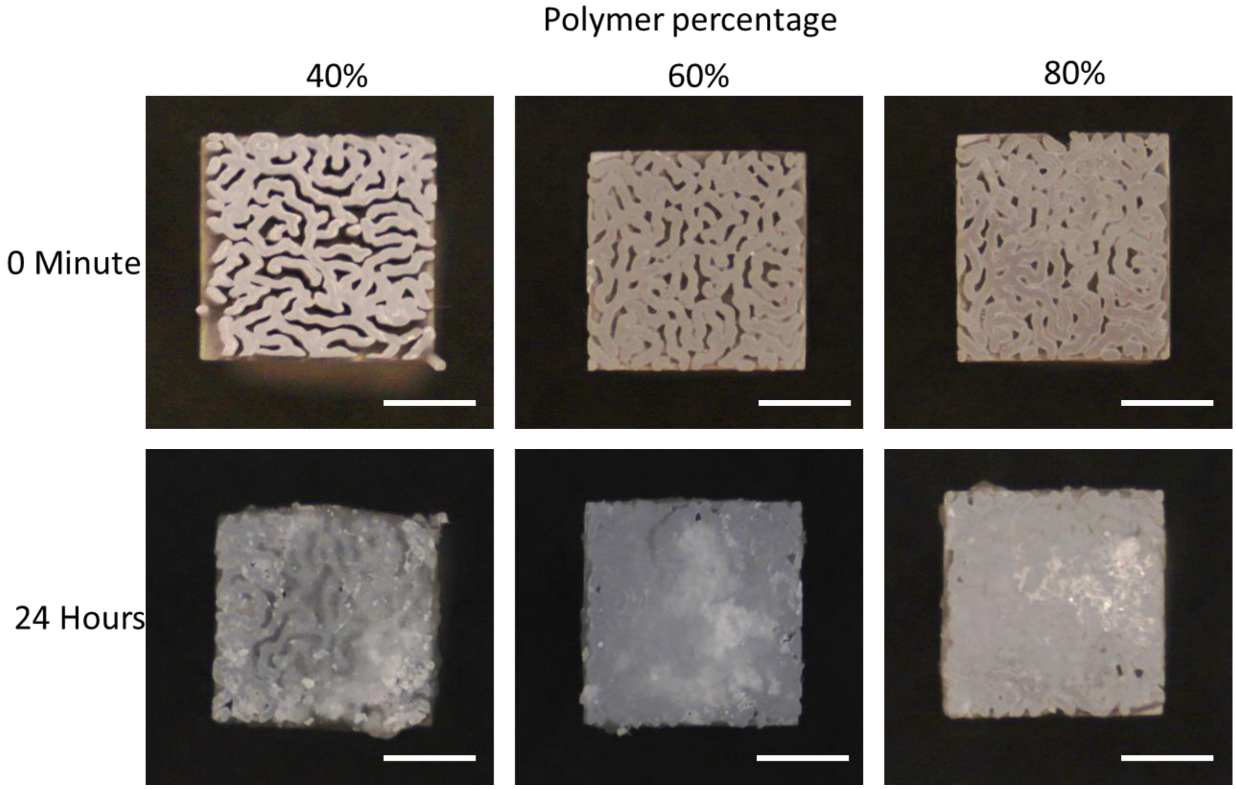

Supplementary Fig.13: Photos of three different volume ratios of 40%, 60%, and 80% of 3D-printed cuttlebone polymer structures without RS crystal, and with grown RS crystals for 24 hours. (Scale bar, 5mm)

For the purpose of comparing the piezoelectric effects of various structural ratios of the same size, three different volume ratios of cuttlebone structures were printed, 40%, 60%, and 80%, respectively. Here, the ratio refers to the percentage of the entire square (10mm x 10mm x 3mm) occupied by the 3D-printed cuttlebone. Following that, three different proportions of 3D-printed samples were immersed in the RS crystal solution for 24 hours simultaneously, after which they were removed for piezoelectric testing. The crystal scale for 3D-printed samples corresponds to 60%, 40%, and 20% shown in the Supplementary Fig.13.

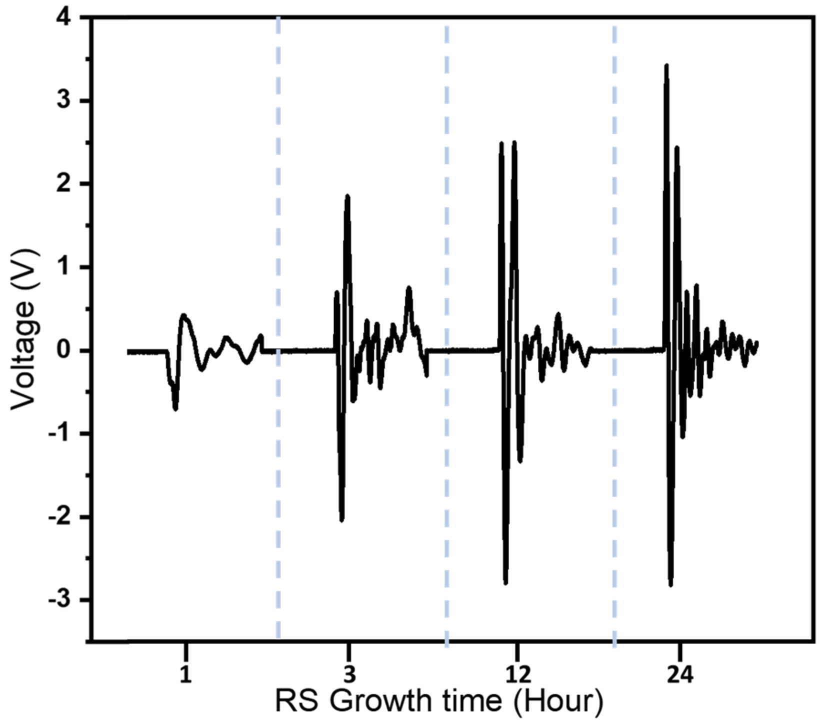

Supplementary Fig.14: The voltage output corresponding to different RS crystal growth times in 20 Vol% polymers.

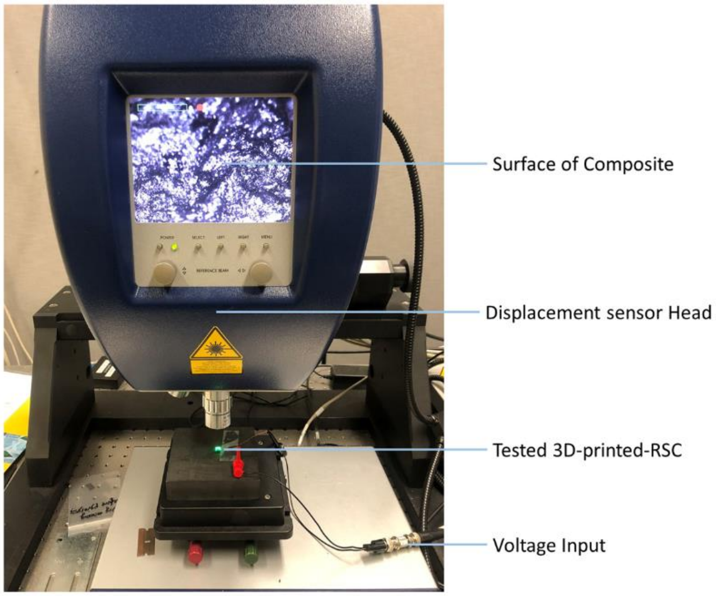

Supplementary Fig.15: MSA-500 laser vibrometer test machine setup.

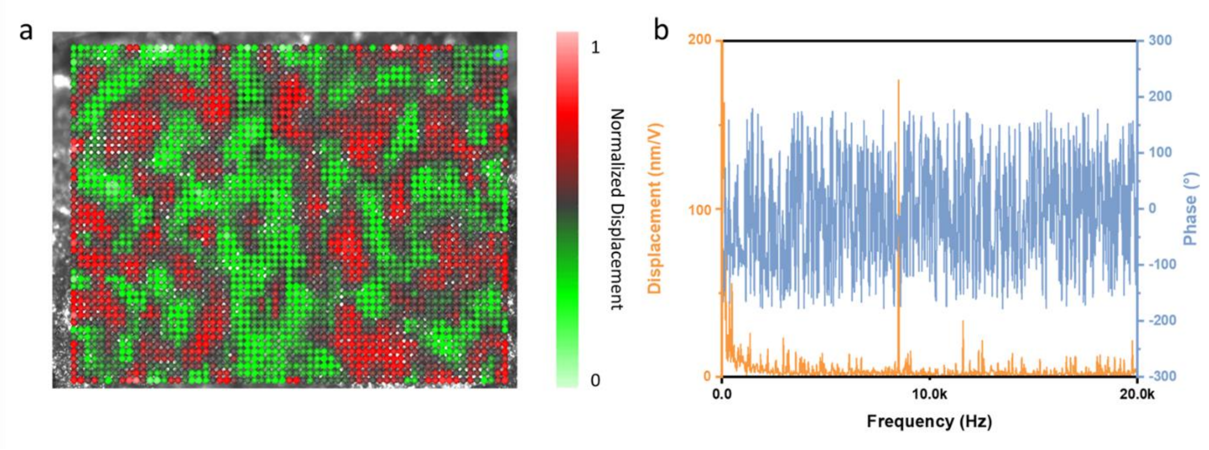

Supplementary Fig.16: Displacement and phase of 3D-printed-RSC measured using a laser vibrometer. (a) Normalized displacement distribution under microscope; (b) Displacement module and phase of 3D-printed-RSC graph.

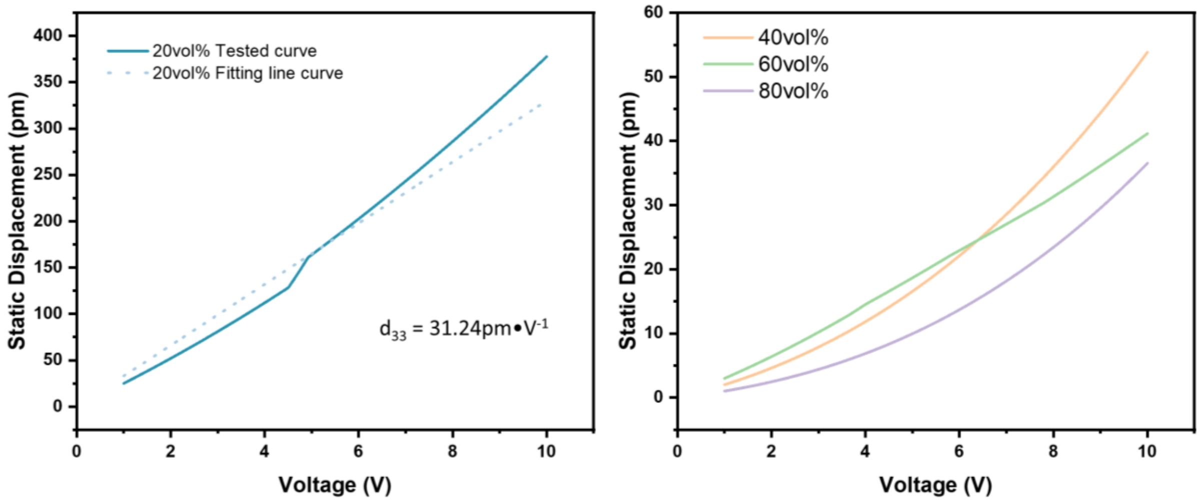

Supplementary Fig.17: Static displacement vs. applied voltage measured on 20%, 40%, 60%, 80% composites.

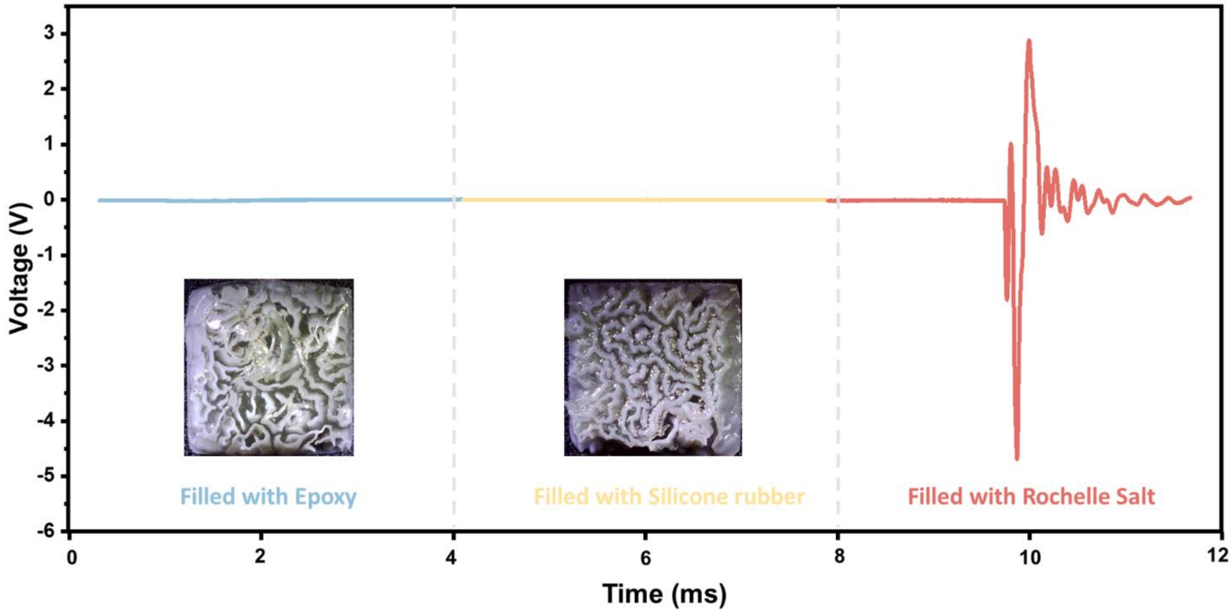

Supplementary Fig.18: Voltage output graphs of 3D-printed-CB filled with different materials.

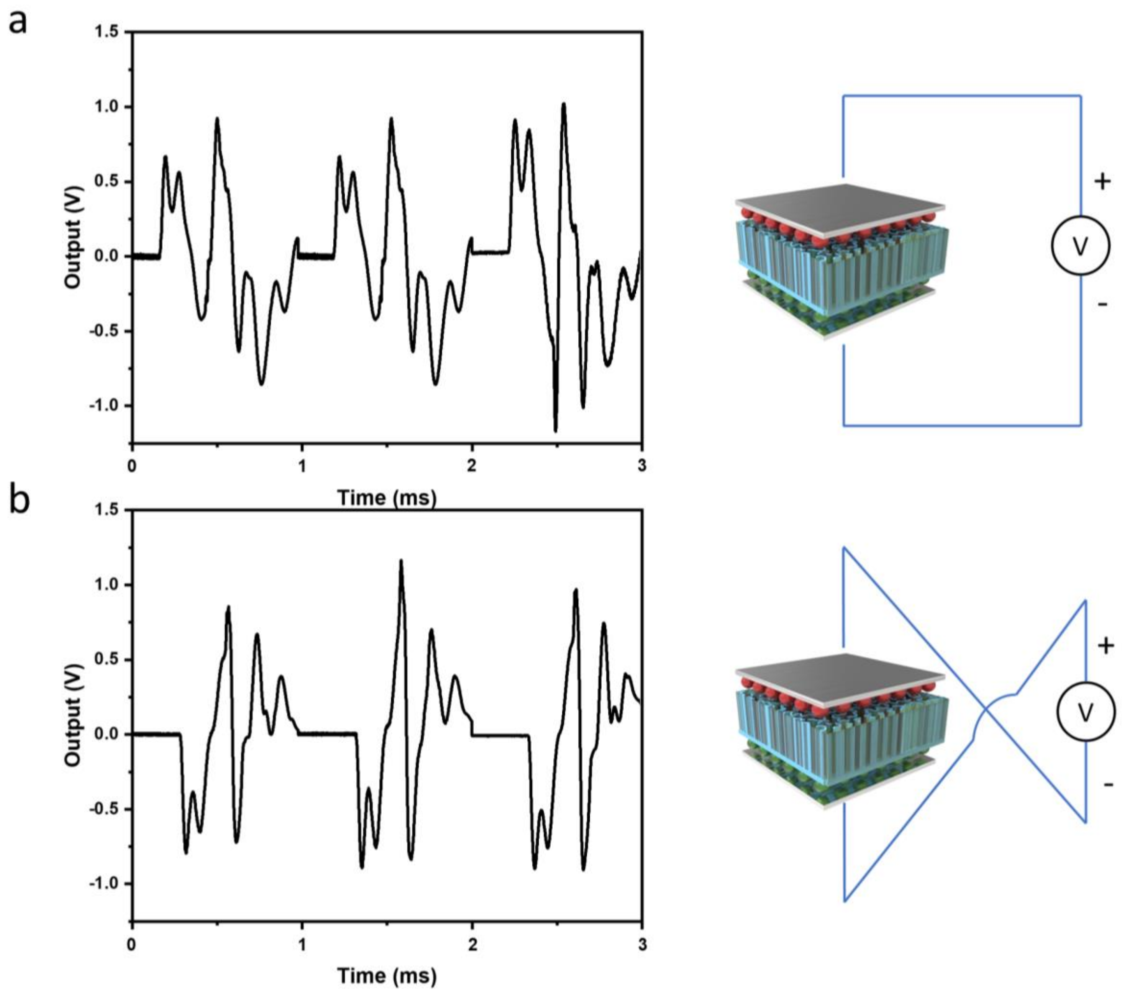

Supplementary Fig.19: (a) The electrical output voltage; (b) The switching polarity tests for output voltage.

$$
\mathrm{d_{KM}} = \begin{bmatrix} dET11 & dET21 & dET31 \\ dET12 & dET22 & dET32 \\ dET13 & dET23 & dET33 \\ dET14 & dET24 & dET34 \\ dET15 & dET25 & dET35 \\ dET16 & dET26 & dET36 \end{bmatrix} = \begin{bmatrix} 0 & 0 & 0 \\ 0 & 0 & 0 \\ 0 & 0 & 0 \\ 3.45e^{-10} & 0 & 0 \\ 0 & 5.4e^{-11} & 0 \\ 0 & 0 & 1.2e^{-11} \end{bmatrix}
$$

Supplementary Fig.20: The piezoelectric coefficients of Rochelle salt.

The piezoelectric coefficients set in the COMSOL simulation are shown in Supplementary Fig. 20. As a linear electromechanical connection between mechanical and electrical conditions, piezoelectricity can be viewed as a form of electricity. Piezoelectric coefficient d is known as the constant for this linear relation. The piezoelectric equations are defined by the following equations:

$$D_k = d_{km} T_m \quad (1)$$

$$S_m = d_{km} E_k \quad (2)$$

The linear electrical behavior of the material is related to D and T, where D is the electric displacement (C/m^2), and T is the electric field component (V/m). Parameters S and T correspond to Hooke's law for elastic materials, S is the strain component, and T is the stress component (N/m^2). It should be noted that k indicates the electric displacement component in a Cartesian reference frame (x_1, x_2, x_3), whereas m =1,...,6 indicates the mechanical stress or strain. According to our study, m =1, 2, and 3 refer to the normal stresses corresponding to x_1, x_2, and x_3 respectively, while m =4, 5, and 6 represent the shear stresses S_{23}, S_{13}, and S_{12}.

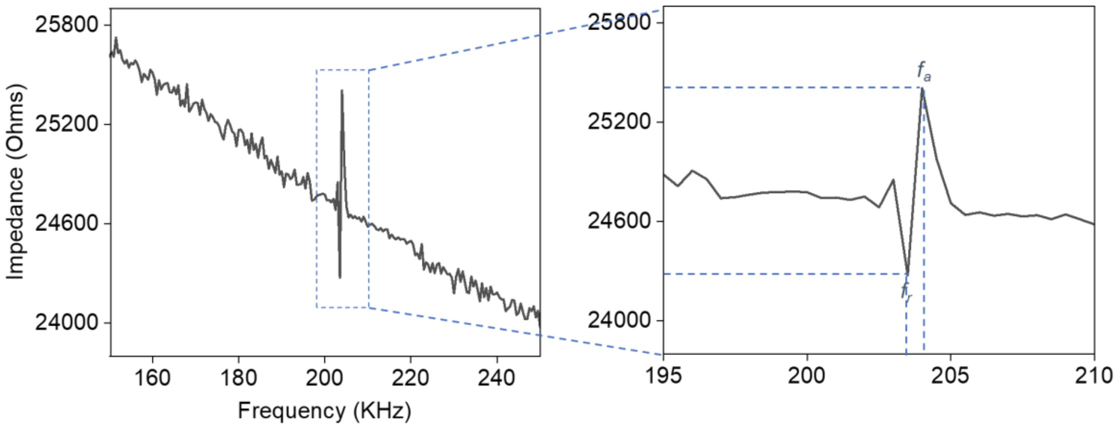

Supplementary Fig.21: Measured Impedance spectrum of the Rochelle salt composite in 20% volume ratio

The electrical impedance of the composite has been measured by impedance analyzer (Agilent 4294A, Santa Clara, CA, USA). The electromechanical coupling coefficient (k_t) of the piezoelectric material can be defined as the following equation, whereas f_r is resonant frequency, and f_a is anti-resonant frequency.

$$k_t = \sqrt{\frac{\pi fr}{2fa} \times \cot \frac{\pi fr}{2fa}} \tag{3}$$

Based on the tested result in below Supplementary Fig. 21, the f_r and f_a can be located as 203.3 kHz, and 204.2 kHz, respectively. The coupling factor has been determined as 9.3% based on the previous response. The central frequency of the composite is located between f_r and f_a, thus, the impedance of the composite is between 24300 ohms and 25400 ohms.

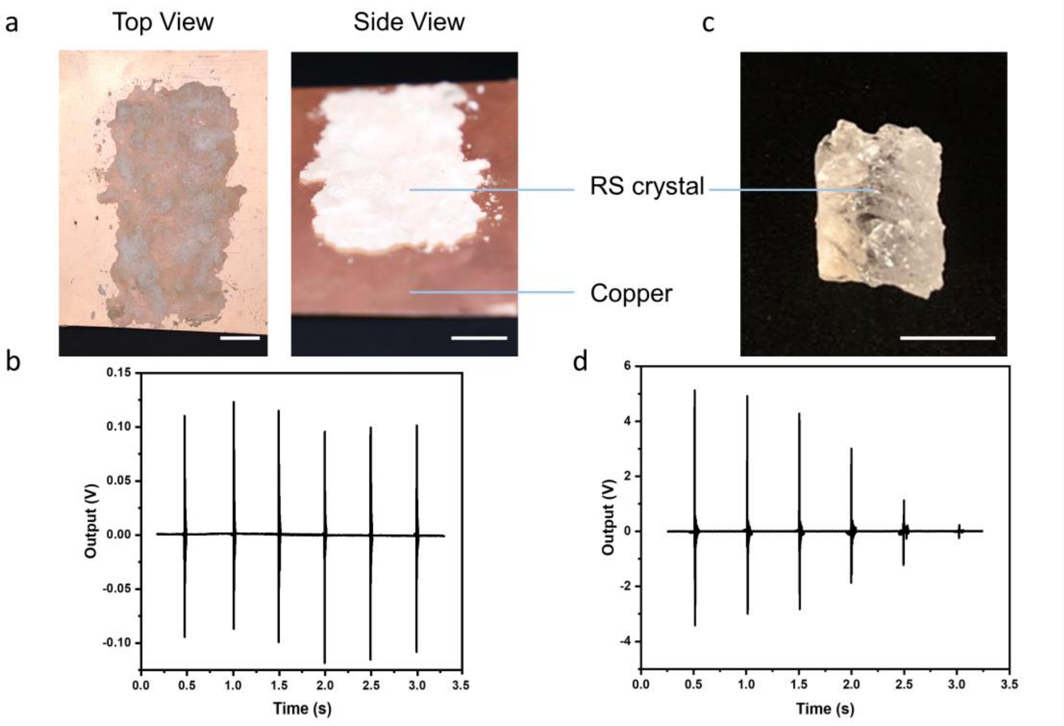

Supplementary Fig.22: Properties of RS piezo film and RS piezo crystal. (a) Photos of RS piezo film (scale bars: 1cm); (b) Output voltage under 2Hz frequency of RS piezo film; (c) Photos of RS piezo crystal (scale bar: 1cm); (d) Output voltage under 2Hz frequency of RS piezo crystal.

An RS piezoelectric film is formed by dropping the RS solution onto the copper sheet, with the copper sheet acting as both an electrode and the growing substrate. E-solder was attached to the RS surface after 24 hours of growth as the other electrode (Supplementary Fig.22a). Supplementary Fig.22b demonstrates the output voltage of an RS piezoelectric film subjected to a cyclic impact test at a frequency of 2 Hz. Moreover, we compared RS crystals for piezoelectric testing (Supplementary Fig.22c). We found that the pure RS crystal is more fragile after five cycles of impact testing due to the lack of a 3D-printed cuttlebone as a mechanical support (Supplementary Fig.22d).

As reported in this paper, manufactured biodegradable 3D-printed-RSC samples exhibit good piezoelectric properties without the need for any poling step. It is a natural property of Rochelle salt that exhibits piezoelectricity without the need for poling. With three non-neglectable orthogonal piezoelectric coefficients in RS (d_{14}, d_{25}, d_{36}), piezoelectric responses can be measured in almost all axes except optical axis (the longest tubular direction of crystals) [1]. Levitskii et al.[2] illustrated RS crystals will decompose at approximately 55 °C, and the polar ferroelectric phase, occurring at the Curie temperature range between −18°C and +24°C, is monoclinic. Our experiments were conducted in a room temperature environment (20°C).

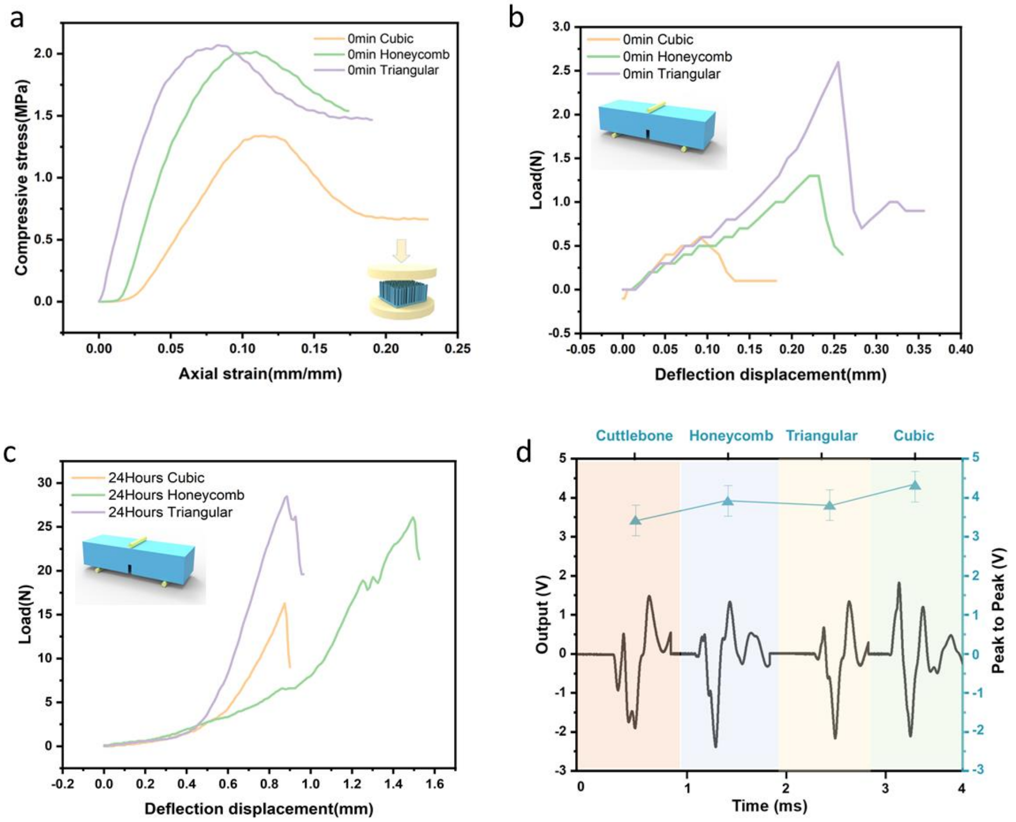

Supplementary Fig.23: Mechanical properties and piezoelectrical output of different 3D-printed structures. (a) Comparison of the load versus displacement among Cubic, Honeycomb, and Triangular structures without RS crystal inside; Compression force versus resistance change for Cubic, Honeycomb, and Triangular structures without RS crystal inside (b), as well as RS crystal inside the structures, shown in (c). (d) Output voltage of Cuttlebone, Honeycomb, Triangular, and Cubic structures. Error bars represent standard deviation(n=10).

To measure the toughness of the material, the standard three-point bending test with a notch was conducted in three different comparison structures, including Cubic, Honeycomb, and Triangular structures (Supplementary Fig.23). Comparative samples were fabricated to the identical size as the 3D-printed cuttlebone structures, in which length, width, and height are 10mm, 5mm, and 3mm respectively, with a notch depth of 0.6mm. Besides, under the impact of 2g weight, the piezoelectric output of the 3D-printed-RSC sample does not significantly differ from that of the other three composites (~20% lower). The 3D-printed-RSC applications in this study, instead, focus on protective piezoelectric sensors, which can be used as a smart armor to normalize the output voltage in order to obtain the corresponding force in equal proportions throughout the smart armor. Considering the protective property and piezoelectrical sensing abilities, we determine that the mechanical performance is more significant, and the piezoelectric performance is applicable to the sensing function, leading us to choose the 3D-printed-RSC as the best solution.

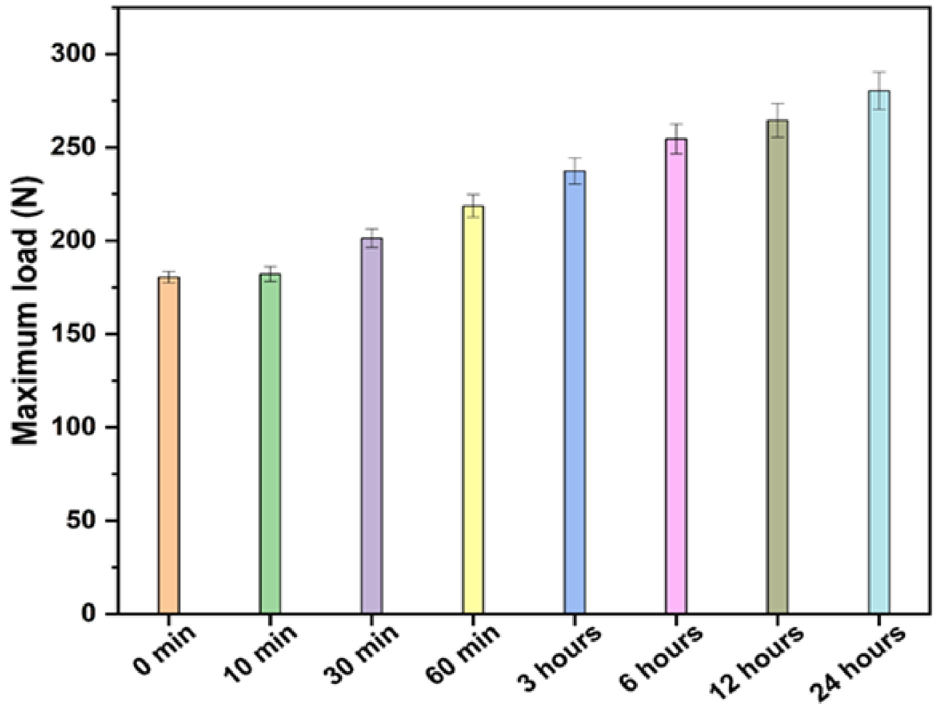

Supplementary Fig.24: Maximum load corresponding to different RS crystal growth rates in 3D-printed cuttlebone samples. Error bars represent standard deviation(n=10).

As the crystal growth time increases, the maximum load (load at failure) increases for composites (Supplementary Fig.24). Consequently, it appears that the Rochelle salt crystal in the 3D-printed cuttlebone structure is a crucial component in crack deflection and energy dissipation. After 24 hours of RS crystal growth, and according to Fig 3d, e, the maximum load gradually stabilizes. RS crystals grown at 24 hours increase the maximum load by nearly 30% over a 3D-printed polymer cuttlebone structure without RS crystals. In this regard, the RS crystals are also expected to have satisfactory mechanical properties and are likely to contribute substantial mechanical protection to the 3D-printed structure.

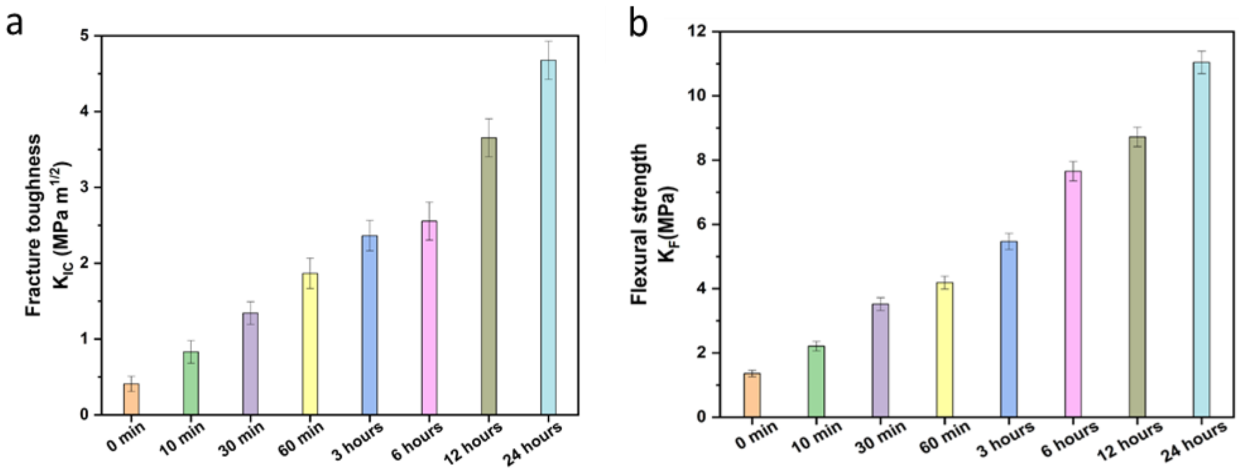

Supplementary Fig.25: Comparison of (a) fracture toughness for crack initiation (K_{IC}) and (b) Flexural strength (K_F) of the 3D-printed cuttlebone-RS composite. Error bars represent standard deviation(n=10).

Fracture toughness refers to a material's ability to resist fracture with originating cracks and plays an important role in the structural integrity and reliability of a structure. It has been demonstrated that the fracture toughness for crack initiation K_{IC} (described as the resistance to crack initiation) for 24 hours RS crystal growth time is significantly higher than for other growth times under the same loading conditions. As crystallization progresses along the wall of the 3D-printed cuttlebone structure, it reaches its maturity after 24 hours of growth. According to the results, the K_{IC} of 24 hours 3D-printed RS has the highest value (4.67 MPa m$^{1/2}$) and has increased by 1044.6% over pure polymer (Supplementary Fig.25).

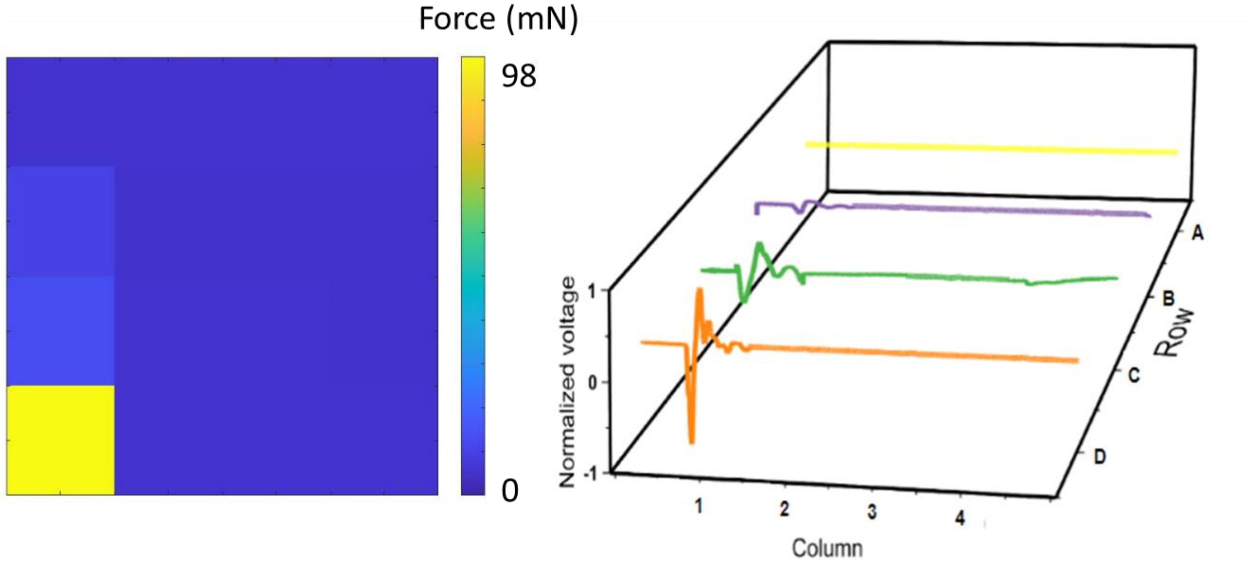

Supplementary Fig.26: Left: Force magnitude distribution obtained from electrodes attached to the sensing armor; Right: Piezoelectric output diagram corresponding to the sensing element.

MATLAB was used to analyze the magnitude distribution of force after uploading the piezoelectric output corresponding to each sensing element. The force magnitude distribution is shown in Supplementary Fig.26, where yellow represents the element with the greatest force. Piezoelectric induction armor with enhanced mechanical protection features 4 rows by 4 columns for a total of 16 sensing units. Supplementary Fig.26 illustrates the piezoelectric output of each inductive element. In accordance with the magnitude distribution of the force, the higher the output piezoelectricity, the greater the force on the detection element.

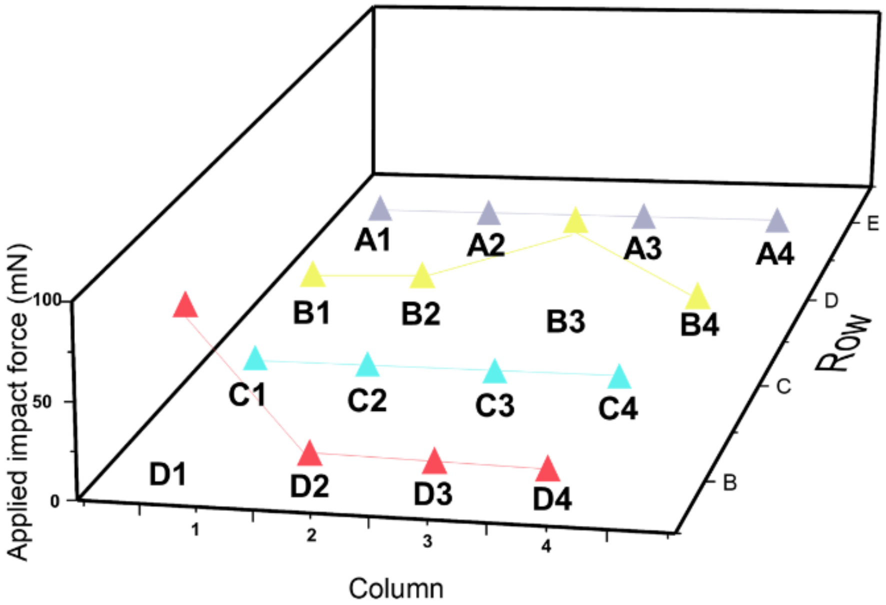

Supplementary Fig.27: Applied impact force of 16 elements of smart armor.

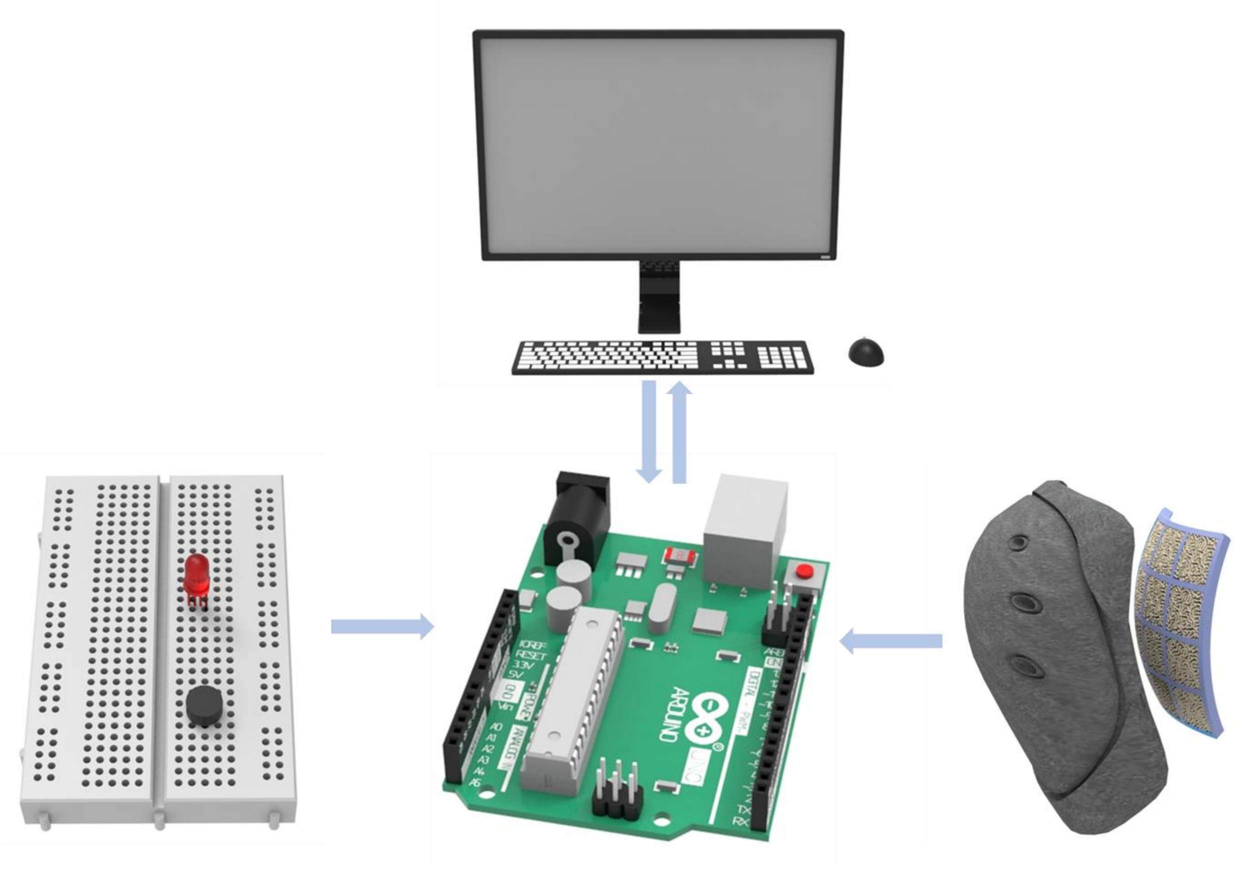

Supplementary Fig.28: Flow diagram for intelligent conductive protection devices test.

The sample tested was a 4x4 original array of intelligent sensor alarm protection knee pads. First of all, In Supplementary Fig.28, a total of 16 inductive components are plated with silver epoxy positive and negative electrodes, and the 16 inductive components are connected to the Arduino Uno development board through the wire, due to multiple lines, the breadboard functions as a collection of circuit boards for connecting the 16 inductive components on the Arduino Uno development board. Additionally, another breadboard connected to the Arduino Uno is used to install the buzzer alarm and LED lights, so that upon impact with the fall detection knee pads, both the buzzer alarm and LED lights will illuminate. A USB port is used to connect the Arduino Uno power supply to the computer, which supplies 3.3 Volts to the circuit.

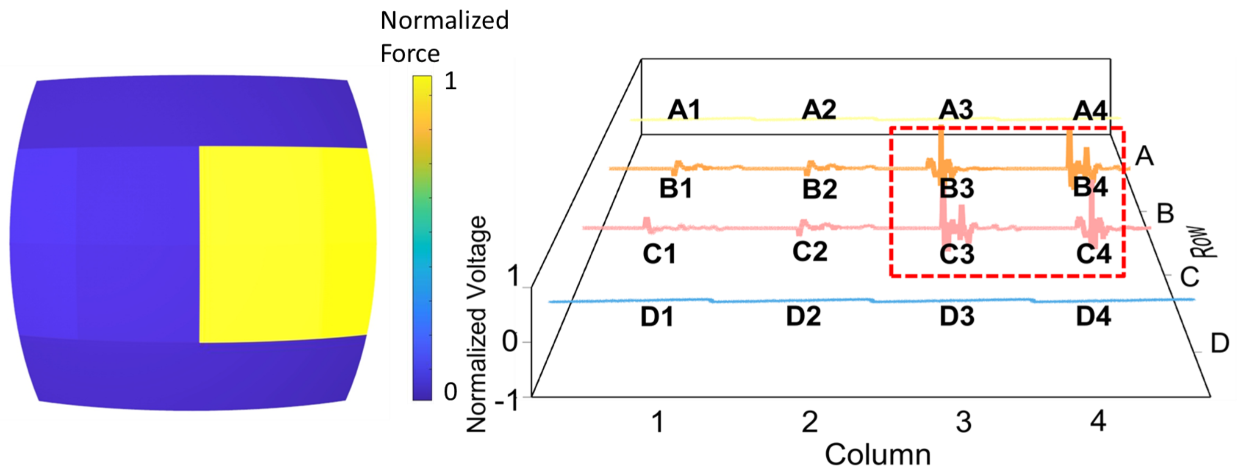

Supplementary Fig.29: Knee anti-fall sensing device right side force test MATLAB pixel block and output voltage diagram.

The inside knee (right side) fall test was verified after the middle knee slip test and the left knee fall assessment. The Supplementary Fig.29 shows a 4 by 4 pigmented block calculated by MATLAB based on the voltage generated by the 3D-printed sensing device due to the impact. The four pigment blocks on the right side of the knee are clearly more stressed than their counterparts on the left side. For a more intuitive understanding of the scale of the voltage generated by the device's piezoelectric effect, the output voltages for each pigment block are also determined, as shown in the Supplementary Fig.29 after normalizing the output voltage, the right side of the four individual units generated by the higher voltage can also be derived, based on the fact that the right side of the knee has a higher force than the other side of the knee.

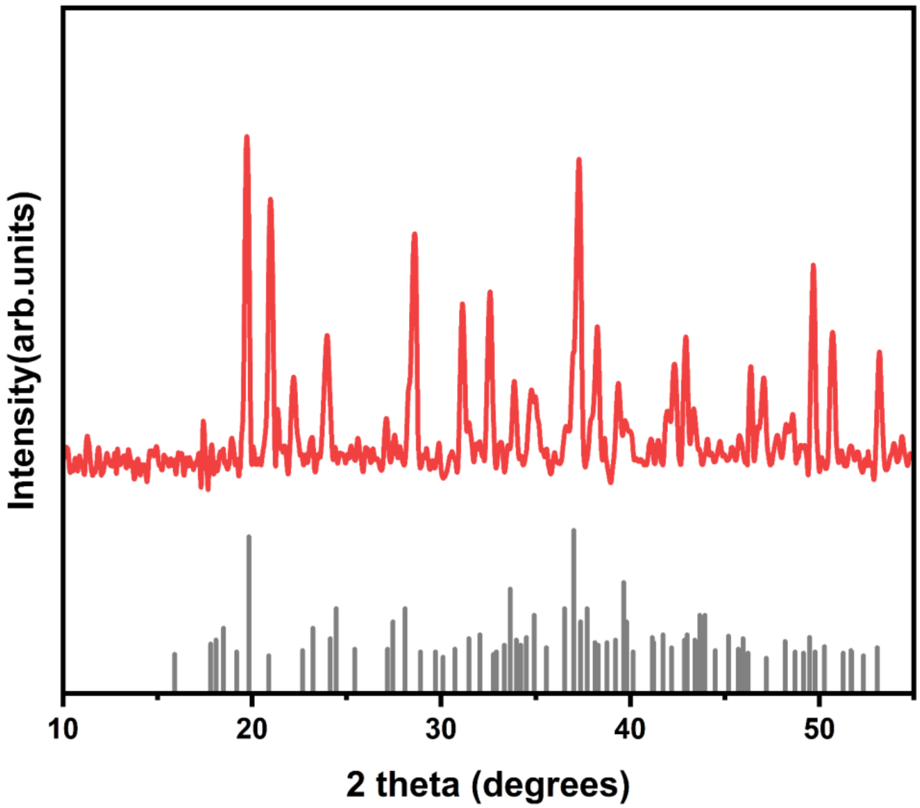

Supplementary Fig.30: Comparison of X-ray diffraction patterns of Rochelle salt crystals grown in the polymer (red line) with the Powder Diffraction File (grey line).

As a microstructural analysis method, X-ray diffraction analysis (XRD) is used to determine the crystallinity of polymers, identify crystalline phases (polymorphism), and establish the orientation of polymers. An X-ray Diffraction experiment was conducted on the cross-section of a freshly broken RS crystal incorporated polymer to verify RS's crystallization in the polymer (Supplementary Fig.30), in which the experimental test results (red lines) were compared with the standard x-ray diffraction powder patterns (grey lines). It has been found that some of the patterns on our tested diffractogram are very similar to those on the standard XRD diffractogram obtained from the Inorganic Crystal Structure Database (ICSD). Diffraction patterns were observed at 18.89°, 27.92°, and 36.69° on our diffractogram, corresponding to planes (021), (231), and (302).

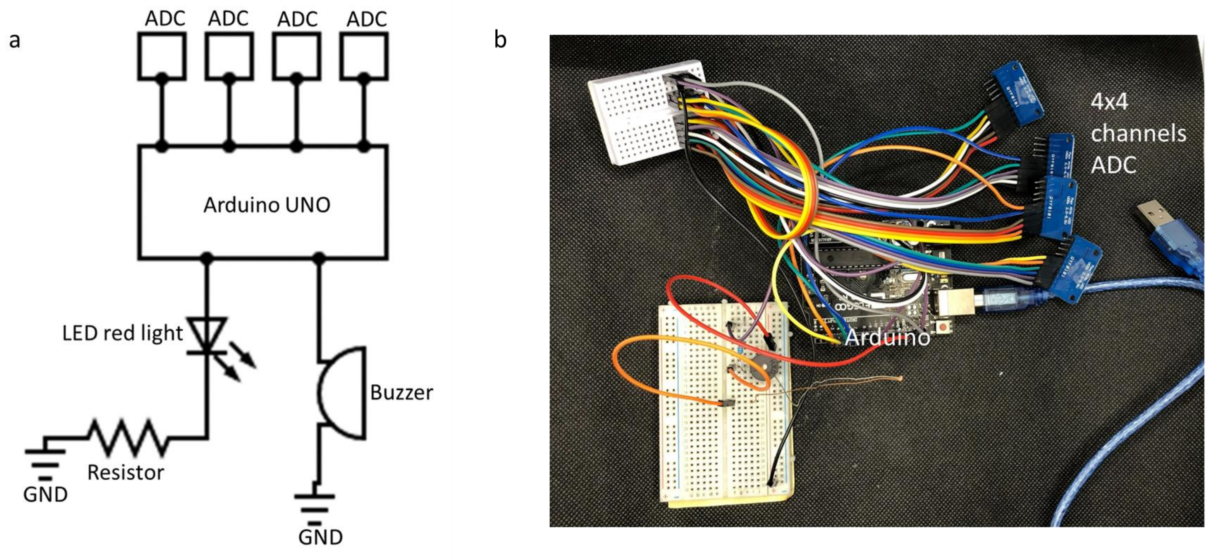

Supplementary Fig.31: (a) The circuit diagram of testing the smart conductive protection devices, and (b) the setup photo.

The setup to test the smart knee pad is shown in Supplementary Fig.31. We used a microcontroller (Arduino) to measure the output voltage for compressing the smart knee pad. The tested samples consist of four 4x4 elements, which are each connected to a four-channel ADC. Our triggered threshold was set at 900 millivolts.

Supplementary Tables

Supplementary Tab.1 Comparison of piezoelectric properties of different 3D-printed samples.

Samples	Force (N)	Voltage (V)	Ref in main article
**3D-printed cuttlebone-RS composite**	**0 ~ 0.2**	**0 ~ 9.5**	**Our work**
3D-printed self-powered pressure sensor	2.9	3 ~ 9	[3]
3D-printed piezoelectric BNNTs nanocomposites	0.1 ~ 2	1.5 ~ 15	[4]
3D-printed Ag-coated PNN-PZT ceramic-polymer grid-composite	0.2 ~ 1	0.3 ~ 2.4	[5]
3D-printed PVDF nanocomposites	0 ~ 2.5	0 ~ 3	[6]
3D printing of piezoelectric materials with designed anisotropy	0.1 ~ 0.5	0.2 ~ 1.5	[7]
Nanogenerator with non-producing kirigami structure	20 ~ 60	1 ~ 8	[8]
3D-printed piezoelectric-Regulable cells with customized electromechanical response distribution	10 ~ 100	1.5 ~ 8	[9]

Supplementary Tab.2 Comparison of density, specific toughness, specific strength, and piezoelectricity of different samples.

Samples	Density (g/cm^3)	Specific toughness ($MPam1/2/gcm^{-3}$)	Specific strength (Mpa/gcm^{-3})	Piezoelectricity	Complex shape	Ref in main article
3D-printed cuttlebone-RS composite	1.67	3.125	7.186	Yes	Yes	Our work
3D-printed nacre	1	1.5 ~ 3	60	No	Yes	[10]
RS incorporated with wood composite	1.7175	N/A	5.53 to 5.823	Yes	No	[11]
Artificial nacre	1.73 ~ 1.83	1 ~ 2	30	No	No	[12]
3D-printed American lobster Bioinspired Bouligand structure	1.2 ~ 1.4	0.769	38.462	No	Yes	[13]
3D-printed baleen's hierarchical structure	1.3	0.15 ~ 12.3	2.7	No	Yes	[13]
3D-printed Wood-Inspired Helical Composites	1.18	0.2 ~ 0.8	3.39 ~ 5.08	No	Yes	[14]

Section 2. Supplementary References:

[1] E. Lemaire, D. Thuau, J.-B. De Vaulx, N. Vaissiere, and A. Atilla, "Rochelle Salt-Based Ferroelectric and Piezoelectric Composite Produced with Simple Additive Manufacturing Techniques," *Materials,* vol. 14, no. 20, p. 6132, 2021.

[2] R. Levitskii, I. Zachek, T. Verkholyak, and A. Moina, "Dielectric, piezoelectric, and elastic properties of the Rochelle salt NaKC 4 H 4 O 6 · 4 H 2 O: A theory," *Physical Review B,* vol. 67, no. 17, p. 174112, 2003.

[3] X. Yuan, X. Gao, X. Shen, J. Yang, Z. Li, and S. Dong, "A 3D-printed, alternatively tilt-polarized PVDF-TrFE polymer with enhanced piezoelectric effect for self-powered sensor application," *Nano Energy,* vol. 85, p. 105985, 2021.

[4] J. Zhang *et al.*, "3D printed piezoelectric BNNTs nanocomposites with tunable interface and microarchitectures for self-powered conformal sensors," *Nano Energy,* vol. 77, p. 105300, 2020.

[5] A. Yan, X. Yuan, Z. Li, J. Yang, K. Ren, and S. Dong, "3D-printed flexible, multilayered ceramic-polymer composite grid with integrated structural-self-sensing function," *Sensors and Actuators A: Physical,* vol. 332, p. 113187, 2021.

[6] S. Bodkhe, G. Turcot, F. P. Gosselin, and D. Therriault, "One-step solvent evaporation-assisted 3D printing of piezoelectric PVDF nanocomposite structures," *ACS applied materials & interfaces,* vol. 9, no. 24, pp. 20833-20842, 2017.

[7] H. Cui *et al.*, "Three-dimensional printing of piezoelectric materials with designed anisotropy and directional response," *Nature materials,* vol. 18, no. 3, pp. 234-241, 2019.

[8] X. Zhou *et al.*, "All 3D-printed stretchable piezoelectric nanogenerator with non-protruding kirigami structure," *Nano Energy,* vol. 72, p. 104676, 2020.

[9] X. Liu, J. Liu, L. He, Y. Shang, and C. Zhang, "3D Printed Piezoelectric‐Regulable Cells with Customized Electromechanical Response Distribution for Intelligent Sensing," *Advanced Functional Materials,* vol. 32, no. 26, p. 2201274, 2022.

[10] Y. Yang *et al.*, "Electrically assisted 3D printing of nacre-inspired structures with self-sensing capability," *Science advances,* vol. 5, no. 4, p. eaau9490, 2019.

[11] E. Lemaire, C. Ayela, and A. Atli, "Eco-friendly materials for large area piezoelectronics: self-oriented Rochelle salt in wood," *Smart Materials and Structures,* vol. 27, no. 2, p. 025005, 2018.

[12] W. Cui *et al.*, "A strong integrated strength and toughness artificial nacre based on dopamine cross-linked graphene oxide," *ACS nano,* vol. 8, no. 9, pp. 9511-9517, 2014.

[13] W. Huang *et al.*, "Multiscale toughening mechanisms in biological materials and bioinspired designs," *Advanced Materials,* vol. 31, no. 43, p. 1901561, 2019.

[14] L. Zorzetto and D. Ruffoni, "Wood‐inspired 3D‐printed helical composites with tunable and enhanced mechanical performance," *Advanced Functional Materials,* vol. 29, no. 1, p. 1805888, 2019.
