# Supplementary material for: Growing recyclable and healable piezoelectric composites in 3D printed bioinspired structure for protective wearable sensor
Source: Nat Commun. 2023 Oct 14;14:6477. doi: 10.1038/s41467-023-41740-6 (PMC10576793; doi:10.1038/s41467-023-41740-6)
Supplement: Supplementary file 2 — Description of Additional Supplementary Files [file 41467_2023_41740_MOESM2_ESM.pdf]

Description of Additional Supplementary Files

File Name: Supplementary Movie 1

Description: 3D-printed-RSC smart knee pad test.
