# Supplementary material for: Growing recyclable and healable piezoelectric composites in 3D printed bioinspired structure for protective wearable sensor
Source: Nat Commun. 2023 Oct 14;14:6477. doi: 10.1038/s41467-023-41740-6 (PMC10576793; doi:10.1038/s41467-023-41740-6)
Supplement: Supplementary file 5 — Peer Review [file 41467_2023_41740_MOESM5_ESM.pdf]

nature portfolio

Peer Review File

Growing Recyclable and Healable Piezoelectric Composites in 3D Printed Bioinspired Structure for Protective Wearable Sensor

Open Access This file is licensed under a Creative Commons Attribution 4.0 International License, which permits use, sharing, adaptation, distribution and reproduction in any medium or format, as long as you give appropriate credit to the original author(s) and the source, provide a link to the Creative Commons license, and indicate if changes were made. In the cases where the authors are anonymous, such as is the case for the reports of anonymous peer reviewers, author attribution should be to 'Anonymous Referee' followed by a clear attribution to the source work. The images or other third party material in this file are included in the article's Creative Commons license, unless indicated otherwise in a credit line to the material. If material is not included in the article's Creative Commons license and your intended use is not permitted by statutory regulation or exceeds the permitted use, you will need to obtain permission directly from the copyright holder. To view a copy of this license, visit http://creativecommons.org/licenses/by/4.0/.

Editorial Note: Parts of this Peer Review File have been redacted as indicated to remove third-party material where no permission to publish could be obtained.

REVIEWER COMMENTS

Reviewer #1 (Remarks to the Author):

The authors report a strategy to grow the recyclable and healable piezoelectric Rochelle salt crystals in 3D-printed cuttlebone-inspired structures to form a composite with good mechanical and piezoelectric performance. The biomimetic concept is interesting. The proposed device shows promising applications in smart monitoring devices with integrated mechanical protection and sensing ability. Although the idea and concept is attractive, there is not enough on the characterization and analysis. The following issues should be clarified to improve the quality of the manuscript.

1. It seems the structure sizes of the bio-inspired 3D-printed structure are much bigger than the cuttlefish bone structure. How the size effect influence the property of the device. The authors should perform a deeper analysis.
2. The authors state the cuttlebone structure suffers the least deformation compared to the other three structures. However, the piezoelectric output originates from the deformation of the composite. If so, I do not think the cuttlebone structure is benefit to achieve optimized piezoelectric performance.
3. The piezoelectric test should be retest using a standard test method. Such as the sensitivity, pressure range, cycle testing and frequency performance. In the manuscript, stainless weight loadings (5g - 20g) were applied to the composites along the vertical direction. However, such testing will produce obvious triboelectric outputs. It cannot confirm the outputs originate from piezoelectricity.
4. How to measure the effective piezoelectric coefficient d33 of nanocomposite film by a d33 machine. The detailed descriptions and test images should supply in the revised manuscript.
5. The piezoelectric and ferroelectric properties of piezoelectric films based on Rochelle salt crystal (RS) should be test and compared with the structured devices. Furthermore, the poling parameters, range of sensing temperatures should be provided.

6. For the healing performance, as the device consists photocurable resin, electrodes and piezoelectric Rochelle salt. How to achieve full-healing ability in the practical application.
7. How about the durability of the composite device. Especially under high pressure or cycle testing, how to avoid the interfacial separation and crack propagation for the electrodes or piezoelectric layers.

Reviewer #2 (Remarks to the Author):

Review of "Growing Recyclable and Healable Piezoelectric Composites in 3D Printed Bioinspired Structure for Protective Wearable Sensor"

-> What are the noteworthy results?

The authors report a method to grow polycrystalline Rochelle (RS) salt in bio-inspired 3D-printed cuttlebone structures to form a composite that can be repaired and acts as a smart monitoring sensor.
The study of mechanical properties of the composite is really instructive. Composite repairability is a truly original and interesting approach. Moreover, the applications and use cases are well thought out and well presented.

-> Will the work be of significance to the field and related fields? How does it compare to the established literature? If the work is not original, please provide relevant references.

The authors mentioned previous work on RS/wood-based composite, but they forget to mention that the growth of Rochelle salt in a 3D printed structure has already been reported [A]. Moreover, RS/cellulose based composites have already been demonstrated [B].

[A]: Lemaire, E., Thuau, D., De Vaulx, J. B., Vaissiere, N., & Atilla, A. (2021). Rochelle Salt-Based Ferroelectric and Piezoelectric Composite Produced with Simple Additive

Manufacturing Techniques. Materials, 14(20), 6132.

[B]: Lemaire, E., Moser, R., Borsa, C. J., & Briand, D. (2016). Green paper-based piezoelectronics for sensors and actuators. Sensors and Actuators A: Physical, 244, 285-291.

-> Does the work support the conclusions and claims, or is additional evidence needed?

The authors used polycrystalline Rochelle salt and explain its piezoelectric behavior, for example here:
{line174}"The slices are cut into different shapes according to the specific application, such as expander or shear vibration plates[22]. In our study, since no cut was conducted, the effective d33 of the final structure is the combination of three shear piezoelectric constants. The measured effective d33 is approximately ~30 pC/N, which fits within the theoretical range."

For me it's still not clear in any case how direct and converse piezoelectricity behaves in disorganized polycrystalline Rochelle salt... Further studies should be done, but maybe that's not the goal principal of this work, but authors should convincingly demonstrate the piezoelectric behavior of their devices.
Piezoelectricity includes direct and converse effects. Thus, authors should consider several direct and inverse comparative measurements of effective d33 to confirm piezoelectricity (both effects) and range of coefficients in their composite samples. One should expect a distribution of coefficients. In addition, the coupling factor of the samples could be measured.

-> Are there any flaws in the data analysis, interpretation and conclusions? Do these prohibit publication or require revision?

The effective converse [pm/V] and direct [pC/N] d33 should be measured on several sample and compared with respect to a relevant choosen parameter (RS loading, grain size,

impedance etc.). Output voltage only is not sufficient to qualify piezoelectric properties and samples comparative performances.
This work require revision.

NB: for example, triboelectric effects in some sample could produce quite similar output signals (without any piezoelectricity). Thus, piezoelectricity must be demonstrated systematically (e.g. measuring direct and converse piezoelectric effects).

-> Is the methodology sound? Does the work meet the expected standards in your field?

In terms of methodology, the authors should consider additional characterizations (related to piezoelectricity):
- comparison of complex impedance, current or d33, from one sample to another.
- direct and converse piezoelectricity should be highlighted.
- the piezoelectric coupling factor of fabricated samples should also be documented.

-> Is there enough detail provided in the methods for the work to be reproduced?

The fabrication process and raw characterizations are well documented in the manuscript and in the supplementary data. Sufficient details are provided regarding reproducibility.

Reviewer #3 (Remarks to the Author):
The authors present newly manufactured multifunctional sensors that are also mechanically strong and thus can be used in knee caps and armors supposedly for sports applications. Rochelle salt, an ecofriendly material is used as a piezoelectric sensing element, which were grown on a 3D printed polymeric scaffoldic structures. However, there are serious flaws in the procedures and test results presented in this work and I recommend that this article should not be accepted for publication in the reputed Nature communications journal.

- Will the work be of significance to the field and related fields? How does it compare to the established literature? If the work is not original, please provide relevant references.
Ans: Not in its current state.

- Does the work support the conclusions and claims, or is additional evidence needed?
Ans: No.

- Are there any flaws in the data analysis, interpretation and conclusions? - Do these prohibit publication or require revision?
Ans: There are major flaws in the testing, data analysis as well as fabrication and methodology of the article. This article should not be published in this present state.
1. It is not clear as the shown graphs are piezoelectricity or of a combination of piezoelectricity and triboelectricity. Have the authors checked for this difference in the phenomenon by reversing the electrodes as explained in the article: (Šutka, Sherrell et al. 2020)
2. What are “three different volume ratios of 40%, 60%, and 80% of 3D-printed cuttlebone polymer structures without RS crystal, and with grown RS crystals for 24 hours.” And how are they prepared? This is missing in the methods section.
3. Fig.1: Where is the XPS curve for the images in 1b, how can you prove that all that is coming from the same sample? What is the proportion of various elements?
4. What was the applied mechanical signal for the response shown in Fig.5?
5. How many samples of each kind were tested in the mechanical tests? What were their dimensions? The data presented should include a statically correct amount of tested samples.
6. Through lines 286 and beyond, the authors wrongly claim their process as recyclable, where they are reusing the salt solution for new 3D printed scaffolds. This is not regeneration.
7. Many confusing statements are present in the article and need to be corrected. For example, here are a few of them.
a) Line145: “Stainless <<What?>>weight loadings (5g - 20g) were applied to”??
b) Line146: “the 20%-80% composites along the vertical” What is 20% and what is 80% in these composites?

c) Line198: “The cuttlebone structure suffers the least deformation and homogeneous force distribution compared to the other three structures when subjected to the same compressive force.”

a) Line452 in the article: The cuttlebone structure, triangular, cubic, honeycomb structure, and 4 × 4 Array armors with cuttlebone structures in each element

a) Line463 in the article: “For each layer to be able to be attached to the print platform, 2.5 seconds of exposure time was set, followed by 35 seconds of exposure time at the bottom.” What does that mean? The procedure is unclear.

8. Please correct Figure S1 title, “Fig. S.3 The CT scanned photos of 20% cuttlebone polymer structure grown RS crystal for 24 hours. (Scale bar, 50mm)” there is only one photo in the figure.

- Is the methodology sound? Does the work meet the expected standards in your field?

Ans: No the methodology is unclear and irreproducible in its current state. The work does not meet the standards in the field of 3D printing. The procedure to 3D print and available commercial resin and then growing Rochelle salt crystals in it doesn’t represent the novelty in 3D printing process at all. Also, growing Rochelle salt crystals in 3D printed scaffolds has already been published (Lemaire, Thuau et al. 2021). Hence the novelty of this work is questionable.

- Is there enough detail provided in the methods for the work to be reproduced?

Ans: No. The methodology lacks details. As well as the results in Fig2 are coming from only singular tests, with no proof of repeatability or accuracy of the sensors presented? Do the sensors generate the same signal with the dropping of the same weights multiple times? Is missing in the results and discussions. The FEM analysis and the inputs and model specifications are not clear. What do you mean by X1,X2 and X3 axes? How do they correspond to the global access? How were the properties for the RS that were supplied to the model, obtained? Were they from literature, or did the authors carry out experiment’s

to determine them? In either cases, all the input parameters and values to the model should be presented in the article.

References:

Lemaire, E., D. Thuau, J. B. De Vaulx, N. Vaissiere and A. Atilla (2021). "Rochelle Salt-Based Ferroelectric and Piezoelectric Composite Produced with Simple Additive Manufacturing Techniques." Materials (Basel) 14(20).

Šutka, A., P. C. Sherrell, N. A. Shepelin, L. Lapčinskis, K. Mālnieks and A. V. Ellis (2020). "Measuring Piezoelectric Output—Fact or Friction?" 32(32): 2002979.

9.

Response Letter to Reviewer comments

Reviewer #1 (Remarks to the Author):

The authors report a strategy to grow the recyclable and healable piezoelectric Rochelle salt crystals in 3D-printed cuttlebone-inspired structures to form a composite with good mechanical and piezoelectric performance. The biomimetic concept is interesting. The proposed device shows promising applications in smart monitoring devices with integrated mechanical protection and sensing ability. Although the idea and concept is attractive, there is not enough on the characterization and analysis. The following issues should be clarified to improve the quality of the manuscript.

[author reply]: We are grateful to the reviewer for the comment.

1. It seems the structure sizes of the bio-inspired 3D-printed structure are much bigger than the cuttlefish bone structure. How the size effect influence the property of the device. The authors should perform a deeper analysis.

[author reply]: We are thankful to the reviewer for this question. Our work involved developing 3D-printed Rochelle salt cuttlebone composite (RSC) using the Phrozen Sonic MINI 4K 3D printer, which has a resolution of 35 micrometers, and using Aqua-Gray 4K resin. In order to make the 3D-printed cuttlebone as close as possible to the natural cuttlefish bone size, three different sample scales were added in the revised manuscript and produced with a Micro-SLA 3D printer. As for the resin, it is UHR Kudo 3D Inc. Micro-SLfA 3D printers are capable of producing objects with a resolution of up to 20 micrometers. For electrical and mechanical comparisons, we 3D-printed three different sizes of samples from the Cuttlebone model to mimic the original size of the Cuttlefish bone in nature. As shown in **Fig. R1(a)**, from left to right, the first photo shows a sample of the same size as the one printed with the Phrozen 3D printer in the article. As can be seen in the second and third photos, the sample has been scaled down by 1/4 and 1/16, respectively. All three different scale samples have the same size of 10×10×3mm. According to **Fig.R1(b)**, the different scales of 3D-printed-RSC correspond to **Fig.R1(a)**. As can be seen in the photograph (**Fig.R1(c)**) taken under the microscope, the Rochelle salt crystals are dense and uniformly distributed throughout the 3D-printed cuttlebone.

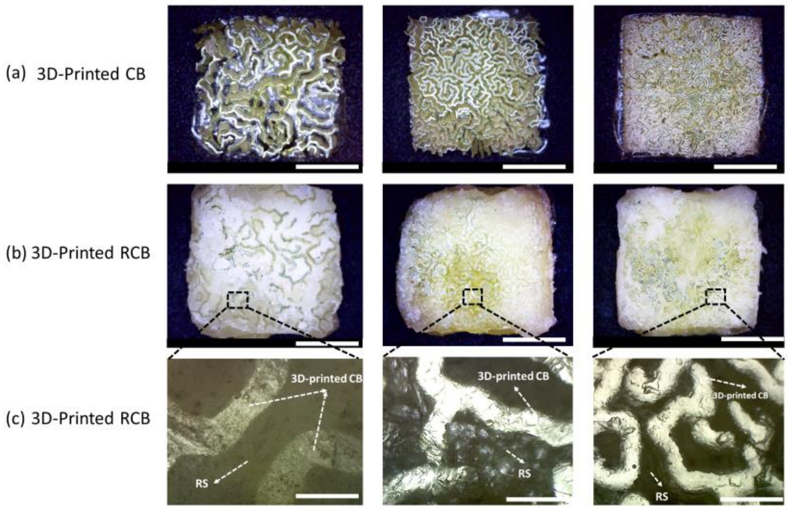

Fig.R1: 3D-printed-RSC with different scales.
(a) 3D-printed CB with same 20% cuttlebone ratio but different scales. (scale bars: 5mm) (b) 3D-printed-RSC with same 20% cuttlebone ratio but different scales. (scale bars: 5mm) (c) Microscope photos of 3D-printed RCB with same 20% cuttlebone ratio but different scales. (scale bars: 500µm)

As a follow-up to the preparation of the test 3D-printed-RSC samples, we used the same method to conduct a single output voltage measurement using weights. The varying scales of 3D-printed-RSC samples were laid parallel to the horizontal table, and a weight of 20g was dropped freely at a height of 50 mm perpendicular to the sample. The output voltage of the sample was recorded as a result of force. **Fig. R.2(a)** records the output voltages. As the RS ratio does not change regardless of the scale of the samples (20% 3D-Printed-Cuttlebone, 80% RS), and piezoelectric properties are provided by the RS, there is essentially no change in properties with no change in the piezo-ratio.
Based **on Fig. R.2(b)**, the sample size for the three-point bending test is 10×5×3mm. Compared to the 3D-printed-RSC samples printed out by the Phrozen 3D printer in the article, the strain-stress graph indicates lower maximum stress. The significant differences in mechanical properties can be attributed to the different resins used by the two printers to achieve the highest degree of accuracy. It is possible to compare the mechanical properties of different scales of 3D-printed-RSCs when they are printed using the same resin conditions**.** According to Fig. R.2(b), the sample downsized by 1/16 has slightly lower strength than the original scale and the 1/4 scale samples. Due to the narrower free space, the 1/16 scale sample contains smaller RS crystals. The smaller RS crystals are subjected to less force as well as cracking faster when bent. As a result of limitations in the accuracy of the 3D printer at present, although we have reached the micron stage, the smallest size has not yet been printed.

At this stage, due to the limitations of the printing accuracy, it is difficult to print cuttlefish bone walls thinner than 5 microns. Future research will examine 3d printing of high-precision and large-scale sample in order to achieve biomimicry on a micron or even nanometer scale.

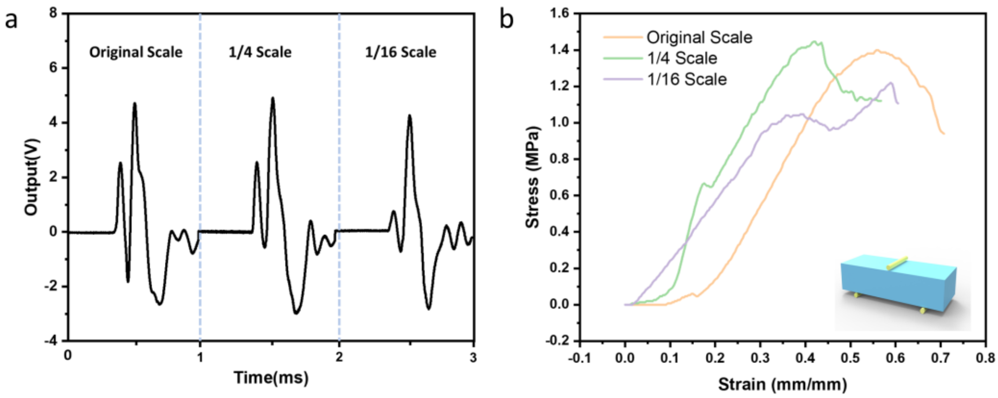

Fig.R.2: Different scales 3D-printed-RSC piezoelectric and mechanical properties.
(a) Single drop output voltages of 20g weights of original scale, 1/4 scale, 1/16 scale, respectively. (b) Strain-stress graphs of original scale, 1/4 scale, 1/16 scale, respectively.

2. The authors state the cuttlebone structure suffers the least deformation compared to the other three structures. However, the piezoelectric output originates from the deformation of the composite. If so, I do not think the cuttlebone structure is benefit to achieve optimized piezoelectric performance.

[author reply]: We are thankful to the reviewer for pointing this question out. For the applications as protective devices such as armor for athletes and knee pads for the elderly people, the high performance of mechanical properties are one of our primary concerns. The mechanical properties of the cuttlefish bone structure were examined experimentally and through simulation analysis together, and the results indicate that it has the highest mechanical properties compared to the other three structures. As for the piezoelectric performance, we acknowledge that the sample does not perform well in comparison with the rest of the structure. However, as can be seen from **Fig. R.3**, the output of cuttlebone sample is not significantly lower than the other three composites under the impact of 2g weight. The 3D-printed-RSC applications in this study, instead, focus on piezoelectric sensors, which can be used as a smart armor to normalize the output voltage in order to obtain the corresponding force in equal proportions. The piezoelectric sensing threshold of a smart knee pad for the elderly people can be preset, and an alarm device can be activated when the threshold value has been exceeded.
Combining the above two considerations, we determine that the mechanical performance is of the utmost importance, and the piezoelectric performance is applicable to the sensing function, thereby choosing 3D-printed-RSC as the ideal solution.

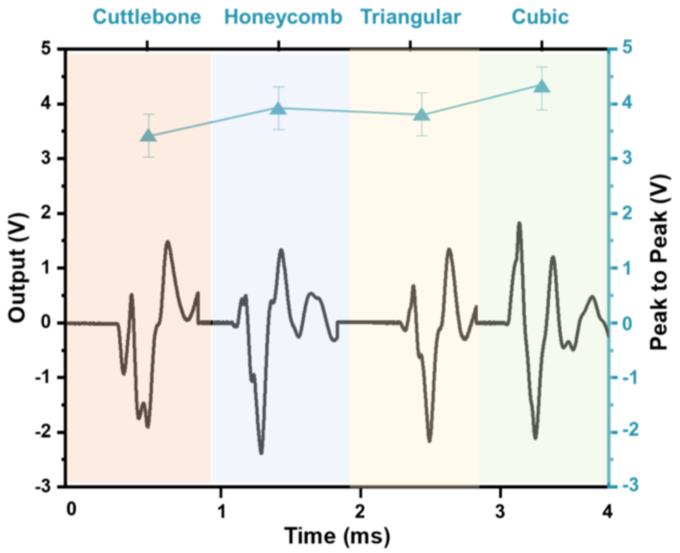

Fig. R.3: Output voltage of Cuttlebone, Honeycomb, Triangular, and Cubic structures.

3. The piezoelectric test should be retest using a standard test method. Such as the sensitivity, pressure range, cycle testing and frequency performance. In the manuscript, stainless weight loadings (5g - 20g) were applied to the composites along the vertical direction. However, such testing will produce obvious triboelectric outputs. It cannot confirm the outputs originate from piezoelectricity.

[author reply]: We sincerely thanks the reviewer for this suggestion. To obtain more precise piezoelectric performance data, cyclic tests were added to the experiments. The **Fig. R.4(a)** shows the motor-driven cyclic impact test set-up. The motor drives the rotation of the rocker arm, and the frequency as well as force level are adjusted by changing the motor input voltage. The bottom of the installation pulley allows the distance between the tested 3D-printed-RSC samples to be easily adjusted. For the purpose of obtaining the output voltage signal, the positive and negative terminals of the 3D-printed-RSC are connected to the signal collector. In order to obtain accurate values of the magnitude of the forces on the tested sample, we apply the thin film force sensor as shown in **Fig. R.4(a)**. From 1Hz to 4Hz, **Fig. R.4(b)** illustrates how voltage is generated by continuous impact on a 3D-printed RSC. The sensitivity of the composite can be observed in **Fig. R.4(c)** according to the peak-to-peak output voltage under varying forces.

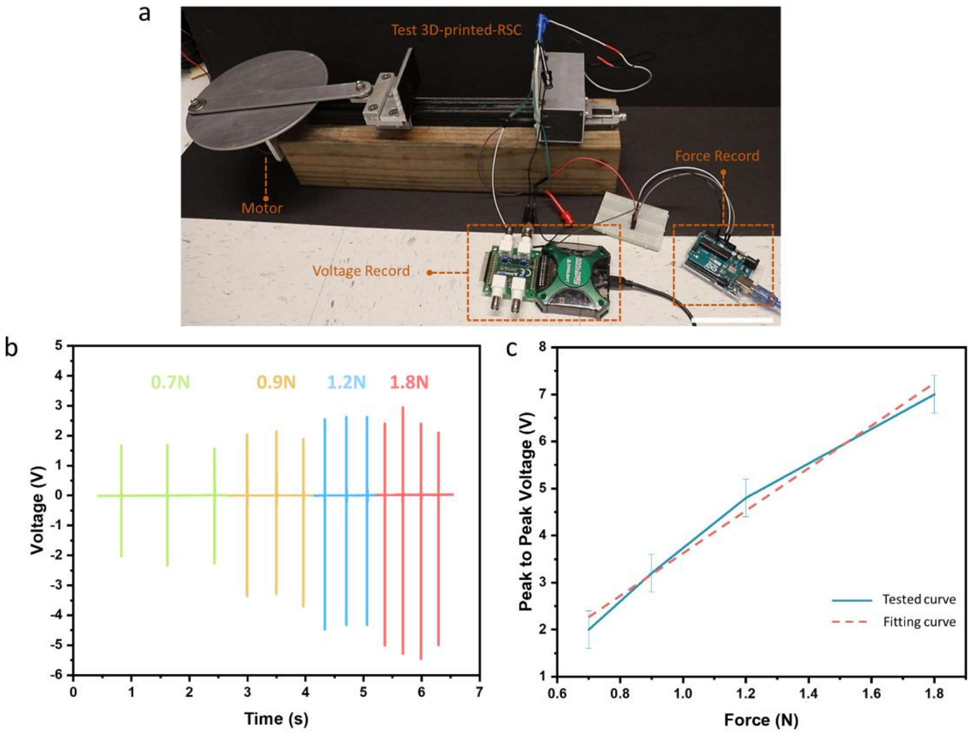

Fig. R.4: Cycle testing performance.
(a) Photo of test setup. (scale bar: 10cm); (b) Piezoelectric output at different frequencies corresponding to different force magnitudes; (c) Peak to peak voltage output at different force.

To demonstrate that the output of the sample is derived from piezoelectric effects rather than triboelectric effects, similarly, we used a 20g weight to hit the composites at a distance of 5cm vertically in order to measure the output voltage. The results of **Fig. R.13** indicate that, as compared to the more obvious output voltage signal of the 3D-printed RSC, the epoxy and silicone rubber samples did not exhibit any significant voltage output after being hit by the weights. Additionally, we used the MSA-500 laser vibrometer to obtain displacement and phase data as shown in **Fig. R.14**. These illustrations show that Rochelle salt composites exhibit piezoelectric properties.

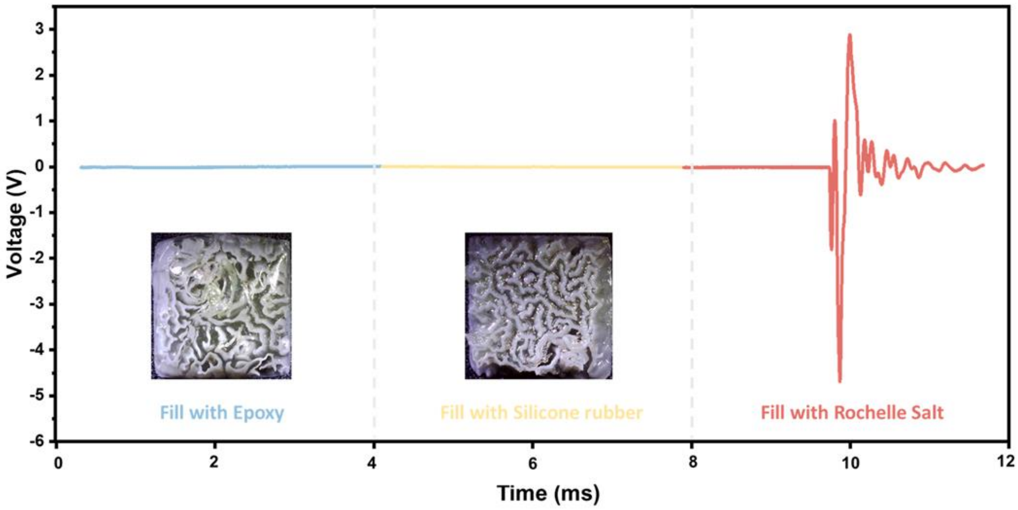

Fig. R.13: Voltage output graphs of 3D-printed Cuttlebone fill with different materials

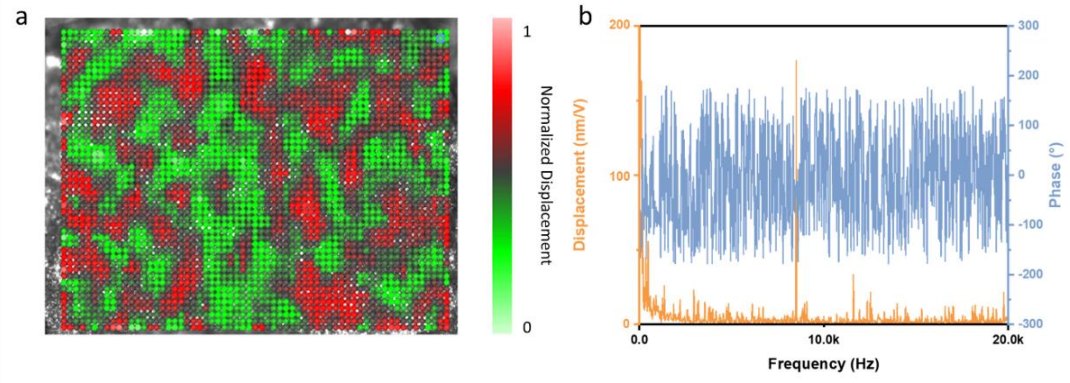

Fig. R.14: Displacement and phase of 3D-printed-RSC measured using a laser vibrometer

(a) Normalized displacement distribution under microscope; (b) Displacement module and phase of 3D-printed-RSC graph

4. How to measure the effective piezoelectric coefficient d_{33} of nanocomposite film by a d_{33} machine. The detailed descriptions and test images should supply in the revised manuscript.

[author reply]: We are thankful to the reviewer for this question. For d_{33} measurement, the direct piezoelectric coefficient (d_{33}) of the samples have been characterized by a d_{33} meter (YE2730A, APC international, Ltd., Mackeyville, PA, USA) as shown in **Fig.R.5**. The tested samples were placed on the fixture of the meter, and then the d_{33} value can be recorded. **Fig. R.5** illustrates 25pC/N of one of the d_{33} tests. The Rochelle Salt composite with volume ratio of 20% was first placed on the fixture of the d_{33} meter, showing the value of 22 - 32 pC/N. Afterwards, when the sample was placed upside-down, the d_{33} value was recorded as (-32) - (-25) pC/N. It illustrated the piezoelectricity of the Rochelle salt composite.

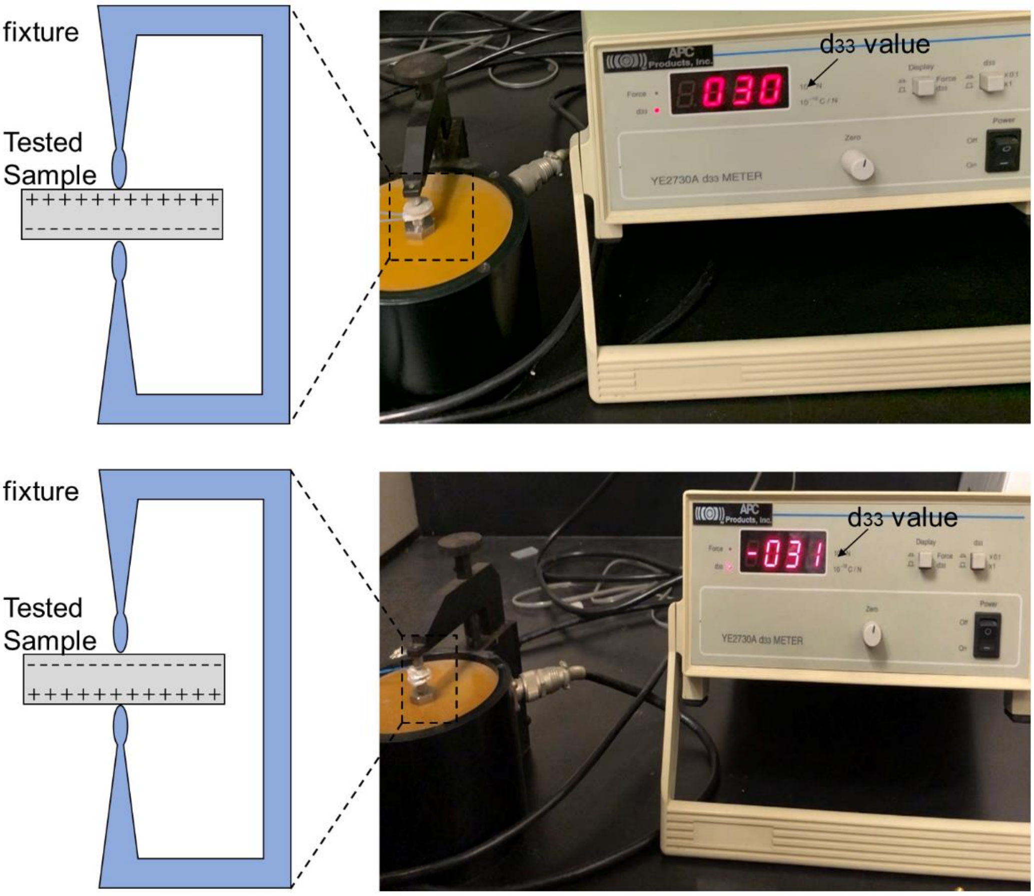

Fig. R. 5: d_{33} measurement setup.

5. The piezoelectric and ferroelectric properties of piezoelectric films based on Rochelle salt crystal (RS) should be test and compared with the structured devices. Furthermore, the poling parameters, range of sensing temperatures should be provided.

[author reply]: We are thankful to the reviewer for this insightful comment. An RS piezoelectric film is formed by dropping the RS solution onto the copper sheet, with the copper sheet acting as both an electrode and the other electrode as an E-solder attached to the RS surface after 24 hours of growth as shown in **Fig. R.6(a)**. **Fig. R.6(b)** demonstrates the output voltage of a RS piezoelectric film subjected to a cyclic impact test at a frequency of 2 Hz.
Moreover, we compared RS crystals for piezoelectric testing (**Fig. R.6(c)**). We found that the pure RS crystal is more fragile after five cycles of impact testing due to the lack of a 3D-printed cuttlebone as a mechanical support (**Fig. R.6(d)**). As reported in this paper, manufactured3D-printed-RSC samples exhibit good piezoelectric properties without the need for any poling step. It is a natural property of Rochelle salt that it exhibits piezoelectricity without the need for poling. With three non-neglectable orthogonal piezoelectric coefficients in RS (d_{14}, d_{25}, d_{36}), piezoelectric responses can be measured in almost all axes [1]. Levitskii et al.[2] illustrated RS crystals will decompose at approximately 55 °C, and the polar ferroelectric phase, occurring at the Curie temperature range between −18°C and +24°C, is monoclinic. Our experiments were conducted in a room temperature environment (20°C).

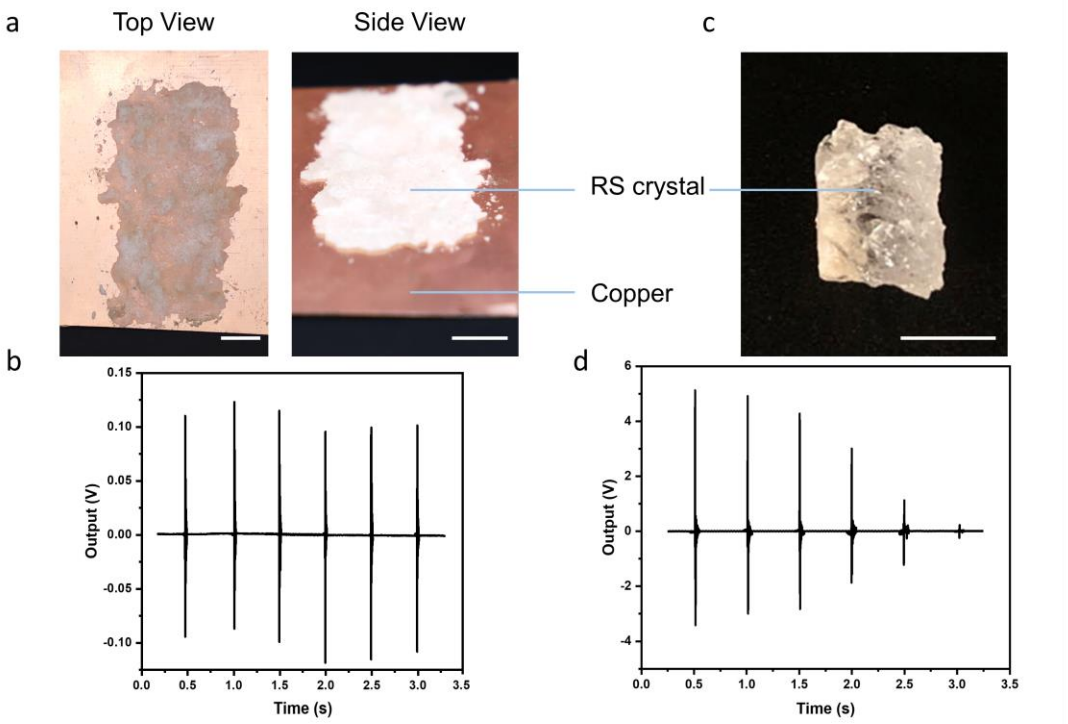

Fig. R.6: RS piezo film and RS piezo crystal property.
(a) Photos of RS piezo film (scale bars: 1cm); (b) Output voltage under 2Hz frequency of RS piezo film; (c) Photos of RS piezo crystal (scale bar: 1cm); (d) Output voltage under 2Hz frequency of RS piezo crystal.

6. For the healing performance, as the device consists photocurable resin, electrodes and piezoelectric Rochelle salt. How to achieve full-healing ability in the practical application.

[author reply]: We are thankful to the reviewer for this point. In this study, we fabricate the 3D-printed cuttlebone by using Phrozen Aqua 4K 3D printing resin, primarily composed of Dipropylene Glycol Diacrylate (DPGDA). Although this commercial material has low viscosity and fast curing features, it does not exhibit self-healing properties. While different strategies are used to create self-healing polymers with high mechanical properties, self-healing ability, or environmental stability, there are many high-performance self-healing polymers that are available. As an example, Snyder et al. [3] had fabricated a fiber-composite that allowed for in situ healing on a minute scale and for an extended period of time, and in a study reported by Cheng et al.[4], a photo-irradiation approach to resin-based self-healing was described. As a result, within five minutes, the damaged area of the polymer film self-healed, indicating that the generated polyurethanes exhibit high-temperature resistance around 1000 °C, as well as significant self-healing properties. Moreover, Self-healed electrodes are also being extensively studied, Sim et al. [5] have also reported the development of a self-healing electrode comprised of liquid crystal graphene oxide (LCGO) and silver nanowires (AgNWs) that is electrically conductive, mechanically robust, and printable. Based on the other previous studies, we aim to achieve full-healing ability in the 3D-printed-RSC devices in future research.

7. How about the durability of the composite device. Especially under high pressure or cycle testing, how to avoid the **interfacial separation and crack propagation** for the electrodes or piezoelectric layers.

[author reply]: We are grateful for the reviewer’s comment. We added a series of sustained impact tests during 4000 seconds under 2 Hz in order to verify the durability of the 3D-printed RSC device. **Fig. R.7** demonstrates that the 3D-printed-RSC devices perform well and maintain a stable output voltage from 0 to 6800 cycles. A fracture was observed in the sample at about 6800 cycles with RS crystal detachment.

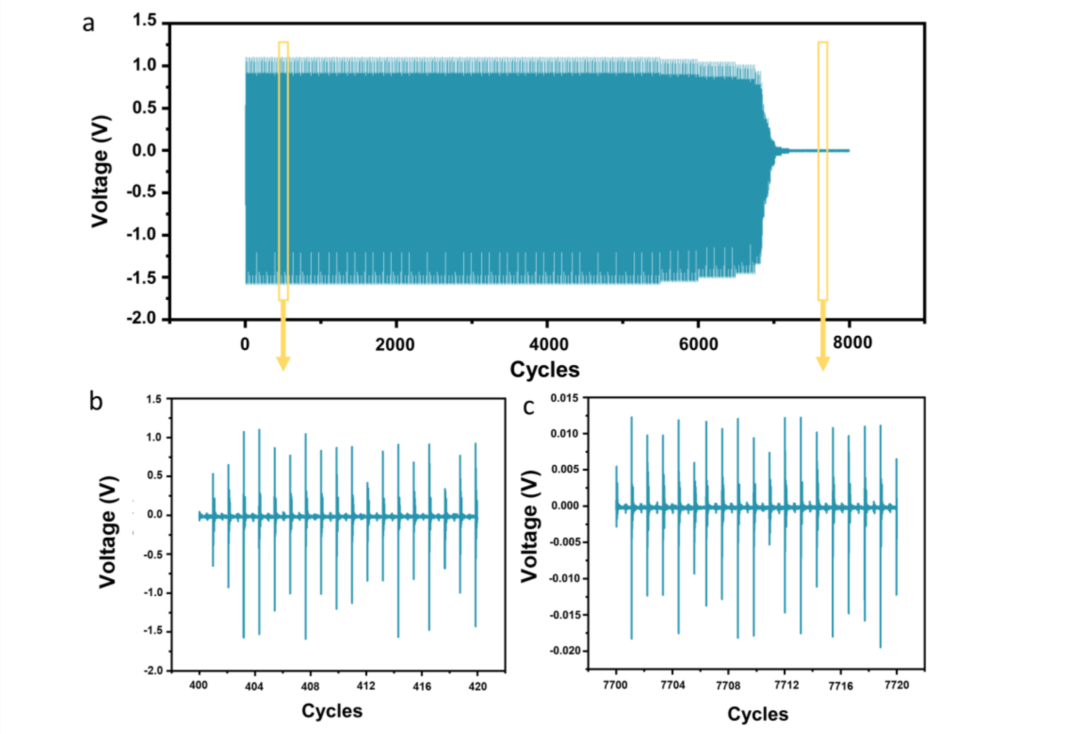

Fig. R.7: 8000 cycles cyclic impact test output voltage graphs.
(a) The output voltage over 8000 cycles cyclic impact test under 2 Hz frequency; (b) The graph of zoom in between 400 and 420 cycles; (c) The graph of zoom in between 7700 and 7900 cycles.

A complete fracture occurred at approximately 7000 cycles. To avoid the interfacial separation and crack propagation, it is possible to attach a functionalization agent to the sample surface[6]. Optimal surface coverage is achieved by optimizing the surface functionalization reaction. A sterically hindered surface is created by these covalent bonds between piezoelectric nanoparticles and the polymer matrix network. This improves the interfacial bonding and dispersion quality of highly concentrated piezo-active colloidal resin In the event that the composite has been damaged (**Fig. R.8**), the 3D-printed-RSC can be repaired in accordance with its healable and recyclable characteristics, so that it can be used for a longer period of time, and in future studies, it is possible to add a protective coating to the outside of the composite sensor to extend its useful life and reduce operating temperatures.

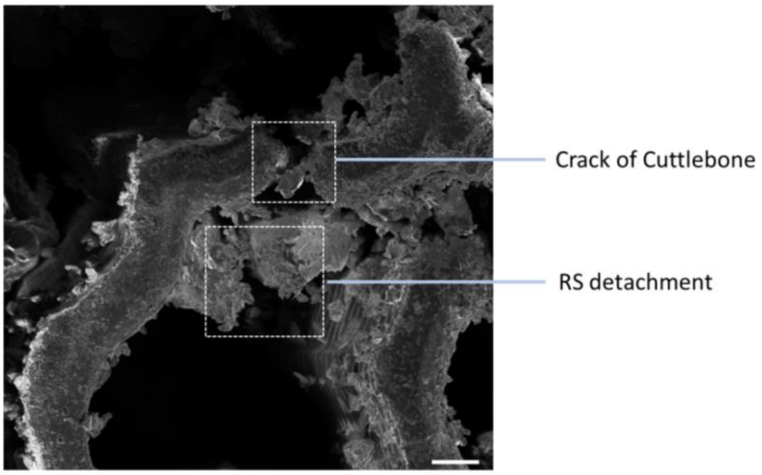

Fig. R.8: SEM photo of 3D-printed-RSC after 8000 times cyclic impact test (scale bar: 300µm).

Reviewer #2 (Remarks to the Author):

Review of "Growing Recyclable and Healable Piezoelectric Composites in 3D Printed Bioinspired Structure for Protective Wearable Sensor"

-> What are the noteworthy results?

The authors report a method to grow polycrystalline Rochelle (RS) salt in bio-inspired 3D-printed cuttlebone structures to form a composite that can be repaired and acts as a smart monitoring sensor.
The study of mechanical properties of the composite is really instructive. Composite repairability is a truly original and interesting approach. Moreover, the applications and use cases are well thought out and well presented.

[Author reply]: We thank the reviewer for the great comment.

-> **Will the work be of significance to the field and related fields?** How does it compare to the established literature? If the work is not original, please provide relevant references.

The authors mentioned previous work on RS/wood-based composite, but they forget to mention that the growth of Rochelle salt in a 3D printed structure has already been reported [A]. Moreover, RS/cellulose based composites have already been demonstrated [B].

[A]: Lemaire, E., Thuau, D., De Vaulx, J. B., Vaissiere, N., & Atilla, A. (2021). Rochelle Salt-Based Ferroelectric and Piezoelectric Composite Produced with Simple Additive Manufacturing Techniques. Materials, 14(20), 6132.

[B]: Lemaire, E., Moser, R., Borsa, C. J., & Briand, D. (2016). Green paper-based piezoelectronics for sensors and actuators. Sensors and Actuators A: Physical, 244, 285-291.

[author reply]: We are thankful to the reviewer for pointing this question out. Initially, we were inspired by Dr. Lemaire's research on RS composites[1, 7]. Through the growth of RS on paper, in wood, and in a 3D-printed basic frame, he extensively analyzed the piezoelectric properties of the composites. As a result of Dr. Lemaire's inspiration, we did this work with the intention of adding higher mechanical properties to the composites based on their piezoelectric properties.

A cuttlefish skeleton has to withstand greater pressure because of its survival in the deep ocean, and we were simultaneously inspired by its structure in nature. As a final step, we combined the piezoelectric properties of the RS crystal with the mechanical properties of the cuttlefish bone structure in order to fabricate the 3D-printed-RSC to realize multifunctional applications.

The growth mechanism and piezoelectric performance with crystallographic direction of RS crystals inside different 3D printed cuttlebone-inspired structure were studied in this paper. The optimized printed composites with growing crystals (especially 20 vol% polymer) show excellent specific toughness of 3.125 MPam1/2/gcm-3. In addition to its outstanding mechanical and piezoelectric performance, the growth mechanisms, the recyclability, and repairability of the RS crystals in 3D printed bionic device were studied. The new recycled 3D-printed cuttlebone RS composite can achieve recovery percentage of over 90% in piezoelectric and mechanical properties, illustrating an excellent sustainability of applying RS composite. Moreover, the smart array armor as well as the knee pad based on the 3D-printed cuttlebone-RS composite can be recycled, healed, and achieved for the detection of the location and magnitude of the force experienced by the wearers.

-> Does the work support the conclusions and claims, or is additional evidence needed?

The authors used polycrystalline Rochelle salt and explain its piezoelectric behavior, for example here:
"The slices are cut into different shapes according to the specific application, such as expander or shear vibration plates [22]. In our study, since no cut was conducted, the effective d_{33} of the final structure is the combination of three shear piezoelectric constants. The measured effective d_{33} is approximately ~30 pC/N, which fits within the theoretical range."

For me it's still not clear in any case how **direct and converse** piezoelectricity behaves in disorganized polycrystalline Rochelle salt... Further studies should be done, but maybe that's not the goal principal of this work, but authors should convincingly demonstrate the piezoelectric behavior of their devices.
Piezoelectricity includes direct and converse effects. Thus, authors should consider several direct and inverse comparative measurements of effective d_{33} to confirm piezoelectricity (both effects) and range of coefficients in their composite samples. One should expect a distribution of **coefficients**. In addition, the **coupling factor** of the samples could be measured.

[Response letter]: We are grateful that reviewer has pointed out these problems. For d_{33} measurement, the direct piezoelectric coefficients (d_{33}) of the samples have been characterized by d_{33} meter (YE2730A, APC international, Ltd., Mackeyville, PA, USA) as the **Fig.R.9** below shows. The tested samples were placed on the fixture of the meter, and then the d_{33} value can be recorded. The 3D-printed RSC with volume ratio of 20% was first placed on the fixture of the d_{33} meter, showing the value of 30 pC/N. Afterwards, when the sample was placed upside-down, the d_{33} value was recorded as -31 pC/N. It illustrated the piezoelectricity of the Rochelle salt composite.

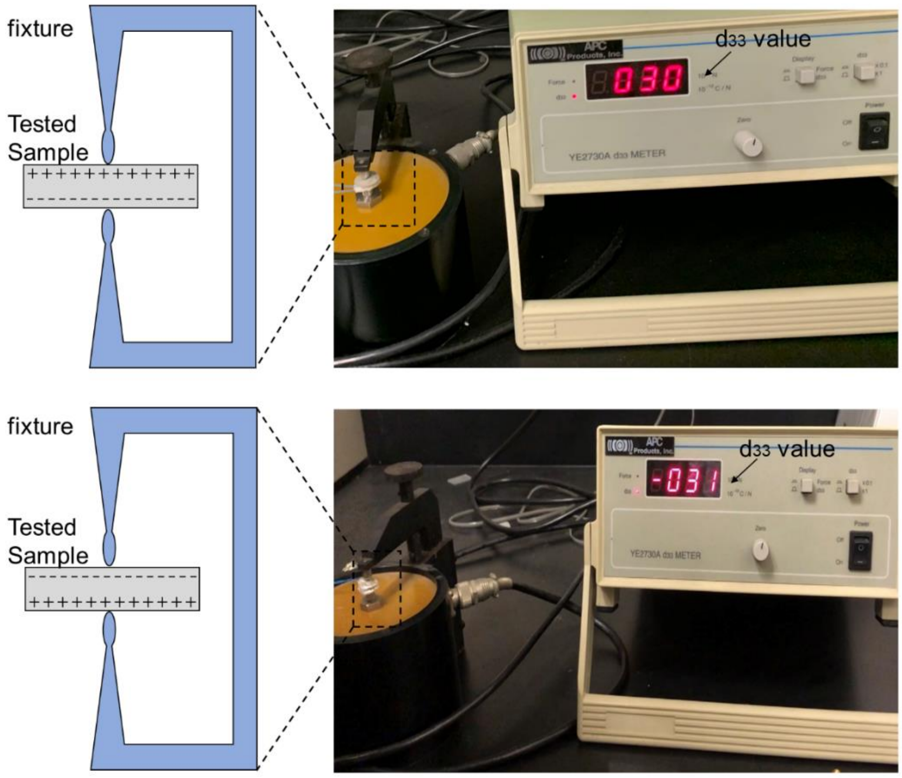

Fig. R. 9: d33 measurement setup.

To test the converse piezoelectricity, the static displacements were calculated for several applied voltages by using MSA-500 laser vibrometer from Polytech (**Fig. R. 10**), from 1V to 10V, under 5 kHz is shown in **Fig. R.11**. The statistic displacement increased with the applied voltage, and we performed a linear fit to the resulting curve, and the slope of the fitted curve is the converse d_{33}. This results in the direct and converse d_{33} values remaining essentially the same.

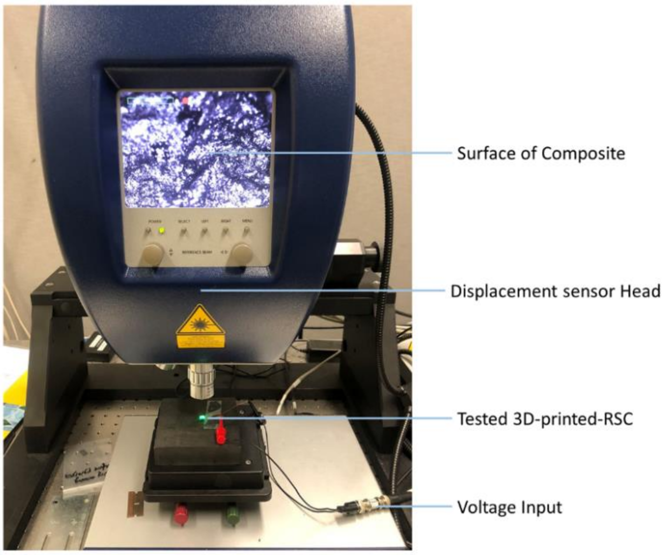

Fig. R.10: MSA-500 laser vibrometer test machine setup.

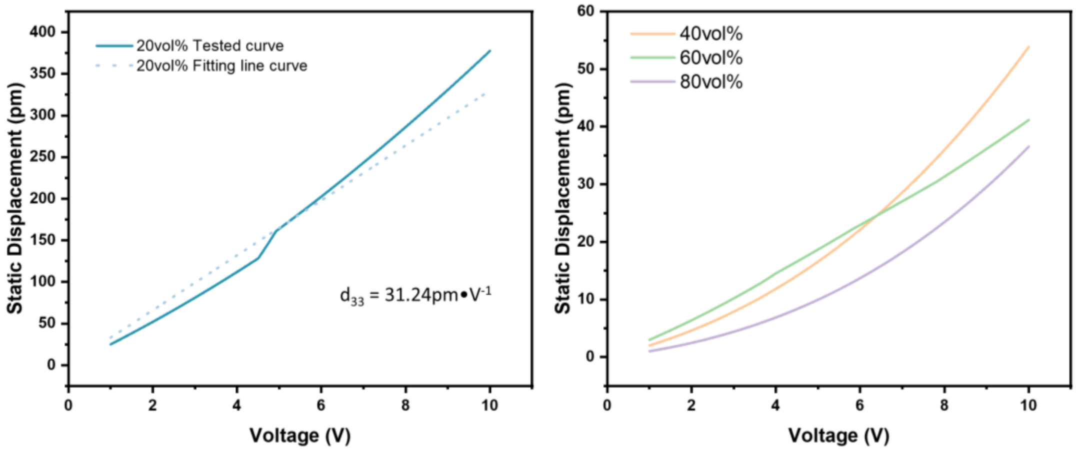

Fig. R.11: Static displacement vs. applied voltage measured on 20%, 40%, 60%, 80% composites.

Moreover, we have also tested the d_{33} of the Rochelle salt composite in different volume ratio, showing the range of the piezoelectric coefficients (**Fig** below, **Fig. 2(e)** in the manuscript).

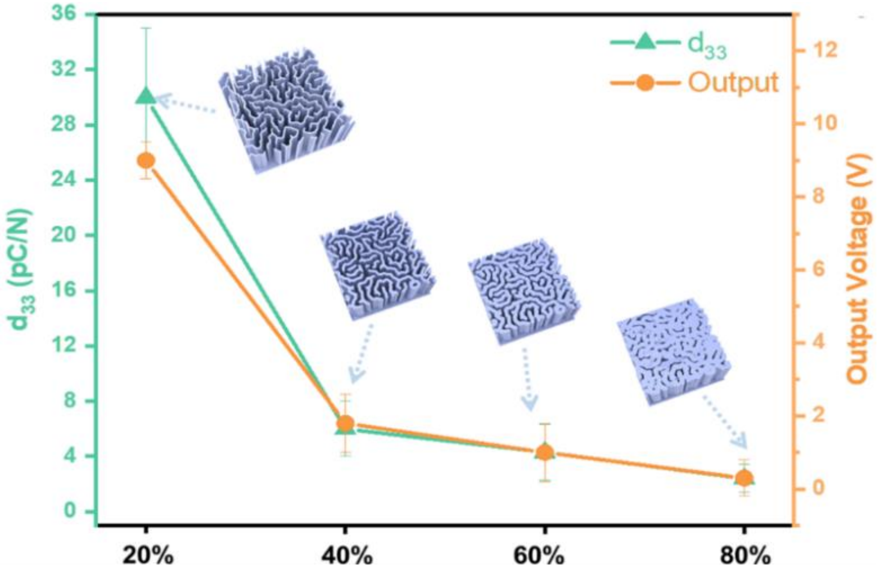

Fig. 2(e): Voltage output and piezoelectric coefficient comparison line for the identical test conditions corresponding to the polymer ratio of different 3D-printed cuttlebone-RS composites.

We thank the reviewer to mention the coupling factor of the samples, we have measured the Rochelle salt composite in 20% volume ratio. The electrical impedance of the composite has been measured by impedance analyzer (Agilent 4294A, Santa Clara, CA, USA). The electromechanical coupling coefficient (k_t) of the piezoelectric material can be defined as the following equation, whereas f_r is resonant frequency, and f_a is anti-resonant frequency.

$$k_t=\sqrt{\frac{\pi fr}{2fa}\times \cot\frac{\pi fr}{2fa}}$$

Based on the tested result in below **Fig. R.12**, the f_r and f_a can be located as 203.3 kHz, and 204.2 kHz, respectively, calculating the k_t (Coupling coefficient/factor) as 9.3%.

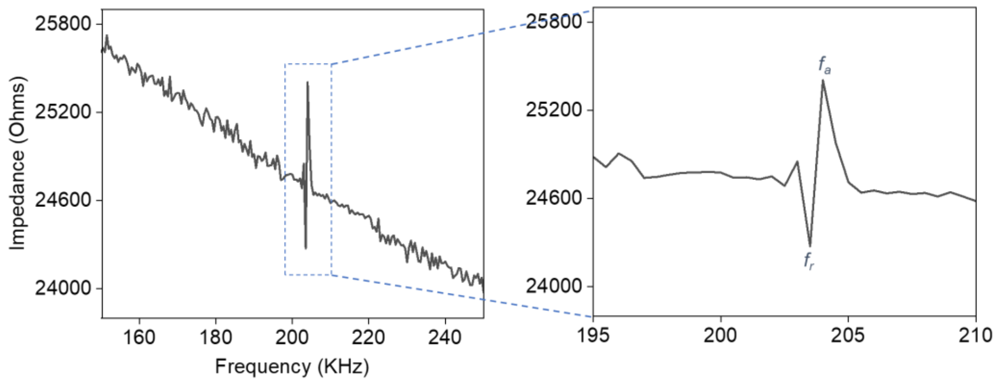

Fig. R.12: Measured Impedance spectrum of the Rochelle salt composite in 20% volume ratio

-> Are there any flaws in the data analysis, interpretation and conclusions? Do these prohibit publication or require revision?

The effective converse [pm/V] and direct [pC/N] d_{33} should be measured on several sample and compared with respect to a relevant chosen parameter (RS loading, grain size, impedance etc.). Output voltage only is not sufficient to qualify piezoelectric properties and samples comparative performances.
This work require revision.

NB: for example, triboelectric effects in some sample could produce quite similar output signals (without any piezoelectricity). Thus, piezoelectricity must be demonstrated systematically (e.g. measuring direct and converse piezoelectric effects).

[Author response]: We are thankful to the reviewer for this question. Continuing on the previous question, the direct and converse d_{33} for Rochelle salt composite in different volume ratio (RS loading) have been tested, which has been described in the previous response.

To demonstrate that the output of the sample is derived from piezoelectric effects rather than triboelectric effects, Similarly, we used a 20g weight to hit the composites at a distance of 5cm vertically in order to measure the output voltage. The results of **Fig. R.13** indicate that, as compared to the more obvious output voltage signal of the 3D-printed RSC, the epoxy and silicone rubber samples did not exhibit any significant voltage output after being hit by the weights. Additionally, we used the MSA-500 laser vibrometer to obtain displacement and phase data as **Fig. R. 14** shown. These illustrations show that Rochelle salt composites exhibit piezoelectric properties.

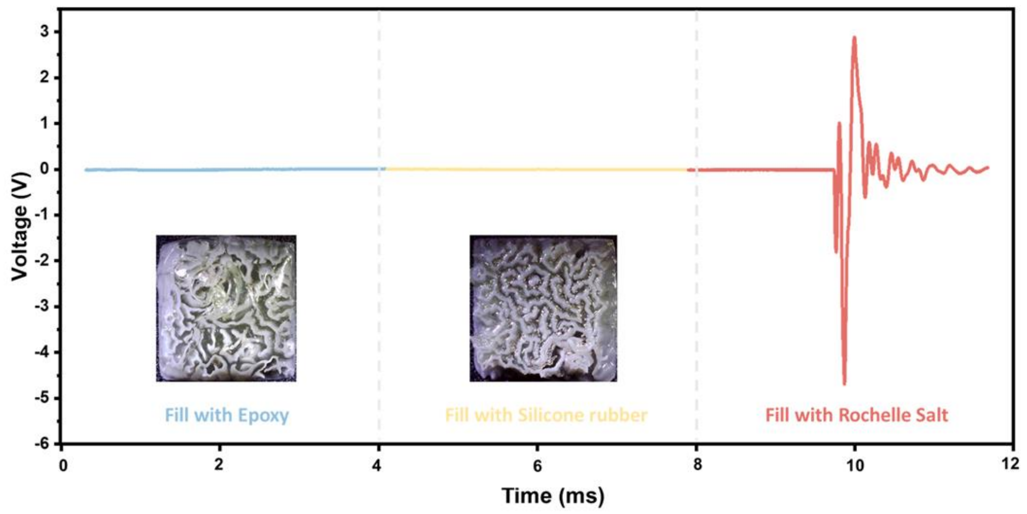

Fig. R.13: Voltage output graphs of 3D-printed Cuttlebone fill with different materials

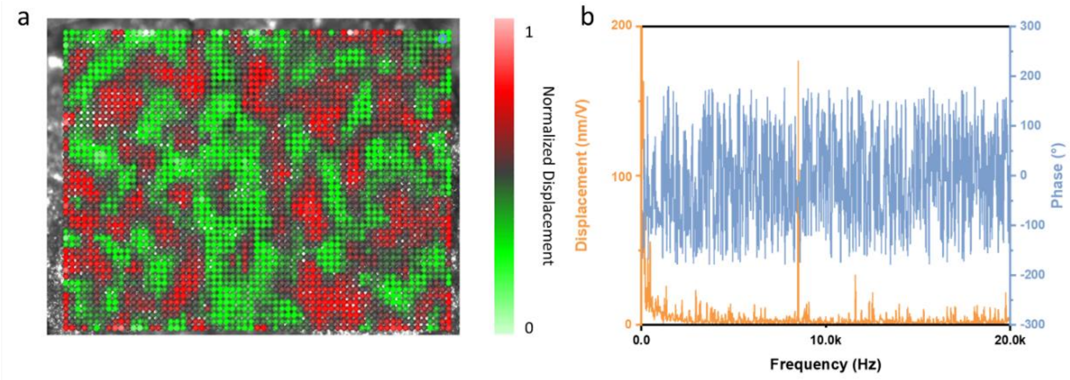

Fig. R.14: Displacement and phase of 3D-printed-RSC measured using a laser vibrometer
(a) Normalized displacement distribution under microscope; (b) Displacement module and phase of 3D-printed-RSC graph

Furthermore, we compared the converse [pm/V] and direct [pC/N] d_{33} by using d_{33} meter (YE2730A, APC international, Ltd., Mackeyville, PA, USA) shown in **Fig. R.9** (according to the last question), and MSA-500 laser vibrometer test machine shown in **Fig. R.10** (according to the last question).

-> Is the methodology sound? Does the work meet the expected standards in your field?

In terms of methodology, the authors should consider additional characterizations (related to piezoelectricity):
- comparison of complex impedance, current or d_{33}, from one sample to another.
- direct and converse piezoelectricity should be highlighted.
- the piezoelectric coupling factor of fabricated samples should also be documented.

[Author response]: We are grateful to the reviewer for pointing this question. For d_{33} comparation from one sample to another, we compared four different

polymer ratios of 3D-printed-RSC composites shown in **Fig. 2(e)**. Each result was collected from the testing of five different samples with the same kind.

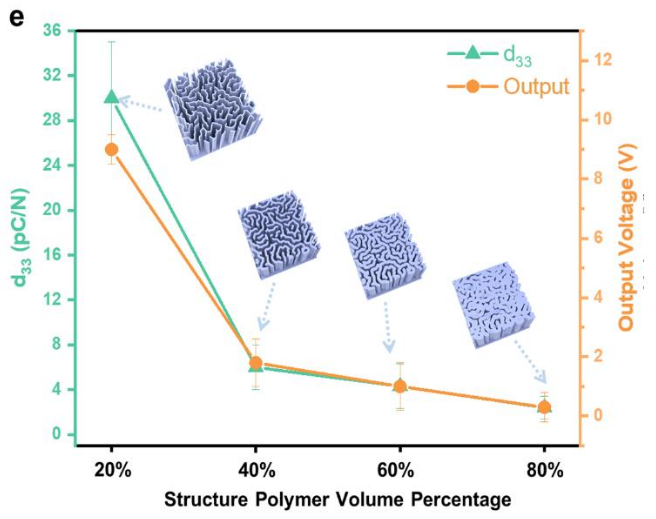

Fig. 2(e): Voltage output and piezoelectric coefficient comparison line for the identical test conditions corresponding to the polymer ratio of different 3D-printed cuttlebone-RS composites

To test the converse piezoelectricity, the static displacements were calculated for several applied voltages by using MSA-500 laser vibrometer from Polytech, from 1V to 10V, under 5 kHz is shown in **Fig. R.11**. The statistic displacement increased with the applied voltage, and we performed a linear fit to the resulting curve, and the slope of the fitted curve is the converse d_{33}. This results in the direct and converse d_{33} values remaining essentially the same.

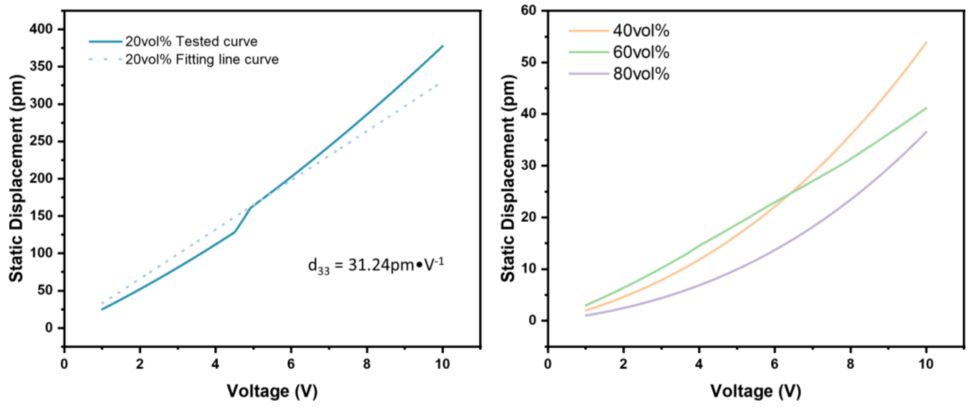

Fig. R.11: Static displacement vs. applied voltage measured on 20%, 40%, 60%, 80% composites.

Continuing on the previous question, we have added the results of direct and converse d_{33} of the Rochelle salt in different volume ratio. And we have tested the coupling factor of the Rochelle salt composite in 20% volume ratio (**Fig. R.15** below). The coupling factor has been determined as 9.3% based on the previous response. The impedance of the composite is between 24300 ohms and 25400 ohms. For Rochelle salt composite in volume ratio of 40%-80%, the

impedance spectrums are hard to tested based on the small quality of Rochelle salt piezoelectric materials in the composite.

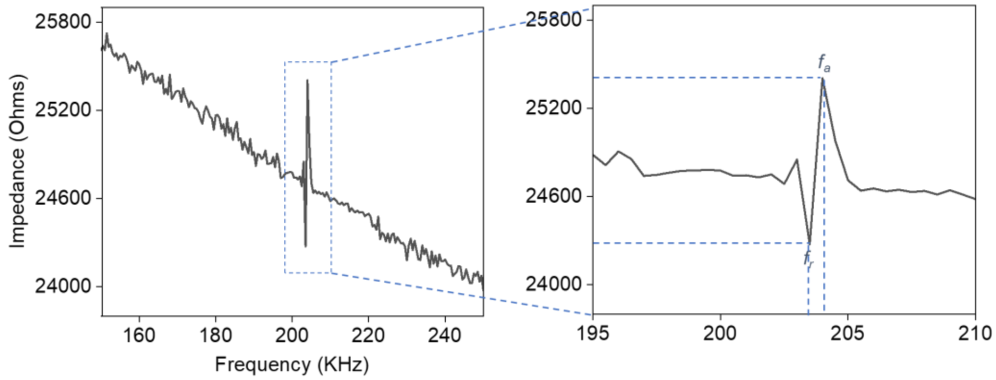

Fig. R.15: Measured Impedance spectrum of the Rochelle salt composite in 20% volume ratio

-> Is there enough detail provided in the methods for the work to be reproduced?

The fabrication process and raw characterizations are well documented in the manuscript and in the supplementary data. Sufficient details are provided regarding reproducibility.

[Author response]: We thank the reviewer for the great comment.

Reviewer #3 (Remarks to the Author):

The authors present newly manufactured multifunctional sensors that are also mechanically strong and thus can be used in knee caps and armors supposedly for sports applications. Rochelle salt, an ecofriendly material is used as a piezoelectric sensing element, which were grown on a 3D printed polymeric scaffoldic structures. However, there are serious flaws in the procedures and test results presented in this work and I recommend that this article should not be accepted for publication in the reputed Nature communications journal.

[Author reply]: We thank the reviewer for the comment.

- Will the work be of significance to the field and related fields? How does it compare to the established literature? If the work is not original, please provide relevant references.
Ans: Not in its current state.

[author reply]: We greatly appreciate the valuable feedback provided by the reviewer. Bionic multifunctional structural materials, which possess characteristics such as light-weight, strength, and perceptibility, have exhibited significant potential in diverse fields including sports, medicine, and aerospace. Nevertheless, the integration of smart monitoring devices with both mechanical protection and piezoelectric induction remains a limited area of exploration. In this study, some innovation points include:

1. Inspired by cuttlefish, a stereolithography-based 3D-printing method was applied to build the artificial cuttlefish bone structures to grow RS crystals for reinforcement smart monitoring devices.
2. The growth mechanism and piezoelectric performance with crystallographic direction of RS crystals inside different 3D printed cuttlebone-inspired structure were studied.
3. Microscale analysis together with FEM simulations by using Comsol Multiphysic, the 3D printed cuttlebone-inspired matrix outperforms the traditional cubic, honeycomb and triangular structure with less stress concentration and strains. And 3D-printed cuttlebone-RS composites growing at 24 hours shows exceptional specific toughness and strength, which is similar to the toughness mechanism in natural cuttlebone.
4. The fabricated RS composite has excellent recyclable and healable properties. The mechanical and piezoelectric performance of the repaired and recycled samples were both tested and showed comparable performances with original structures, which demonstrated the excellent sustainability of applying 3D-printed cuttlebone RS composite.
5. Biomimetic 3D-printed cuttlebone-RS composites are capable of providing superior protection and outstanding piezoelectric sensing capabilities, making them ideal candidates as a smart armor or pads that provides integrated mechanical protection and electrical sensitivity.

- Does the work support the conclusions and claims, or is additional evidence

needed?
Ans: No.

[author reply]: We thank the reviewer's comment. In this study, we report a strategy to grow piezoelectric Rochelle Salt (RS) crystals in 3D-printed cuttlebone-inspired structures for reinforcement smart monitoring devices. The cuttlebone structure was achieved by a 3D printing method using photocurable resin to grow RS crystal composites. The synthesis and the mechanism of piezoelectric performance of RS crystal in the 3D-printed cuttlebone structure were systematically investigated. The optimized printed composites with growing crystals (especially 20 vol% polymer) show excellent specific toughness of 3.125 MPam1/2/gcm-3. In addition to its outstanding mechanical and piezoelectric performance, the growth mechanisms, the recyclability, and repairability of the RS crystals in 3D printed bionic device were studied. The new recycled 3D-printed cuttlebone RS composite can achieve recovery percentage of over 90% in piezoelectric and mechanical properties, illustrating an excellent sustainability of applying RS composite. In a continuous cyclic test, the sample can be repeated close to 7000 times. Moreover, the smart array armor as well as the knee pad based on the 3D-printed cuttlebone-RS composite can be recycled, healed, and achieved for the detection of the location and magnitude of the force experienced by the wearers. These results establish the foundation for new-generation smart monitoring electronics, which shows promising potential in fabricating smart wearable electronics for various applications, such as sports, medicine, military, and aerospace.

- Are there any flaws in the data analysis, interpretation and conclusions? - Do these prohibit publication or require revision?
Ans: There are major flaws in the testing, data analysis as well as fabrication and methodology of the article. This article should not be published in this present state.
1. It is not clear as the shown graphs are piezoelectricity or of a combination of piezoelectricity and triboelectricity. Have the authors checked for this difference in the phenomenon by reversing the electrodes as explained in the article: (Šutka, Sherrell et al. 2020)

[author reply]: We are thankful to the reviewer for pointing this question out. We have studied Šutka, Sherrell et al.[8] paper carefully. Both piezoelectric and triboelectric are mentioned in the paper as tests with output voltage or charge, However, in piezoelectric material, charge is generated by the deformation of aligned polymer chains in a crystallite, while in triboelectricity, opposite charges form on opposing surfaces as a result of contact between two dissimilar materials. Chen et al.[9] also illustrated that piezoelectric materials generate charge by deforming, while triboelectric materials generate charge by rubbing or adhering.
Based on the results of the above two studies, output voltages for different materials inserted inside 3D-printed Cuttlebones, and reverse piezoelectric signal tests to confirm the existence of displacement changes. To eliminate the voltage generated by friction between different materials and electrodes, we printed the cuttlebone first in the same manner. Following that, epoxy, silicon rubber, and RS solutions were each used to fill the 3D-printed Cuttlebone frame,

and electrodes were plated on both sides with E-solder on each sample. The output voltage signal was taken by hitting the composite with a 20g weight at a distance of 5cm vertically. **Fig. R.16** shows that compared to the 3D-printed-RSC more obvious output voltage signal, the samples filled with epoxy and silicone rubber did not show any significant voltage output after being hit by the weights.

Moreover, the converse piezoelectric effect was also achieved by getting the displacement under the input voltage. In order to measure out-of-plane displacement modules and phase changes in the tested samples, the actuating conditions (-10V to 10V sinusoidal input voltage from 0Hz to 20kHz) were applied to the samples which were placed in a laser vibrometer (MSA-500 from Polytec) as **Fig. R.17(b)** presented. The **Fig. R.17(a)** illustrates the normalized out-of-plane displacement distribution, in which the red area exhibits a greater displacement change than the green area, corresponding to the locations of the filled RS crystals and the 3D-printed Cuttlebones. Based on the dynamic response of the 3D-printed-RSCs in the frequency domain, it was confirmed that the composites exhibited a converse piezoelectric behavior.

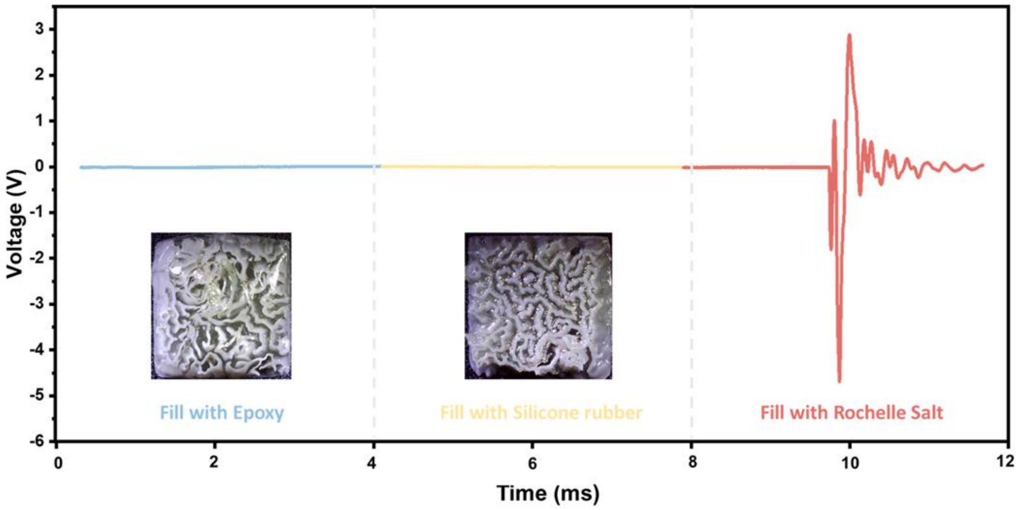

Fig. R.16: Voltage output graphs of 3D-printed Cuttlebone fill with different materials

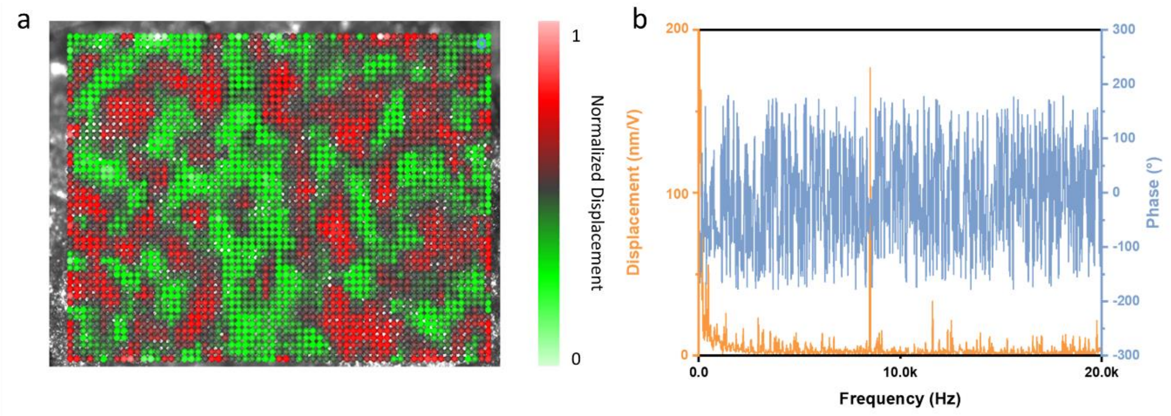

Fig. R.17: Displacement and phase of 3D-printed-RSC measured using a laser vibrometer

(a) Normalized displacement distribution under microscope; (b) Displacement module and phase of 3D-printed-RSC graph

Furthermore, to assure that the measured signal is actually the result of piezoelectric responses and not the result of artificial noise, a voltage from switching polarity tests is measured[10], as shown in **Fig. R.21(a) and (b)**. Upon reversing the polarity of the fabricated device, the shapes of the electrical output curves are reversed, confirming the piezoelectric response.

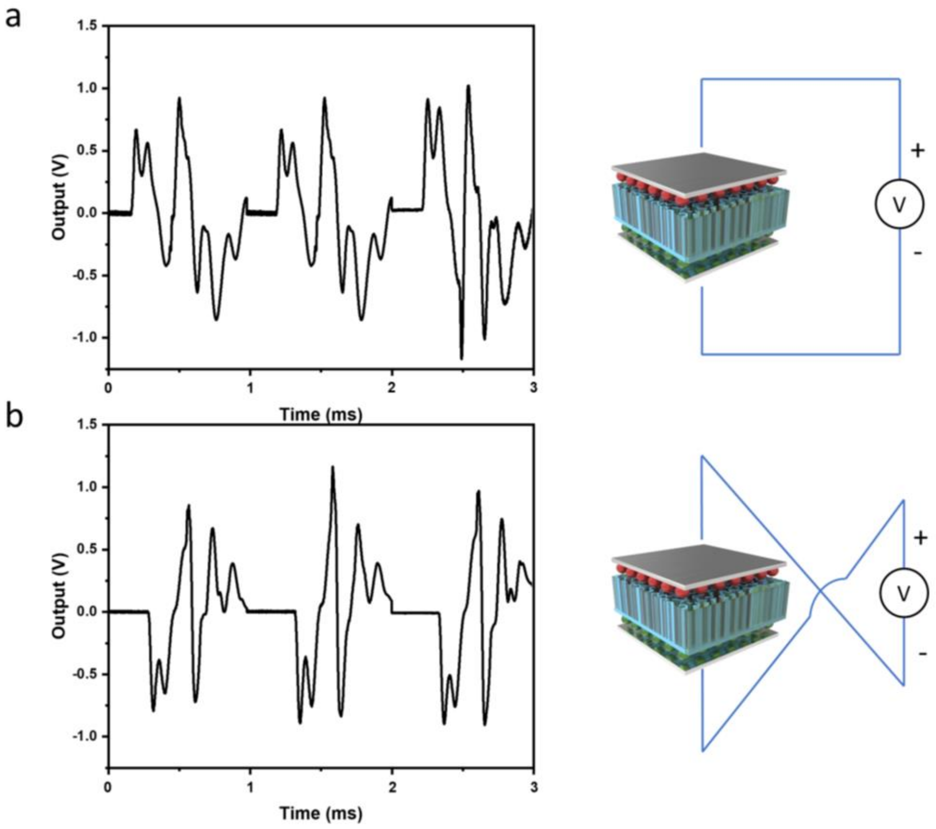

Fig. R.21: (a) The electrical output voltage; (b) The switching polarity tests for output voltage.

2. What are “three different volume ratios of 40%, 60%, and 80% of 3D-printed cuttlebone polymer structures without RS crystal, and with grown RS crystals for 24 hours.” And how are they prepared? This is missing in the methods section.

[author reply]: We are grateful to the reviewer for this question. **Fig. S.4** showed the photos of three different volume ratios of 40%, 60%, and 80% of 3D-printed cuttlebone polymer structures without RS crystal, and with grown RS crystals for 24 hours. The volume ratio here refers to the volume of the cuttlebone structure in relation to the overall cube (10mm × 10mm × 3mm). Due to the fact that the composites are composed of two materials, one being a 3D-printed cuttlebone frame and the other being RS crystals, 40% volume ratio refers to 40% of 3D-printed polymeric cuttlebone frame and 60% of RS. Following that, three different proportions of 3D-printed samples were immersed in the RS crystal solution for 24 hours simultaneously, after which they were removed for

piezoelectric testing. The crystal scale for 3D-printed samples corresponds to 60%, 40%, and 20% shown in the **Fig. S.4**.

Following the reviewers' comments, we have described this preparation process more clearly in the Methods section in the revised manuscript.

3. Fig.1: Where is the XPS curve for the images in 1b, how can you prove that all that is coming from the same sample? What is the proportion of various elements?

[Author reply]: We are grateful that reviewer point out this question. The reviewer might mean EDS instead of XPS as it is related to the SEM images in Fig 1b. To illustrate the proportion of the elements, we have added the updated EDS spectrum curve. Considering EDS analyzes the SEM images in real-time, it is difficult to locate the same part in the tested sample; therefore, we chose the same 3D-printed-RSC composite as analyzed in the paper, but a different part for another EDS analysis. The microstructure and composition of the 3D-printed-RSC were further examined using SEM and energy dispersive X-ray spectroscopy (EDS) shown in **Fig. R.18(a)**. Based on the results, it is evident that the carbon elements are concentrated in the 3D-printed cuttlebone by virtue of the light-curing resin. As the RS crystals grow in the middle of the skeletal gaps, oxygen, potassium, and sodium are mainly concentrated. EDS Spectrum in **Fig. R.18(b)** depicts the expected major elements such as Carbon from the 3D-printed cuttlebone, Oxygen, potassium, and sodium from the RS crystal. **Fig. R.18(c)** illustrates the weight ratio and atomic ratio of four different elements in the tested composite.
In combination with SEM and TEM, EDS is commonly used to perform point sweeps, line sweeps, and surface sweeps of samples, which make it possible to determine the distribution of elements on the surface (in combination with SEM) or in the bulk phase (in combination with TEM) of the sample; XPS, on the other hand, is generally used independently in order to detect surface information, as well as to determine elements' composition, chemical state, and molecular structure. As a consequence, we verified that the elements were obtained from the same sample by EDS, and we analyzed the elemental composition semi-quantitatively.

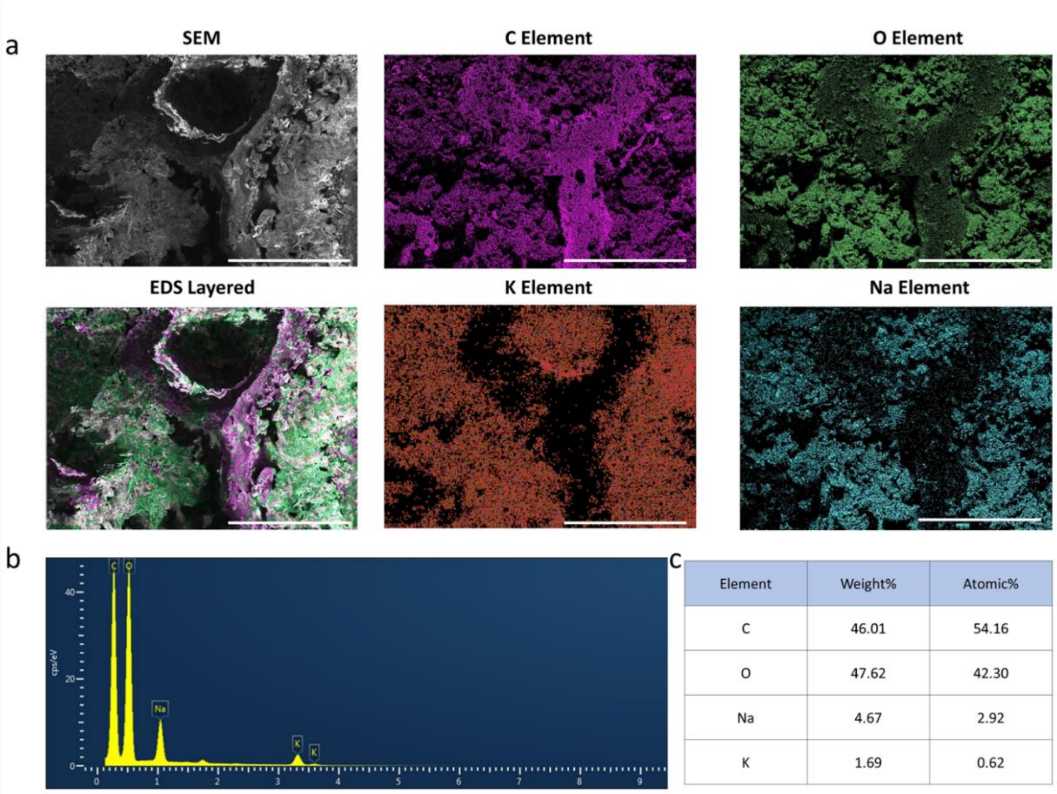

Element	Weight%	Atomic%
C	46.01	54.16
O	47.62	42.30
Na	4.67	2.92
K	1.69	0.62

Fig. R.18: SEM-EDS of 3D-Printed-RSC
(a) SEM photo and EDS element layered photos (scale bars: 500μm); (b) EDS spectrum; (c) The weight ratio and atomic ratio of four different elements.

4. What was the applied mechanical signal for the response shown in Fig.5?

[author reply]: We sincerely thanks to the reviewer for this question. To obtain the input force signals corresponding to the drops of 10g and 5gweights, we placed two flexible force sensors of the same type on the 3D-printed-RSC smart armor. The **Fig. R.19** shows the output of mechanical signals from 16 elements under the impact of weights. The magnitude of the forces on D1 and B3 are 89mN and 41mN, respectively. The mechanical signal derived from the output voltage signal was depicted in **Fig. 5(c) and (d)**, which is consistent with the measured physical input signal.

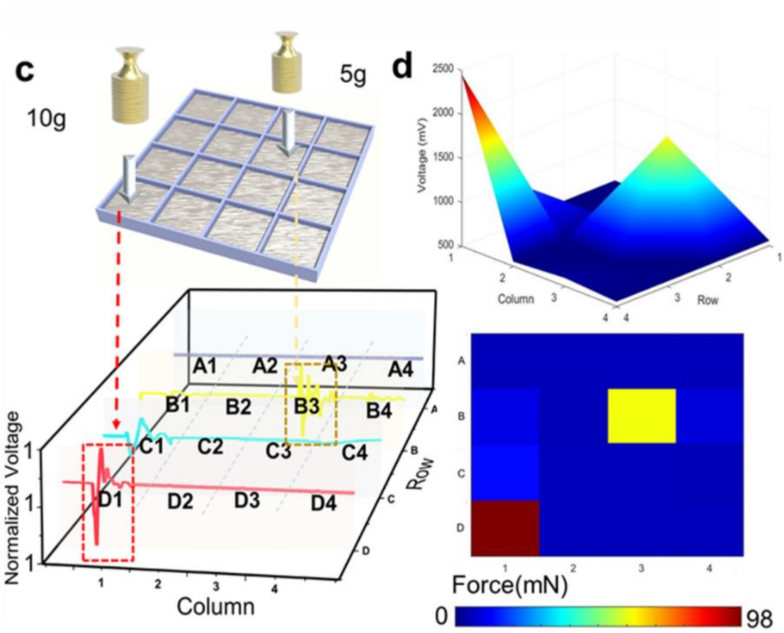

Fig. 5(c): Voltage output waveform of the 16 elements corresponding to the impact and Fig. 5(d): the force distribution derived from the voltage analysis by MATLAB.

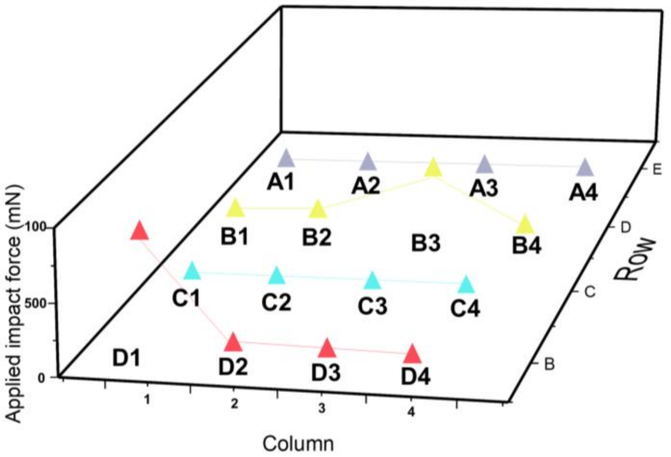

Fig. R.19: Applied impact force of 16 elements of smart armor.

5. How many samples of each kind were tested in the mechanical tests? What were their dimensions? The data presented should include a statically correct amount of tested samples.

[author reply]: We thankful to the reviewer for this question. During the mechanical tests, we used 5 samples for each kind of test, in **Fig. 3(f)** The error bar is also labeled with the average, maximum, and minimum values for flexural strength and fracture toughness for the compared tested samples. For the dimensions, in compress test, the tested samples dimensions are 10mm × 10mm × 7mm; In bending test, we printed composites for 10mm × 5mm × 3mm; In the 3 points bending test with notch, the samples size are the same as the

bending test samples, which are 10mm × 5mm × 5mm, with the notch height of 0.6mm. In the method section of revised manuscript, we have added additional information regarding the composites tested.

6. Through lines 286 and beyond, the authors wrongly claim their process as recyclable, where they are reusing the salt solution for new 3D printed scaffolds. This is not regeneration.

[author reply]: We sincerely thank to the reviewer for careful reading. We corrected the error in this description, and during the experiment, we immersed the 3D-printed-RSC in heated DI water, and the RS in the composite was completely dissolved in the DI water to obtain the recycled RS solution.

7. Many confusing statements are present in the article and need to be corrected. For example, here are a few of them.

[author reply]: We are grateful to the reviewers for their corrections, and we feel sorry for our carelessness. We have revised the article and the supplementary materials and addressed the questions raised as follows.

a) Line145: “Stainless steel<<What?>>weight loadings (5g - 20g) were applied to”??

[author reply]: We thank the reviewer for pointing this out. We have added “steel” in the sentence.

b) Line146: “the 20%-80% composites along the vertical” What is 20vol% and what is 80% in these composites?

[author reply]: **In Fig. S.4**, we illustrated the different ratio in the 3D-printed-RSC composites. The ratio refers to the percentage of the entire square (10mm x 10mm x 3mm) occupied by the 3D-printed cuttlebone. 20vol% refers to 20% 3D-printed cuttlebone and 80% RS crystal in the entire 3D-printed-RSC composites. In the contrast, 80vol% refers to 80% 3D-printed cuttlebone and 20% RS crystal in the entire 3D-printed-RSC composites.

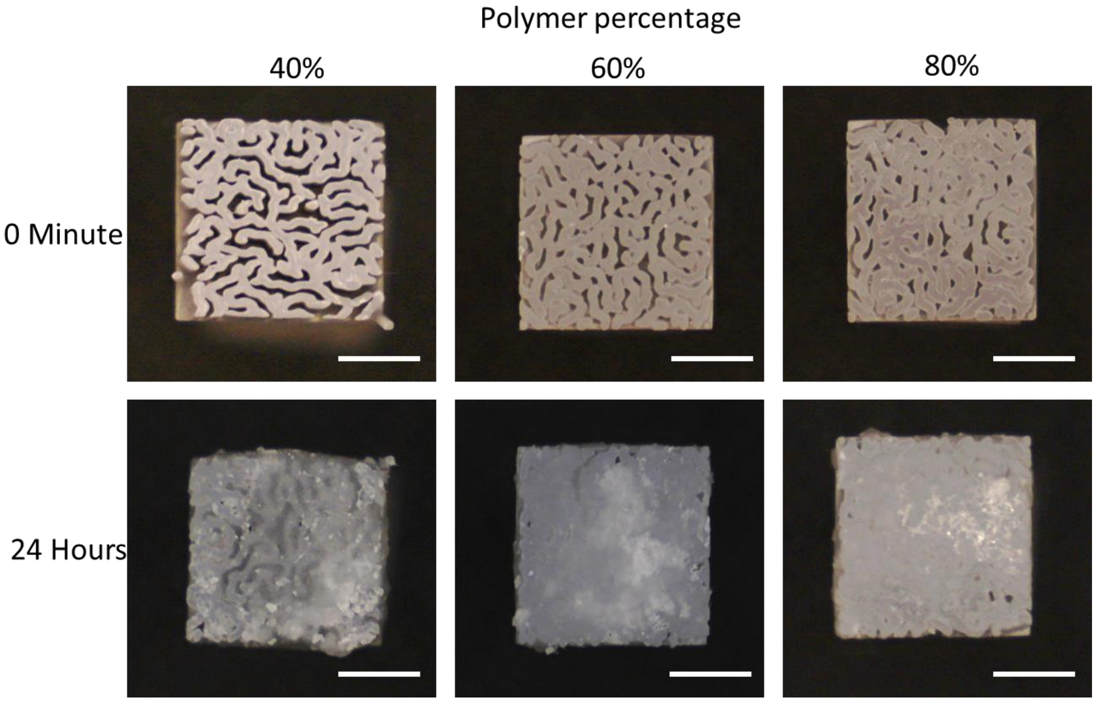

Fig. S.4 Photos of three different ratios of 40%, 60%, and 80% of 3D-printed cuttlebone polymer structures without RS crystal, and with grown Rochelle salt crystals for 24 hours. (Scale bar, 5mm)

c) Line198: "The cuttlebone structure suffers the least deformation and homogeneous force distribution compared to the other three structures when subjected to the same compressive force."

[author reply]: We have changed the description as "When subjected to the same compressive force, the composite with cuttlebone structure exhibits the least deformation and uniform force distribution compared to the composites with other three structures."

a) Line452 in the article: The cuttlebone structure, triangular, cubic, honeycomb structure, and 4 × 4 Array armors with cuttlebone structures in each element

[author reply]: We thank the reviewer for mentioning this sentence. We have changed the description as "All the 3D-printed structures were designed by "SolidWorks" and "Fusion 360" software.

a) Line463 in the article: "For each layer to be able to be attached to the print platform, 2.5 seconds of exposure time was set, followed by 35 seconds of exposure time at the bottom." What does that mean? The procedure is unclear.

[author reply]: We used the Digital Light Processing (DLP) 3D printing technology in our study. The designed 3D model file firstly needs to be sliced into 2-dimensional image format, with each image representing the shape of each layer in the 3D printing process. The resin material is poured into the material tank and the 3D printer's platform (**Fig. S.1**) is gradually raised from the bottom to the top at the end of printing each layer. The printing time (resin curing time) of each layer can be adjusted to ensure that the 3D printer is

controlled to print out the sample in detail. The curing time of 35 seconds was set for the first layer at the bottom to ensure that the resin material can fully adhere to the printing platform and will not fall off, after that the curing time of each layer was set to 2.5 seconds to ensure the printing accuracy and will not be over-cured.

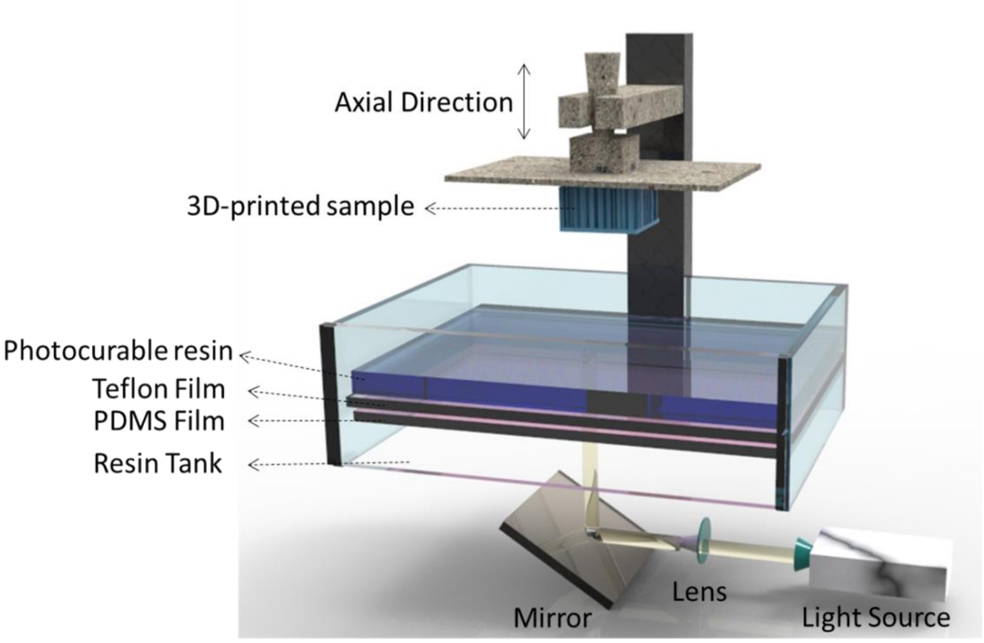

Fig. S.1 The schematic of the DLP-3D printing machine.

8. Please correct Figure S1 title, “Fig. S.3 The CT scanned photos of 20% cuttlebone polymer structure grown RS crystal for 24 hours. (Scale bar, 50mm)” there is only one photo in the figure.

[author reply]: We feel sorry for our carelessness. We have fixed the writing error and corrected the scale bar. ‘Fig. S.3 The CT scanned photo of 20% cuttlebone polymer structure grown RS crystal for 24 hours. (Scale bar, 5 mm)’.

- Is the methodology sound? Does the work meet the expected standards in your field?
Ans: No the methodology is unclear and irreproducible in its current state. The work does not meet the standards in the field of 3D printing. The procedure to 3D print and available commercial resin and then growing Rochelle salt crystals in it doesn’t represent the novelty in 3D printing process at all. Also, growing Rochelle salt crystals in 3D printed scaffolds has already been published (Lemaire, Thuau et al. 2021). Hence the novelty of this work is questionable.

[author reply]: We are thankful to the reviewer for pointing this question out. Dr. Lemaire's research on RS composites[1, 7] studied the growth of RS in paper, wood, and in a 3D-printed basic frame, he has extensively analyzed the piezoelectric properties of the composites. But the mechanical performance of those natural materials and 3d printed lattice structure are not strong enough for armor and knee pads and the growth mechanisms of RS crystal as well as the piezoelectric and mechanical properties are not clear. Based on these

points, we did our work with the intention of adding higher mechanical properties to the composites based on their piezoelectric properties.

A cuttlefish skeleton has to withstand greater pressure because of its survival in the deep ocean, and we were simultaneously inspired by its structure in nature. As a final step, we combined the piezoelectric properties of the RS crystal with the mechanical properties of the cuttlefish bone structure in order to fabricate the 3D-printed-RSC to realize multifunctional applications.

The growth mechanism and piezoelectric performance with crystallographic direction of RS crystals inside different 3D printed cuttlebone-inspired structure were studied in this paper. The optimized printed composites with growing crystals (especially 20 vol% polymer) show excellent specific toughness of 3.125 MPam1/2/gcm-3. In addition to its outstanding mechanical and piezoelectric performance, the growth mechanisms, the recyclability, and repairability of the RS crystals in 3D printed bionic device were studied. The new recycled 3D-printed cuttlebone RS composite can achieve recovery percentage of over 90% in piezoelectric and mechanical properties, illustrating an excellent sustainability of applying RS composite. Moreover, the smart array armor as well as the knee pad based on the 3D-printed cuttlebone-RS composite can be recycled, healed, and achieved for the detection of the location and magnitude of the force experienced by the wearers.

- Is there enough detail provided in the methods for the work to be reproduced? Ans: No. The methodology lacks details. As well as the results in Fig2 are coming from only singular tests, with no proof of repeatability or accuracy of the sensors presented? Do the sensors generate the same signal with the dropping of the same weights multiple times? Is missing in the results and discussions. The FEM analysis and the inputs and model specifications are not clear. What do you mean by X1,X2 and X3 axes? How do they correspond to the global access? How were the properties for the RS that were supplied to the model, obtained? Were they from literature, or did the authors carry out experiment's to determine them? In either cases, all the input parameters and values to the model should be presented in the article.

[Author response]: We are thankful to the reviewer for those comments. In **Fig.R.20** and **R.21** we added the comparison of 3D-printed-RSC output voltage at different frequencies and different forces, as well as tested composite output voltage at 2Hz impact for 8000 cycles instead of singular tests. For each single output test by using weights, we also performed the same impact test with each group of five 3D-printed-RSC samples under the same conditions to obtain the average output voltage, which is marked as an error bar in the **Fig. 2(e)**.

For FEM analysis, the material's position is specified by the Material Coordinate System, which is denoted by the XYZ axes. Therefore, while simulating piezoelectric materials, it is of utmost importance to take its spatial orientation and poling direction into account in order to correctly interpret the material properties. Since the RS crystal itself does not require polarization, herein, we defined that (x_1, x_2, x_3) in a Cartesian reference frame correspond to the (X, Y,

Z) in the global access. In **Fig. S.14**, we described the piezoelectric coefficients of RS, which is obtained by COMSOL Multiphysics® software to collect the material references (**Fig. R.22**). Following the reviewers' comments, we have illustrated this issue in the Methods section in the revised manuscript.

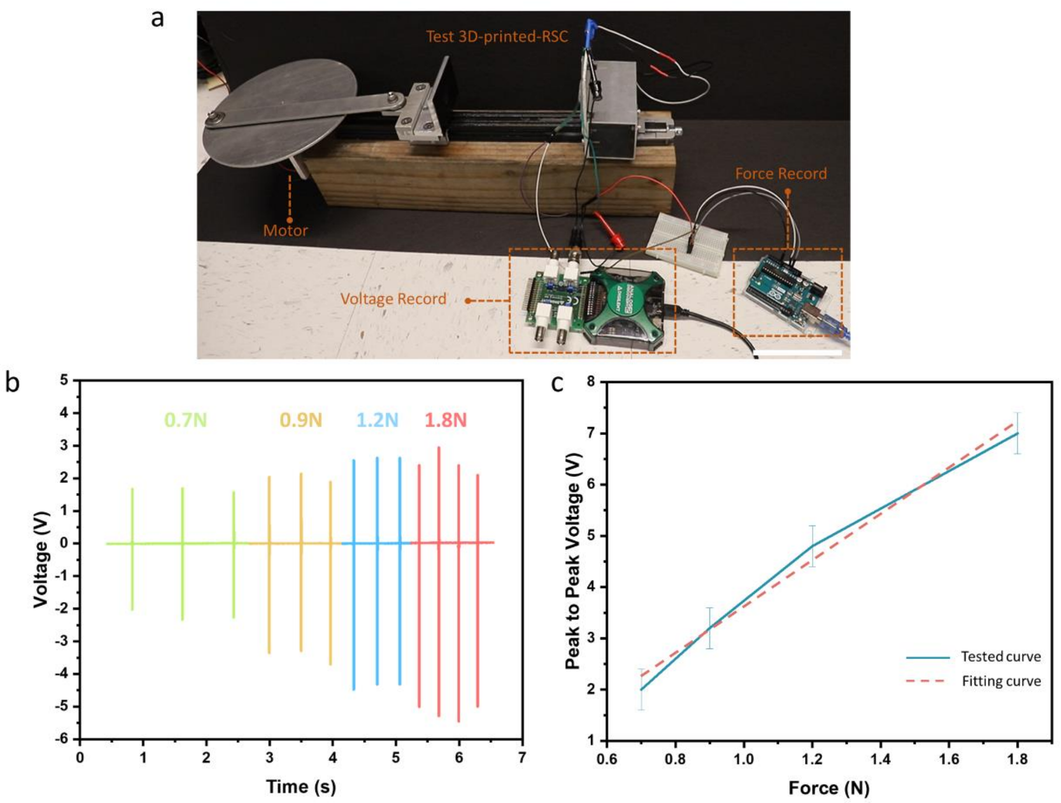

Fig. R.20: Cycle testing performance.
(a) Photo of test setup. (scale bar: 10cm); (b) Piezoelectric output at different frequencies corresponding to different force magnitudes; (c) Peak to peak voltage output at different force.

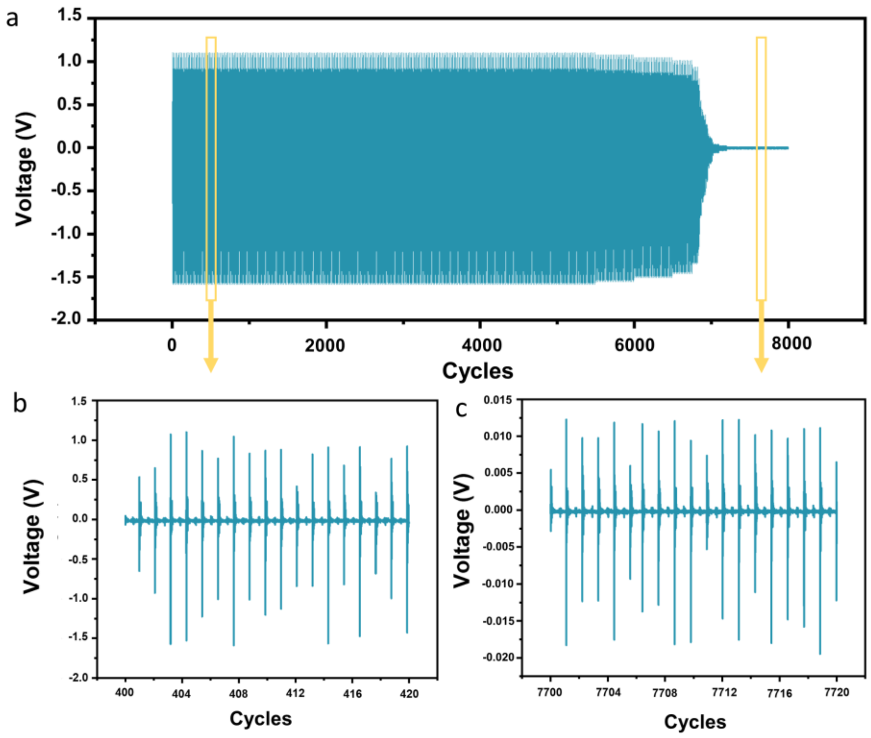

Fig. R.21: 8000 cycles cyclic impact test output voltage graphs
(a) The output voltage over time of 8000cycles cyclic impact test under 2Hz frequency; (b) The graph of zoom in between 400 to 420 cycles; (c) The graph of zoom in between 7700 to 7720 cycles.

[Editorial Note: Figure redacted]

Fig. R. 22: Screenshot of Rochelle salt material contents in COMSOL

References:

Lemaire, E., D. Thuau, J. B. De Vaulx, N. Vaissiere and A. Atilla (2021). "Rochelle Salt-Based Ferroelectric and Piezoelectric Composite Produced with Simple Additive Manufacturing Techniques." Materials (Basel) 14(20).

Šutka, A., P. C. Sherrell, N. A. Shepelin, L. Lapčinskis, K. Mālnieks and A. V. Ellis (2020). "Measuring Piezoelectric Output—Fact or Friction?" 32(32): 2002979.

9.

References:

[1] E. Lemaire, D. Thuau, J.-B. De Vaulx, N. Vaissiere, and A. Atilla, "Rochelle Salt-Based Ferroelectric and Piezoelectric Composite Produced with Simple Additive Manufacturing Techniques," *Materials,* vol. 14, no. 20, p. 6132, 2021.

[2] R. Levitskii, I. Zachek, T. Verkholyak, and A. Moina, "Dielectric, piezoelectric, and elastic properties of the Rochelle salt NaKC 4 H 4 O

6· 4 H 2 O: A theory," *Physical Review B,* vol. 67, no. 17, p. 174112, 2003.
[3] A. D. Snyder, Z. J. Phillips, J. S. Turicek, C. E. Diesendruck, K. B. Nakshatrala, and J. F. Patrick, "Prolonged in situ self-healing in structural composites via thermo-reversible entanglement," *Nature Communications,* vol. 13, no. 1, p. 6511, 2022.
[4] C. Cheng, J. Li, F. Yang, Y. Li, Z. Hu, and J. Wang, "Renewable eugenol-based functional polymers with self-healing and high temperature resistance properties," *Journal of Polymer Research,* vol. 25, pp. 1-13, 2018.
[5] H. J. Sim *et al.*, "Self-healing electrode with high electrical conductivity and mechanical strength for artificial electronic skin," *ACS applied materials & interfaces,* vol. 11, no. 49, pp. 46026-46033, 2019.
[6] H. Cui *et al.*, "Three-dimensional printing of piezoelectric materials with designed anisotropy and directional response," *Nature materials,* vol. 18, no. 3, pp. 234-241, 2019.
[7] E. Lemaire, R. Moser, C. Borsa, and D. Briand, "Green paper-based piezoelectronics for sensors and actuators," *Sensors and Actuators A: Physical,* vol. 244, pp. 285-291, 2016.
[8] A. Šutka, P. C. Sherrell, N. A. Shepelin, L. Lapčinskis, K. Mālnieks, and A. V. Ellis, "Measuring piezoelectric output—fact or friction?," *Advanced Materials,* vol. 32, no. 32, p. 2002979, 2020.
[9] C. Chen *et al.*, "A method for quantitatively separating the piezoelectric component from the as-received "Piezoelectric" signal," *Nature Communications,* vol. 13, no. 1, p. 1391, 2022.
[10] R. Yang, Y. Qin, C. Li, L. Dai, and Z. L. Wang, "Characteristics of output voltage and current of integrated nanogenerators," *Applied Physics Letters,* vol. 94, no. 2, p. 022905, 2009.

REVIEWER COMMENTS

Reviewer #1 (Remarks to the Author):

The authors have made revisions to the manuscript, resulting in some improvement in quality. However, the revised paper still lacks in-depth analysis and the performances should further be optimized.

1.Although relevant experiments have been performed, the relationship between the structure and performance has not been established. In Fig. R.2(a), there is no change in properties with no change in the piezoratio. It seems the size of the sensing structures essentially do not influence the piezoelectric performance. This reviewer doubt whether the test is correct.

2.Facing the question 2 of Reviewer #1, the authors should give an in-depth optimization to combine mechanical properties and piezoelectric sensing performance of the device.

3.For the piezoelectric test, by applying stainless weight loadings (5g - 20g) is not a standard test method, the corresponding data are advised to be retested and replaced in the revised manuscript.

4.If the RS crystals will decompose at approximately 55 °C, the range of sensing temperatures is serious limited. Besides, 7000 cycles cannot meet the requirement of the practical applications.

5.Many response to reviewer’s concerns were not reflected in the revised manuscript.

Reviewer #2 (Remarks to the Author):

The authors have responded to all requests in detail and numerous changes have been made to the manuscript accordingly. Several details about the piezoelectricity of the composite are now clearly explained and detailed. We congratulate the author on this work.

Reviewer #3 (Remarks to the Author):

Accept as is now.

Response letter to reviewer comments

Reviewer #1 (Remarks to the Author):

The authors have made revisions to the manuscript, resulting in some improvement in quality. However, the revised paper still lacks in-depth analysis and the performances should further be optimized.

1.Although relevant experiments have been performed, the relationship between the structure and performance has not been established. In Fig. R.2(a)， there is **no change** in properties with no change in the piezoratio. It seems the size of the sensing structures essentially do not influence the piezoelectric performance. This reviewer doubt whether the test is correct.

[Author reply]: We appreciate the reviewers pointing this out. In the first response, we tested the performance of 3D-printed-RSC structure with only three different size scales by using drop weights. The number of samples is limited for us to find the relationship between the structure and performance. We have improved the experiment by using more samples with five different size scales and cyclic testing was used as the standard test method. As the **Fig. R1** shows, using five different scales, we studied the relationship between structure size and mechanical and piezoelectric performance (Line 133-134, and Fig. S5). **Fig. R1(a)** depicts 3D-printed cuttlebone structures. By keeping the volume of a 3D-printed cuttlebone (CB) as 20% of the volume of a 3D-printed-RSC, the following structures are fabricated and shown from left to right: Structure of a cuttlefish bone after enlargement by two times (2 scale), the original scale, 1/4 scale, 1/16 scale, 1/32 scale. Our model also incorporates a 1/32 scaled down 3D-printed RCB structure that is designed to further mimic the original size of Cuttlefish bones. Moreover, we added structures that were enlarged by a factor of two in order to better understand how the size of structure affects performance. **Fig. R1(b)** displays different scales of 3D-printed-RSC, the optical photos of each scale of 3D-printed-RSC illustrates in **Fig. R1(c)**. Regarding to Zhang et al.[1], the cuttlebone wall thickness is approximately 5-10 µm. Herein, Micro-SLA 3D printers were used to fabricate five different scales of 3D-printed-CB.

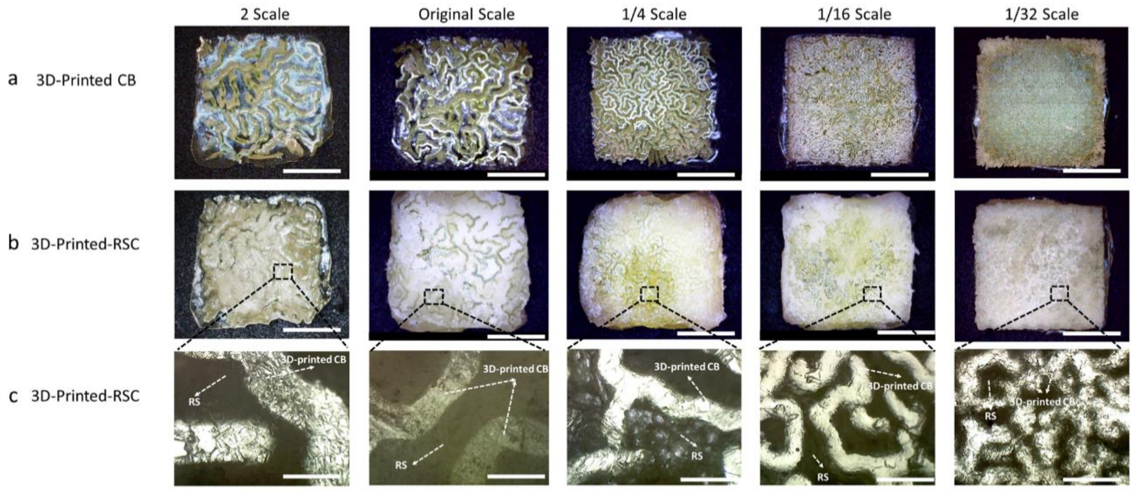

Fig.R1: 3D-printed-RSC with different scales.

(a) 3D-printed CB with same 20% cuttlebone ratio but different scales. (Scale bars: 5mm) (b) 3D-printed-RSC with same 20% cuttlebone ratio but different scales. (Scale bars: 5mm) (c) Microscope photos of 3D-printed-RSC with same 20% cuttlebone ratio but different scales. (Scale bars: 500μm)

Since bending tests are sensitive to specimen geometry and loading rates, the results are very variable. To obtain a clear trend in the relationship between the scales and the mechanical properties, we performed compression tests at different scales of 3D-printed-RSC instead of bending tests. Ten samples of each size were tested to obtain a clear trend. The compressive stress-strain curves of 3D-printed-RSC with different scales are demonstrated (**Fig. R2(a)**) with a buffered downtrend (Line 133 - Line 134, and Fig. S6). In general, the greater the volume reduction, the closer the cuttlefish bone wall thickness is to the actual size, accompanied by higher stress values. **Fig. R2(b)** demonstrates the output voltage waveform of five different scales 3D-printed-RSC generated by continuous impact under 2 Hz instead of using the weight as impact. The piezoelectric properties decrease with an increase in strength illustrates in **Fig. R2(c)**.

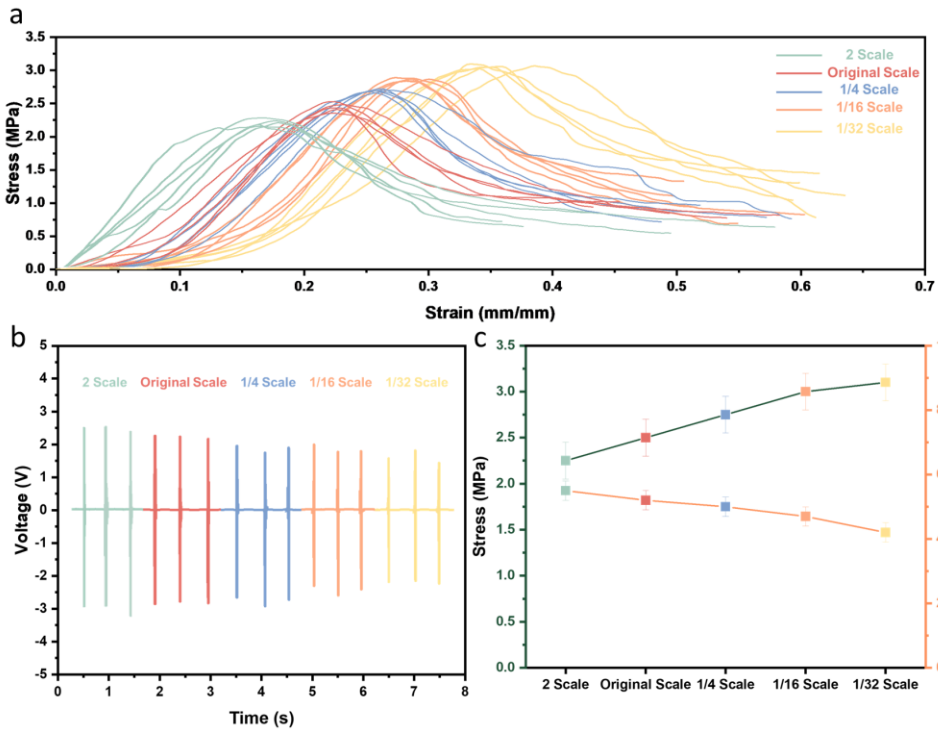

Fig. R2: The relationship between the structure scale size and performance
(a) Comparison of compression properties of different scales 3D-printed-RSC; (b) Piezoelectric output of different scales 3D-printed-RSC under 2 Hz frequency; (c) The relationship of stress and peak-to-peak output voltage among five different scales 3D-printed-RSC.

Fig. R2 shows that the maximum compressive stress increases while the output voltage decreases with decreasing scale size. The results demonstrate the relationship between the structure and performance of the 3D printed RSC structures. In this study, however, large-scale 3D printing is an important consideration with regard to smart knee pads and smart armor. In spite of the

fact that micro-SLA 3D printers can print high resolution samples, the print sizes are very limited. In order to get the best mechanical properties for large 3D-printed structures, we demonstrated the original scale using Phrozen Sonic MINI 4K 3D printers.

2.Facing the question 2 of Reviewer #1, the authors should give an in-depth optimization to combine mechanical properties and piezoelectric sensing performance of the device.

[Author reply]: We are thankful to the reviewer for pointing this question out. A comparison of the mechanical properties of the composite with respect to the piezoelectric output for the four different structures is presented in the **Fig. R3(a)** and **Fig. R3(b)**, with increasing strength, the output voltage decreases (Line 242 – 246, and Fig. S23). In terms of mechanical properties, armor for athletes, knee pads for the elderly and other protective devices are examples of protective devices, and mechanical properties are one of our primary concerns.

Experimental and simulation analyses have been used to determine the mechanical properties of the cuttlefish bone structure, and the results indicate that it has the highest mechanical properties in comparison to the other three structures. In addition, the mechanical properties of cuttlebone are much greater than the properties of the remaining three commonly used structures. The maximum stress of cuttlebone is approximately twice as high as that of the other structures.

Regarding the piezoelectric performance, we acknowledge that the sample does not perform well compared to the rest of the structure. However, as can be seen from **Fig. R3(b)**, under the impact of 2g weight, the piezoelectric output of the sample does not significantly differ from that of the other three composites (~20% lower). The 3D-printed-RSC applications in this study, instead, focus on protective piezoelectric sensors, which can be used as a smart armor to normalize the output voltage in order to obtain the corresponding force in equal proportions throughout the smart armor. The piezoelectric sensing threshold of a smart knee pad for the elderly can be preset, and an alarm device can be activated when the threshold value has been exceeded. Even if the generated output voltage is small, the alarm device can still be triggered by adjusting the valve value.

Taking into account both considerations, we determine that the mechanical performance is more important, and the piezoelectric performance is applicable to the sensing function, leading us to choose the 3D-printed-RSC as the best solution.

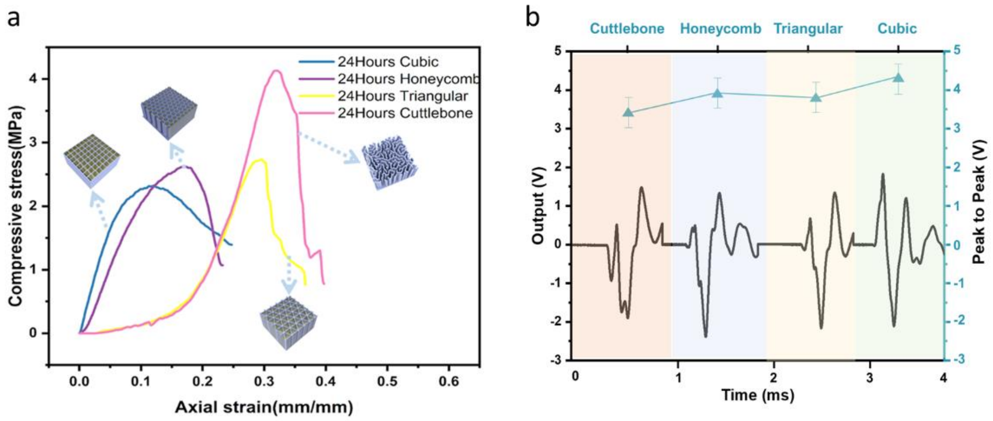

Fig. R3: (a) Comparison of compressibility of RS crystal growth in various structures for 24 hours; (b) Output voltage of Cuttlebone, Honeycomb, Triangular, and Cubic structures.

3.For the piezoelectric test, by applying stainless weight loadings (5g - 20g) is not a standard test method, the corresponding data are advised to be retested and replaced in the revised manuscript.

[Author reply]: We sincerely thanks the reviewer for this suggestion. Based on the previous suggestions about the standard piezo-test (such as cycle test and frequency performance), we adapted **Fig.2 (**corresponding to **Fig. R4)** to the additional test data. **Fig. 2(c)** and **Fig. 2(d)** have been replaced by piezoelectric output at different frequencies corresponding to different force magnitudes, and cyclic impact test output voltage graphs, respectively. Specific descriptions corresponding to the Fig. 2 have been added to the manuscript and supplementary material.

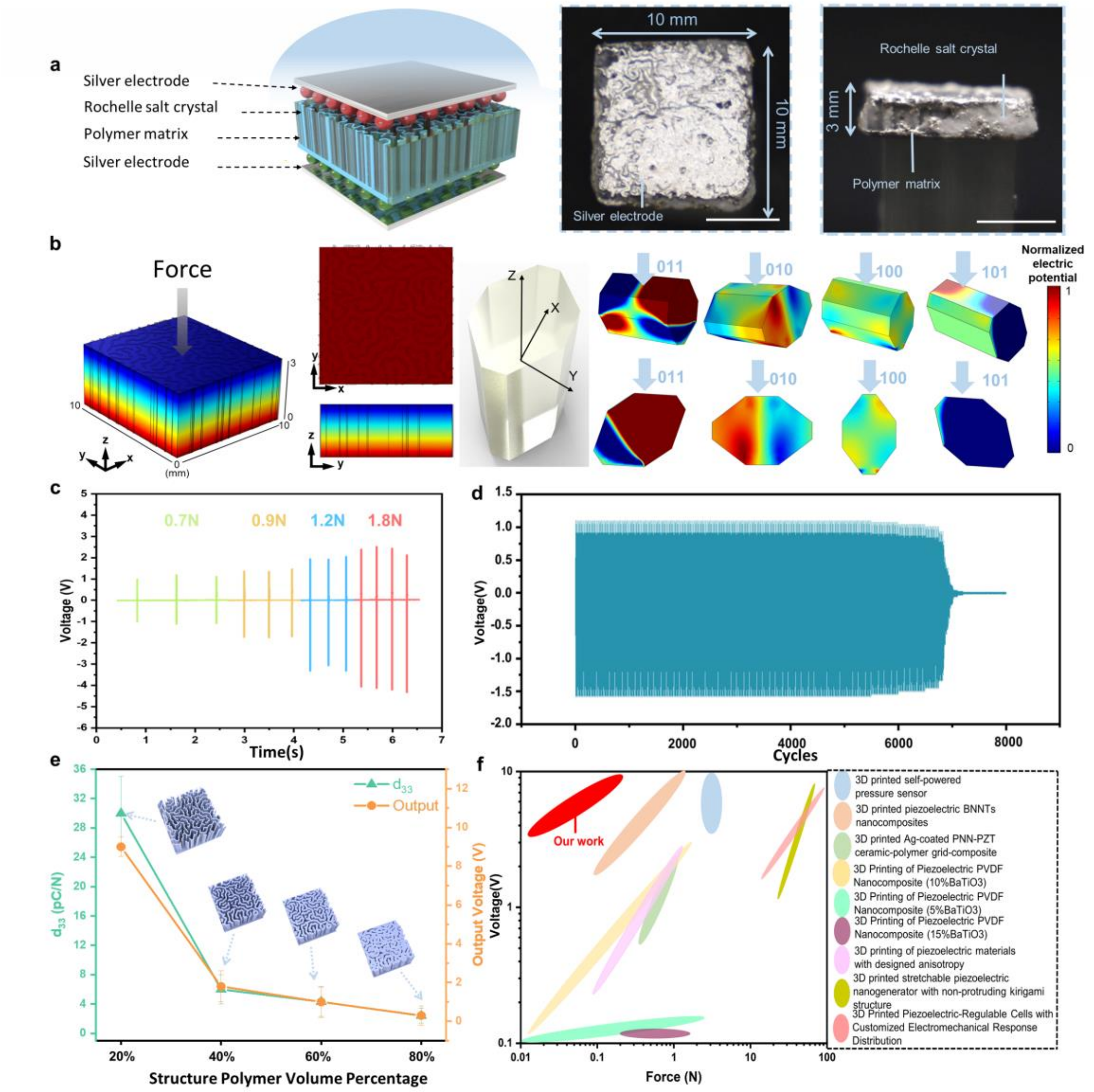

Fig. R4: Corresponding to Fig. 2 in manuscript.

4.If the RS crystals will decompose at approximately 55 °C, the range of sensing temperatures is serious limited. Besides, 7000 cycles cannot meet the requirement of the practical applications.

[Author reply]: We are thankful to the reviewer for this question. There are still wide applications with the sensing temperature ranges, such as wearable protective smart devices for medical, military and sports [2]. In our future research, we will increase the operating temperature range of composites, for example, by adding insulation layers. In An et al. [3]study**,** a hierarchical super-elastic architecture with thermal insulation properties was demonstrated when uniformly distributed porous silica voxels were non-covalently interfaced with polymeric networks. Moreover, the application of the RS crystal in low-temperature working environments can be further investigated with the temperature sensing capability of the crystal [4].

In Karton et al. [5]study, they reported the impact frequency counts from 36.5 to 353 in one season (12 games) of one NFL football athlete may experience

shown in Table. R1, and Cournoyer et al. [6]reported maximum 1400 head impacts can be received per season. Based on the above data, it would appear that cycling 7000 uses as an athlete's smart armor are sufficient for a single season of use (Line 167 - 174). As well as being healable and recyclable, the 3D-printed-RSC composites have an increased service life.

Player Position	Confirmed Impacts		
	Total freq. #	Per Game (ave.± SD)	Per Season (16 games)
QB	73	2.3 ± 2.0	36.5
Rb[a***]	468	14.6 ± 5.4	234
WR	106	3.3 ± 2.9	53
Te[a***]	459	14.3 ± 6.1	229.5
Ol[a***b*]	637	19.9 ± 8.3	318.5
DL[a***b***c*]	706	22.1 ± 8.5	353
Lb[a***]	377	11.8 ± 4.6	188.5
DB	115	3.6 ± 2.5	57.5

Table. R1: Head impact frequency counts for ASF positions. [5]

5.Many response to reviewer's concerns were not reflected in the revised manuscript.

[Author reply]: We are thankful to the reviewer for this question. Changes highlighted in red have been made accordingly in the first and second revised manuscripts and supply materials. The following are specific changes to address each comment:

a: The analysis has been conducted in order to compare the relationship between the size of the structure and the mechanical as well as the piezoelectric performance. Five groups of 3D-printed-RSCs with different scales have been tested and included in the supplementary materials. Relationships have been reflected in the supplementary graphs as well.

b: The piezoelectric test methods have been supplemented with cyclic tests and piezoelectric tests at different frequencies in response to the reviewer's comments. In addition, the **Fig.2** in manuscript has been revised (Page 8). In the manuscript, the weight test image and results have been replaced with the more accurate results resulting from the cyclic test (Page 6-8).

c: We added relevant experiments to the Supplementary Material in order to exclude triboelectric output, and included the corresponding descriptions in the manuscript under "Measurement of piezoelectric response of 3D-printed-RSC" (Page 7).

d: The detailed description of d_{33} measurement has been added to the manuscript under the heading "Method - Piezoelectric response test". Supplementary graphs have been updated with test images (Page 19).

e: Several tests related to the properties of piezoelectric films have been added to the supplementary material. The relevant description has also been included

in the supplementary material and manuscript under "Measurement of piezoelectric response of 3D-printed-RSC." (Page 8).

f: In order to assess the durability of the composite device, we have included a cyclic impact test in the manuscript **Fig. 2** and in supplementary graphs, the description of the increase in useful life was mentioned in “Measurement of piezoelectric response of 3D-printed-RSC” part in manuscript (Page 6-8).

Reviewer #2 (Remarks to the Author):

The authors have responded to all requests in detail and numerous changes have been made to the manuscript accordingly. Several details about the piezoelectricity of the composite are now clearly explained and detailed. We congratulate the author on this work.

[Author reply]: We would like to thank reviewer for taking the necessary time and effort to review the manuscript. We sincerely appreciate all your valuable comments and suggestions, which helped us in improving the quality of the manuscript.

Reviewer #3 (Remarks to the Author):

Accept as is now.

[Author reply]: We would like to thank the reviewer for the thoughtful comments and efforts towards improving our manuscript.

References:

[1] X. Zhang and K. S. Vecchio, "Conversion of natural marine skeletons as scaffolds for bone tissue engineering," *Frontiers of Materials Science,* vol. 7, pp. 103-117, 2013.

[2] V. Patel, A. Chesmore, C. M. Legner, and S. Pandey, "Trends in workplace wearable technologies and connected‐worker solutions for next‐generation occupational safety, health, and productivity," *Advanced Intelligent Systems,* vol. 4, no. 1, p. 2100099, 2022.

[3] L. An *et al.*, "Tailoring thermal insulation architectures from additive manufacturing," *Nature Communications,* vol. 13, no. 1, p. 4309, 2022.

[4] L. Zu *et al.*, "Multiangle, self-powered sensor array for monitoring head impacts," *Science Advances,* vol. 9, no. 20, p. eadg5152, 2023.

[5] C. Karton, T. Blaine Hoshizaki, and M. D. Gilchrist, "A novel repetitive head impact exposure measurement tool differentiates player position in National Football League," *Scientific reports,* vol. 10, no. 1, p. 1200, 2020.

[6] J. Cournoyer, A. Post, P. Rousseau, and B. Hoshizaki, "The ability of American football helmets to manage linear acceleration with repeated high-energy impacts," *Journal of athletic training,* vol. 51, no. 3, p. 258, 2016.

REVIEWERS' COMMENTS

Reviewer #1 (Remarks to the Author):

The authors have satisfactorily answered or addressed all the critics raised the last time and I would recommend the acceptance of this paper.

Response letter to reviewer comments

Reviewer #1 (Remarks to the Author):

The authors have satisfactorily answered or addressed all the critics raised the last time and I would recommend the acceptance of this paper.

[Author reply]: We would like to thank reviewer for taking the necessary time and effort to review the manuscript. We sincerely appreciate all your valuable comments and suggestions, which helped us in improving the quality of the manuscript.
